# Functional characterization and gene expression profiling of *Drosophila melanogaster* short dADA2b isoform-containing dSAGA complexes

**DOI:** 10.1186/1471-2164-14-44

**Published:** 2013-01-22

**Authors:** Tibor Pankotai, Nóra Zsindely, Edith E Vamos, Orbán Komonyi, László Bodai, Imre M Boros

**Affiliations:** 1 Department of Biochemistry and Molecular Biology, University of Szeged, Középfasor 52, H-6726, Szeged, Hungary; 2 Institute of Biochemistry, Biological Research Center, Temesvári krt. 62, H-6726, Szeged, Hungary

**Keywords:** ADA2, SAGA complex, Histone acetylation, Microarray, GCN5

## Abstract

**Background:**

ADA2 proteins, together with ADA3, SGF29 and GCN5 form the acetyltransferase module of GNAT-type histone acetyltransferase complexes. ADA2b is present in the SAGA complex, which plays roles in various chromatin-related processes via histone H3 modifications and by other mechanisms.

**Results:**

In this report we present findings showing that during *Drosophila melanogaster* development two dADA2b isoforms (dADA2bS and dADA2bL) - which differ in their C-terminal domains - are expressed at various levels. Genetic complementation experiments indicate that dADA2bS alone can support development but cannot fully complement *dAda2b* mutations. In the presence of dADA2bS, the SAGA-specific histone H3 acetylation level is partially restored in *dAda2b* mutants. Comparison of whole transcriptome profiles of *dAda2b* null and *dAda2bS* transgene-carrier *dAda2b* null larvae indicates partial overlap between affected genes. mRNA levels corresponding to selected genes which are either up- or down-regulated in *dAda2b* mutants are altered by dADA2bS expression to different extents, ranging from complete restoration to wild type levels to no restoration at all. The short (dADA2bS) isoform of dADA2b seems to be more capable of restoring lost dSAGA functions that cause mRNA level up-regulation than those that lead to decreased mRNA levels.

**Conclusions:**

The data presented here are in accord with results of genetic complementation experiments, and support the hypothesis that different isoforms of dADA2b contribute to the functional variations of dSAGA multiprotein HAT complexes.

## Background

GCN5-containing histone acetyltransferase (HAT) complexes play roles in different molecular processes by affecting nucleosome structure via histone modifications. The SAGA (Spt-Ada-Gcn5-acetyltransferase) complex harbors the GCN5/KAT2 (general control non-derepressible 5/lysine acetyltransferase 2) catalytic subunit and the ADA2 (alteration/deficiency in activation) adaptor protein [1,2], among several other constituents. ADA2 itself has no catalytic activity. It physically interacts with GCN5 and regulates its HAT activity [3-5]. In metazoa there are two ADA2-type adaptors: ADA2b is a subunit of SAGA, while ADA2a is a subunit of the ATAC (Ada Two-A-containing) acetyltransferase complex [2,6-9]. The interacting regions between the human (h) hGCN5 and hADA2b proteins have been mapped to the SANT domain-containing central region of hADA2b, but single amino acid changes in the C-terminal region of hADA2b were also found to interfere with hGCN5 activity [10]. Following the identification of the *Drosophila melanogaster dAda2b* gene, its ability to direct the synthesis of two dADA2b isoforms was recognized [11,12]. We refer to these proteins of 555 and 418 amino acid residues as dADA2bL and dADA2bS, respectively. The existence of two dADA2b isoforms might add to the functional complexity of dSAGA. Studies of *dAda2b* mutants reported so far were carried out using *dAda2b* alleles which affected the production of both dADA2b isoforms [11,12]. These reports demonstrated the essential function of *dAda2b* in *Drosophila melanogaster* development and histone H3K9 and H3K14 acetylation, but provided no information on whether the two gene products are functionally equivalent or whether they have distinct roles regarding these and/or other functions. We sought an answer to this question by generating genetic constructs which permit the study of the function of an individual dADA2b isoform *in vivo*. Here we show that the two isoforms of dADA2b are expressed at different levels during *Drosophila* development, and present data on the *in vivo* function of dADA2bS. We show that dADA2bS alone partially restores viability and histone H3 acetylation levels of *dAda2b* mutants. By comparing transcriptome profiles using microarray of *dAda2b* and *dAda2bS* transgene-carrier *dAda2b* larvae, we found that dADA2bS expression alone altered specific mRNA levels to different extents. Significantly, in *dAda2bS* transgene-carriers the genes which were either up- or down-regulated in *dAda2b* null mutants displayed a shift in their expression level towards that of the wild type control. Our data suggest the preferential involvement of dADA2bS in a dSAGA variant with negative regulatory function.

## Results and discussion

### Two isoforms of dADA2b are expressed at different levels during *Drosophila* development

**Figure 1:**
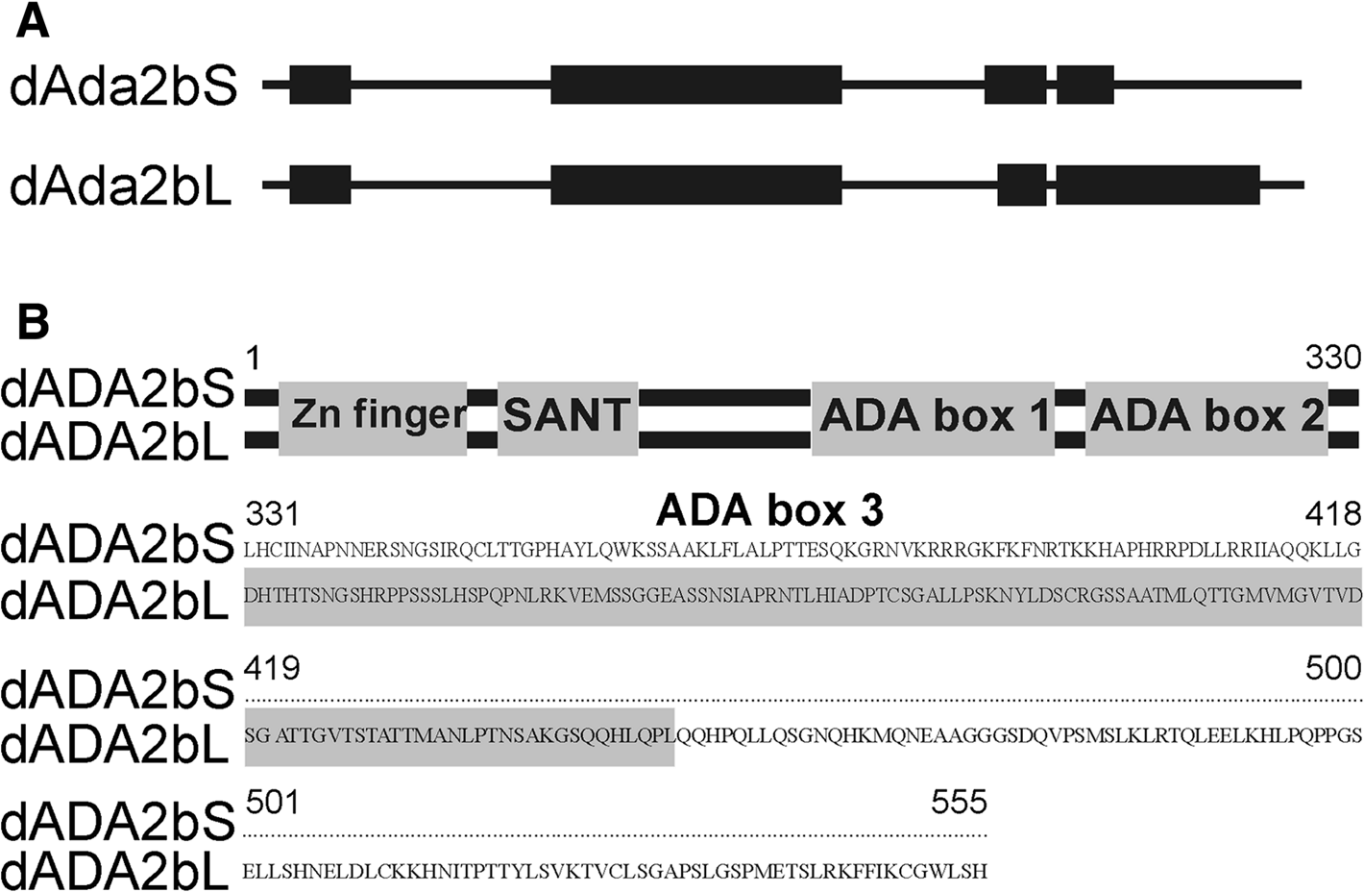
**Schematic structures of the *dAda2b *gene and dADA2b proteins. A**: The structure of two alternatively spliced *dAda2b* messages. Black boxes indicate the position of exons. **B**: Alignment of dADA2bS and dADA2bL protein sequences. The N-terminal 330 amino acid region is represented by lines and boxes indicating the position of conservative protein motifs. The amino acid sequences of the C-terminal regions from amino acid 331 are aligned. The region corresponding to ADA box 3 in ADA2bL is shown in gray.

The structure of the *dAda2b* gene (CG9638) suggests that it can give rise to two mRNA forms, which share their first two exons but are different in exons 3 and 4 (Figure 1A). Consequently, the encoded two isoforms of dADA2b are identical in their N-terminal parts, but differ in their C-terminal regions. The shared parts of the two proteins harbor a zinc finger-like ZZ and a SANT domain, characteristic of all known ADA2 proteins, and two of three so-called ADA boxes (Figure 1B) [1]. A third ADA box is present only in the longer isoform of dADA2b.

**Figure 2:**
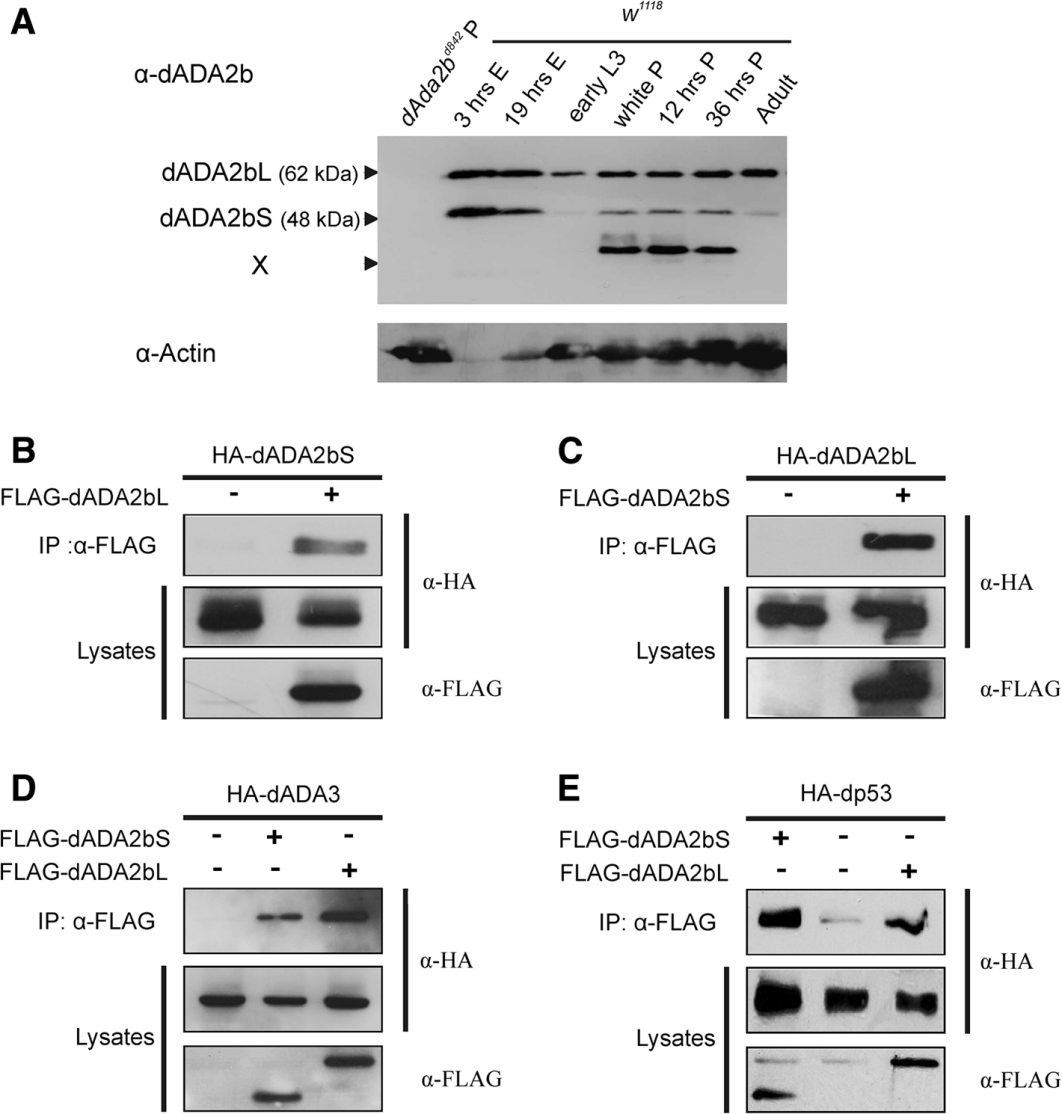
**Co**-**immunoprecipitation of dADA2b isoforms with dADA3 and dp53. A**: Immunoblot of *dAda2b* null (*dAda2b*^*d842*^left lane) and wild type (*w*^*1118*^) animals developed with antibodies specific for dADA2b (top) and actin (bottom). An additional dADA2b antibody immunoreactive band is indicated by X. **B and C**: FLAG-dADA2bL co-immunoprecipitates with HA-dADA2bS and FLAG-dADA2bS co-immunoprecipitates with HA-dADA2bL. **D and E**: Both FLAG-dADA2bL and FLAG-dADA2bS co-immunoprecipitate with HA-dADA3 and HA-dp53. In each panel the epitope tag-containing proteins co-expressed in S2 cells are indicated in the upper part. On the left the immunoprecipitated sample (IP) and the lysates (twice) are shown. The antibodies used for developing the blots are shown on the right.

Using primers which permit the amplification of cDNAs specific for either of the two mRNA forms, we detected both mRNA variants in *D. melanogaster* by RT-PCR (data not shown). In agreement with this, using dADA2b-specific antibodies raised against an N-terminal peptide of the protein we detected two isoforms of dADA2b at different stages of *Drosophila* development (Figure 2A). In denaturing gels the two forms migrate as 62 kDa and 48 kDa proteins. These molecular mass values correspond well to the expected mass of the dADA2bL and dADA2bS isoforms, respectively. The amounts of detectable dADA2b isoforms vary at different stages of *Drosophila* development (Figure 2A). dADA2bS is detectable at highest levels in embryos, and at lowest levels in the mid-larval stage. We noticed that despite the use of protease inhibitors during sample preparations, a fast migrating protein recognized specifically by the dADA2b-specific antibody was present in samples obtained from pupae (Figure 2A. indicated by X). In samples obtained from *dAda2b* null pupae (*dAda2b*^*d842*^) this protein was completely absent, similarly to the two dADA2b isoforms (Figure 2A lane 1). Therefore we find it unlikely that this band corresponds to an immunoreactive protein unrelated to dADA2b. A clarification of the relationship between this protein and dADA2bS and L isoforms, however, requires further studies.

In earlier biochemical separation of dGCN5-containing complexes, dADA2b proteins with different molecular masses were detected in embryonic nuclear extract fractions corresponding to 1.8-2 MDa complexes [1]. As each of these dADA2b forms co-fractionated with TBP, TAF9 and TAF10 proteins, this observation may suggest that several dADA2b isoforms are components of either the same or related multiprotein complexes. To explore this possibility we wanted to determine whether dADA2b isoforms formed heterodimers and interacted with dADA3, another component of ADA2b-containing HAT complexes. Since earlier we found that mutations of the *dAda2b* gene affected dp53 function [12], and physical interaction between dADA2b and dp53 was demonstrated [2], we were also interested to learn whether dADA2b isoforms show differences in interaction with dp53. To detect protein-protein interactions we expressed one of the dADA2b isoforms with N-terminal FLAG and the other with HA epitope tag in S2 cells, and performed co-immunoprecipitations using epitope-specific antibodies. Co-precipitation of dADA2bS with dADA2bL and vice versa indicated *in vivo* physical interaction between the two isoforms (Figure 2B and C). Furthermore, both dADA2b isoforms co-precipitated with dADA3 (Figure 2D) as well as with dp53 (Figure 2E). Thus, the tested protein-protein interactions suggested the possibility of dimer formation between the two dADA2b isoforms but did not reveal different abilities of dADA2b isoforms to participate in interactions with dADA3 or dp53.

Taken together, these data indicate that *dAda2b* gives rise to at least two protein isoforms, dADA2bL and dADA2bS. The two isoforms are present at different levels during *Drosophila* development. Furthermore, the two isoforms interact with each other and with dADA3, another subunit of GCN5-containing HAT complexes. These observations are in line with earlier observations indicating that in embryo extracts different dADA2b forms are present in the same or similar sized complexes, most probably in dSAGA.

### The expression of dADA2bS isoform partially rescues *dAda2b* lethality

**Figure 3:**
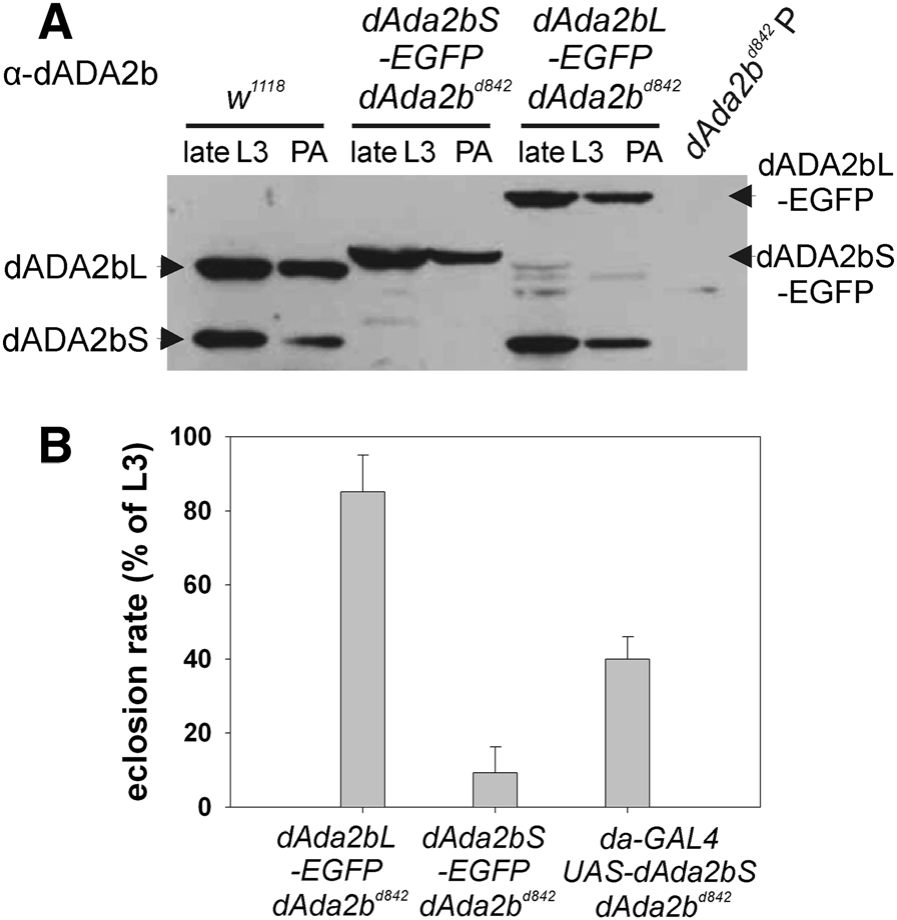
**Expression of dADA2b isoforms in transgene**-**carrier *dAda2b *larvae and rescue ability of *dAda2b *transgenes. A**: dADA2b protein expression in wild type (*w*^*1118*^), *dAda2b*^*d842*^ null mutant and *dAda2b* transgene-carrier *dAda2b*^*d842*^ animals. The genotypes are indicated on top. Transgenes carrying EGFP-fused dADA2b isoform were expressed from the native *dAda2b* promoter. Protein extracts of third instar larvae and pharate adults were separated on SDS-PAGE and immunoblot was prepared using dADA2b specific polyclonal antibody. The positions of native dADA2bS and dADA2bL isoforms are indicated on the left, that of the EGFP tagged forms of the isoforms are shown on the right. Note that the native dADA2bS isoform is also produced in *dAda2bL-EGFP* transgene-carriers, due to alternative splicing of the mRNA. **B**: Rescue of *dAda2b*^*d842*^ mutants by transgenes expressing dADA2b isoforms. Transgenes carrying EGFP-fused dADA2b isoform were expressed from the native *dAda2b* promoter, while the expression of *UAS-dAda2bS* transgene was driven by *da-GAL4* driver, as indicated. The graph shows the percentage of larvae eclosed as adults in each genotype tested.

In order to study the function of individual dADA2b isoforms we generated transgenic lines expressing dADA2bL or dADA2bS with EGFP fused to their C-termini. The transgenes were constructed by fusion of genomic and cDNA sequences in such a way that the expression of the EGFP-tagged proteins could be directed either by the *dAda2b* promoter or by a GAL4 driver due to a UAS sequence inserted in front of the promoter. The expression of the EGFP-tagged dADA2b isoforms is clearly detectable in extracts of transgene-carrier larvae (Figure 3A). The immunoblots also reveal that in *dAda2bL-EGFP* transgene-carrier animals, in addition to the EGFP-tagged long isoform, the shorter form of dADA2b is also produced. This is due to the genomic origin of the transgene and alternative splicing of the transcript.

Next we studied the potential of dADA2b isoforms to complement *dAda2b* mutation. *dAda2b*^*d842*^ is a null allele resulting in lethality in pupal stages [12]. In contrast, 10% of pupae of homozygous *dAda2b*^*d842*^ mutants carrying a single copy of *dAda2bS-EGFP*, and 85% of those carrying a single copy of *dAda2bL-EGFP* completed development and emerged as full-fledged adults (Figure 3B). The incomplete rescue by the *dAda2bL-EGFP* transgene was surprising since carriers of this can express the short isoform as well (Figure 3A). We reasoned that the EGFP tag might interfere with the function of the protein. As the EGFP tag could similarly affect *dAda2bS-EGFP* transgene function, we constructed an untagged *dAda2bS* transgene and studied its rescue ability. Ectopic expression of dADA2bS without an EGFP tag significantly increased the eclosion rate of transgene-carrier *dAda2b* null animals, as 40% of larvae emerged as adults (Figure 3B). However, despite the high level of dADA2bS expression by the transgene-carriers (Additional file 1: Figure S1), we never observed a complete rescue by the short isoform of dADA2b alone. Furthermore, we noticed that adults rescued by the *dAda2bS* transgene had a short lifespan (data not shown).

In summary, from these data we conclude that the dADA2b short isoform can partially provide *dAda2b* functions essential for the development of morphologically normal animals. The incomplete rescue by dADA2bS might suggest that complexes containing this isoform only cannot perform all dSAGA functions perfectly.

### The involvement of dADA2bS isoform in histone H3 acetylation

**Figure 4:**
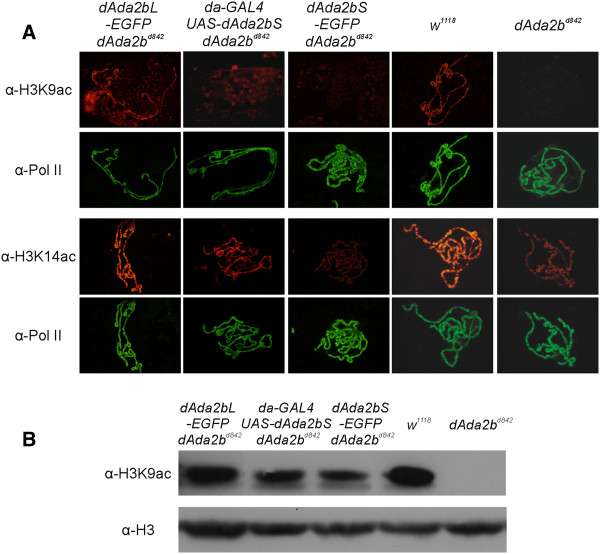
**The effect of *dAda2b *transgenes on histone acetylation. A**: Immunostaining of polytene chromosomes of wild type (*w*^*1118*^), *dAda2b* null (*dAda2b*^*d842*^), and *dAda2b* transgene-carriers (null mutant for endogenous *dAda2b)* with anti-histone H3K9ac and H3K14ac antibodies. Images of the same polytene chromosomes stained with a Pol II large subunit specific antibody 7G5 are shown as staining controls. Genotypes are indicated on the top. Transgenes carrying EGFP-fused dADA2b isoform were expressed from the native *dAda2b* promoter, while the expression of *UAS-dAda2bS* transgene was driven by *da-GAL4* driver, as indicated. Primary antibodies were as indicated on the left. **B**: Comparison of histone H3K9ac and histone H3 levels in larval extracts of the same genotypes shown in **A**. The same filter used for detection of H3K9ac was re-probed with H3- specific antibodies.

In *dAda2b* mutants acetylation of nucleosomal histone H3 at K9 and K14 is severely decreased due to the functional impairment of dSAGA, the dADA2b-containing GCN5 HAT complex [11,12]. We thus found it interesting to determine the activity of complexes containing only dADA2bS in this respect. Polytene chromosomes of *dAda2bL-EGFP* transgene-carrier *dAda2b*^*d842*^ L3 larvae displayed similar staining intensity with antibodies specific for H3K14ac or H3K9ac as chromosomes of wild type animals (Figure 4A). This indicated that, in accord with the observed almost complete rescue, *dAda2bL-EGFP* transgene-carrier larvae produce acetylation competent HAT complexes. In contrast, chromosomes of *dAda2bS-EGFP* transgene-carriers revealed significantly weaker dSAGA-specific acetylation signals (Figure 4A). The transgene directing the expression of untagged dADA2bS, which was more potent in rescue experiments, was also more effective in restoring H3 acetylation. Immunodetection of H3K9ac in extracts of *dAda2b* mutants and transgene-carriers also indicated that dADA2bS complemented at least partially the dSAGA-specific H3 acetylation in *dAda2b* null mutants (Figure 4B). Thus, cells containing only the short isoform of dADA2b produce acetylation-competent HAT complex(es); however, their activity towards nucleosomal histone tails seems to be reduced. It is noteworthy that despite the reduced levels of H3K9 and K14 acetylation, a significant fraction of *dAda2bS* transgene-carrier animals completed development and reached adult stage.

### Transcriptome analysis of dADA2bS isoform expressing larvae

The complementation of *dAda2b* (*dAda2b*^*d842*^*)* mutation by the *dAda2bS* transgene indicated that this isoform can partially restore development. In order to answer the question whether the partial functional rescue was the result of an overall decrease in gene transcription or the differential expression of specific genes, we performed transcriptome analysis. We compared the mRNA profiles of wild type, *dAda2b* null mutants and the *dAda2bS-EGFP* transgene-carriers in *dAda2b*^*d842*^ background. mRNA samples were obtained from late L3 larvae synchronized to anterior spiracle eversion and hybridized to cDNA microarray (Affymetrix Drosophila Genome 2.0 Array) containing 18 000 target sequences. Hybridizations were performed using three independent biological samples. The analysis of microarray data in respect to wild type and *dAda2b* null comparisons has been described [13]; here we analyse the effects of dADA2bS expression.

**Figure 5:**
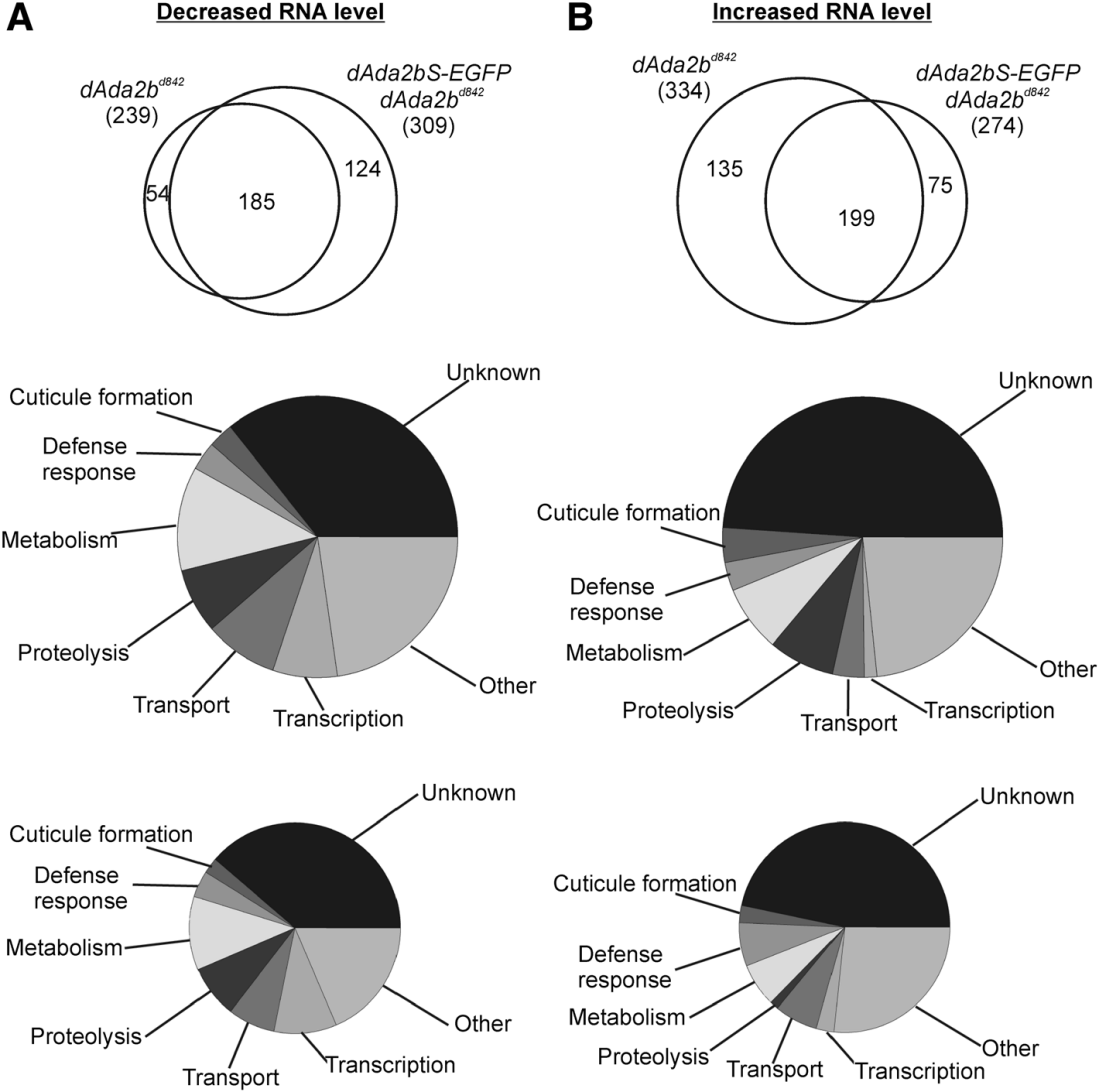
**Gene expression changes in *dAda2b *null *(dAda2b***^***d842***^**) and *dAda2bS****-****EGFP *transgene**-**carrier *dAda2b***^***d842 ***^**(dADA2bS) larvae. A**: The upper Venn diagram shows the overlap among those genes of *dAda2b* null and dADA2bS expressing larvae which show less than 50% of wild type expression. The numbers of affected mRNAs are indicated. Underneath, the affected genes shown on the top are grouped according to GO categories. In the middle are those genes (309) which show decreased expression in dADA2bS larvae. The bottom panel shows the GO categories of those genes (124) which have a decreased mRNA level in dADA2bS larvae only. **B**: As in part **A**, but for genes displaying a higher mRNA level in *dAda2b* null and dADA2bS larvae. Genes which showed at least two-fold increase in mRNA levels are shown only.

We found that in *dAda2b* larvae the levels of 239 mRNAs were reduced by more than 50% of their levels in control samples (*w*^*1118*^) [13] (Figure 5A, Additional file 2: Table S1). The simplest explanation for this could be that this group of genes requires the function of the dSAGA HAT module for transcription, and in absence of dADA2b they are transcribed less effectively. Surprisingly, in dADA2bS samples (*dAda2bS-EGFP* transgene-carrier *dAda2b*^*d842*^ mutants) a higher number of mRNAs (309) were present at less than 50% levels compared to wild type samples (Figure 5A). The overlap between the groups of genes down-regulated in *dAda2b* null and in *dAda2bS* transgene-carriers is high: 77% (185/239). This suggests that for effective transcription of these genes the long isoform, or alternatively both isoforms of dADA2b are required. Another group of genes (124 transcripts) was identified in which genes are down-regulated when only dADA2bS is present but expressed normally in loss of both dADA2b isoforms (Figure 5A, Additional file 2: Table S1). The response of these genes suggests that dADA2bS-containing complexes could act as their negative regulators. This may indicate that dADA2bS may also be present in complexes affecting transcription negatively.

We demonstrated earlier that the absence of *dAda2b* results in increased transcription of several genes [13] (Figure 5B, Additional file 2: Table S1). In fact, the number of mRNAs showing increased signals in *dAda2b* mutants exceeded the number of those which were decreased (334 versus 239). The overlap between the genes with increased mRNA levels in *dAda2b* null mutants versus dADA2bS rescued conditions is 60% (199/334). In contrast, 73% of those genes represented with a higher mRNA level in animals having only dADA2bS were also overexpressed in the *dAda2b* mutant (199/274). From a different perspective: only 40% (135/334) of genes up-regulated in *dAda2b* null mutants were restored by dADA2bS close to control level (Figure 5B, Additional file 2: Table S1). Taken together, the increase of mRNA levels in absence of *dAda2b* is the result of a derepression type of mechanism. The presence of dADA2bS alone prevents this in 40% of the affected genes.

Genes which display altered mRNA levels in *dAda2b* mutants expressing dADA2bS-EGFP (dADA2bS for short) were classified according to Gene Ontology (GO) enrichments (Figure 5 middle). Similarly, we compared the GO categories of those which show altered expression in the presence of dADA2bS, but are not affected in the *dAda2b* mutant (Figure 5 bottom). The distribution of these two groups of genes among GO categories is very similar concerning both the activated and the repressed genes. Among the genes which show increased expression only in the presence of dADA2bS (75 genes), those belonging to GO groups *proteolysis* and *cuticle formation* are underrepresented (compare the middle and bottom pie diagrams in Figure 5B). On the other hand, the representation of genes belonging to GO categories *defense response* and *transport* is somewhat higher among those genes activated only in dADA2bS. No difference is observable in the distribution according to GO categories between those groups of genes repressed in dADA2bS or in the group of genes which was affected by only dADA2bS (Figure 5A middle and bottom panel). It is noteworthy that genes related to *transcription* are represented in higher numbers both among those repressed in dADA2bS and dADA2bS only, compared to those activated in either of these.

### The effect of dADA2bS isoform on the transcription of selected genes

In order to explore whether alterations in the expression of individual genes can uncover specific patterns we analysed the expression of those genes which had the highest hybridization signals (signal values between 100 and 4000 in wild type samples) from the sets of genes affected. In order to investigate the possibility that the different dADA2b isoform containing dSAGA complexes may have diverse functions, we included *dAda2bL-EGFP* transgene-carriers into this microarray analysis. In this respect *dAda2bL-EGF*P transgene-carriers might be considered as a phenocopy of a *dAda2b* hypomorphic mutant. As described above, the *dAda2bL-EGFP* transgene allowed the production of the short dADA2b isoform as well, but resulted in only partial phenotypic rescue, most probably due to the EGFP tag (Figure 3).

**Figure 6:**
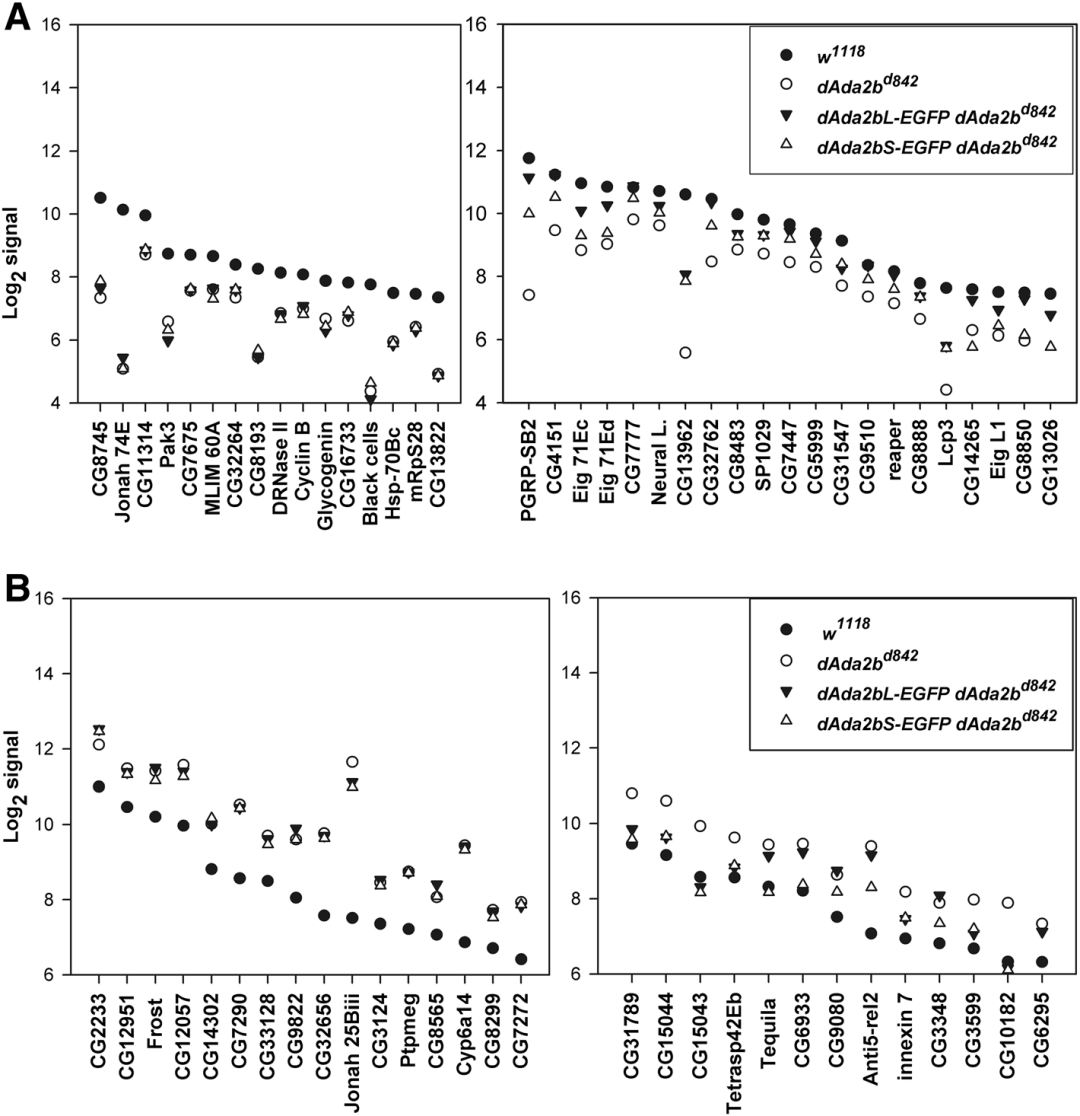
**Changes of mRNA levels of specific genes in *dAda2b***^***d842 ***^**mutant and in transgene**-**carrier larvae. A**: The graphs show RNA levels detected by microarray hybridization in wild type control (*w*^*1118*^), *dAda2b* null (*dAda2b*^*d842*^) and *dAda2b* transgene-carrier animals (*dAda2bL-EGFP dAda2b*^*d842*^, *dAda2bS-EGFP dAda2b*^*d842*^). Only those transcripts which had a strong hybridization signal and showed at least two-fold decrease in *dAda2b* null versus wild type samples are indicated. The RNAs are grouped according to changes of their levels in *dAda2bS* transgene-carriers, as detailed in the text. **B**: The graphs show RNA levels as described in Figure 6A, but only those transcripts which showed at least two-fold increase in *dAda2b* null versus wild type samples are indicated. The RNAs are grouped according to changes of their levels in *dAda2bS* transgene-carriers as detailed in the text.

As expected, in samples obtained from animals expressing an EGFP-tagged isoform of either dADA2bS or dADA2bL, most of the affected genes had RNA hybridization signal intensities situated between the signals of mutant and wild type samples (Figure 6). Significantly, in carriers of either transgene only very few genes were represented by lower or higher RNA signals than in the wild type or null mutant. This is a strong indication of the good quality of our hybridization data. A comparison of signal intensities observed in the four genotypes (*w*^*1118*^, *dAda2b*^*d842*^, *dAda2bL-EGFP dAda2b*^*d842*^, *dAda2bS-EGFP dAda2b*^*d842*^) allowed the classification of genes into groups (Figure 6). In the case of a group of genes down-regulated in null mutants, neither of the transgenes could restore the mRNA levels (Figure 6A). In the case of a large number of *dAda2b* dependent genes, however, the RNA levels were restored to some extent by both or one of the transgenes. The presence of the *dAda2bL-EGFP* transgene resulted in higher hybridization signals than the presence of the *dAda2bS-EGFP* for all of these genes (Figure 6A). This is in line with the observation that the *dAda2bL-EGFP* transgene had a better rescue ability than the *dAda2bS-EGFP*. Practically, for all genes which were represented with decreased mRNA level in *dAda2b* null animals, the expression of *dAda2bS* only was less effective in restoring the wild type mRNA level than the *dAda2bL* transgene (which produces both isoforms).

On about half of the genes which were up-regulated in *dAda2b* mutants, the two transgenes had virtually identical effects: neither could restore the wild type RNA levels (Figure 6B). In the case of the other half of the genes which were up-regulated in *dAda2b* mutants, the expression of either *dAda2b-EGFP* transgene resulted in a decrease in the mRNA level, causing a shift towards the wild type level in transgene-carriers. Intriguingly, for several genes (*Tequila, CG6933, CG9080, Anti5-rel2, CG3348*) the expression of the short dADA2b isoform alone resulted in mRNA levels closer to the wild type than the expression of both isoforms from *dAda2bL-EGFP* (Figure 6B).

## Conclusions

*Drosophila* SAGA harbors one of the two fly ADA2-type adaptors, dADA2b, while dADA2a is present in the ATAC HAT complex [1,7]. Within the yeast (y)SAGA ADA2 and ADA3 are believed to play a role in extending the lysine specificity of GCN5 HAT [4]. In accord with this function, yADA2 physically interacts with yGCN5 [5]. The SANT domain of yADA2 was mapped as a region critical for the interaction and the N-terminal half of this domain was found to be required for effective histone acetylation by yGCN5 in the context of SAGA [14]. In *Drosophila* too, the absence of dADA2b makes dSAGA an incompetent nucleosomal histone H3 acetyltransferase [11,12].

We present here biochemical and genetic evidences that the two isoforms of dADA2b are expressed in different quantities during *Drosophila* development. It might be surprising that despite the detection of two different forms of *dAda2b* messages in earlier studies [1,11] the functions of the two protein isoforms have not been studied. Since dADA2bS has only a short unique peptide segment distinguishing it from dADA2bL, discriminating between the two isoforms might be difficult by mass spectrometric determination of dSAGA constituents. The antibody we used in this study for dADA2b detection is highly specific and the complete absence of both dADA2b isoforms from extracts of *dAda2b* null animals (Figure 2A and 3A) proves that both proteins are *bona fide* products of *dAda2b*.

Our analysis of *dAda2bS* transgene-carriers provides information on the *in vivo* function of the short dADA2b isoform. The partial phenotypic rescue of *dAda2b* null mutants by *dAda2bS* transgenes proves that the shorter isoform alone can contribute to dSAGA functions required for the completion of development. The decreased viability of adults possessing only dADA2bS suggests functional deficiencies, which might be related to dSAGA function in neuronal development [15]. The dSAGA-specific histone H3 modification pattern of animals expressing only dADA2bS indicates a reduction in the level of H3K14ac and particularly in that of H3K9ac (Figure 4). The high level of dADA2bS expression in the transgene-carriers makes it unlikely that the amount of dSAGA complexes would be limiting. Rather, the HAT activity of dSAGA complexes containing only dADA2bS seems to be generally decreased. A change in activity of a C-terminally truncated dADA2b form is in accord with our recent observation that the C-terminal regions of dADA2 proteins play important roles in targeting them to GCN5-containing complexes [16].

Microarray analysis of total RNA profiles revealed that the mRNA levels corresponding to several genes affected by *dAda2b* mutation were partially restored in *dAda2b*S transgene-carriers. Significantly, the levels of those mRNAs, which were either up- or down-regulated in *dAda2b* null mutants, were changed towards the wild type control in *dAda2bS* transgene-carriers (Figure 6). Furthermore, the short dADA2b isoform alone seems to be more potent to restore mRNA levels of genes up-regulated in null mutants.

For a large number of genes we observed that in dADA2bS expressing animals the mRNA levels were close to those detected in *dAda2b* null mutants, so dADA2bS could not restore the normal expression levels of these genes. In the case of a smaller number of genes the mRNA levels in *dAda2bS* transgene-carriers were between those detected in wild type and *dAda2b* samples. The intermediate mRNA levels of these genes are indicative of a partial dSAGA function. Perhaps the most interesting are those few genes for which the presence of dADA2bS resulted in mRNA levels close to the wild type (Figure 6B). All these genes are represented with increased RNA level in null mutants, but in the presence of the short dADA2b isoform their expression is kept at a lower level. The expression of dADA2bS alone results in a decreased level of mRNA for more genes than the expression of none of the dADA2b isoforms (Figure 5A). One explanation for these observations could be that the negative function of dADA2b-containing complex(es) is restored better if at least the short isoform is present, than if none.

The microarray data unfortunately does not provide information on which step(s) of transcription is affected when only dADA2bS is expressed. SAGA complexes have several structural modules, to which specific functions can be assigned [17]. The ADA2 type proteins belong to the GCN5-HAT module, but this does not exclude the possibility that they modify SAGA function through mechanisms other than histone acetylation. For example mutations affecting WDA and dADA2b proteins cause similar phenotypes though the former is part of the deubiquitinating module [18]. Furthermore, the acetyltransferase function of SAGA could affect gene expression by several mechanisms including promoter specific modification, global histone acetylation, factor acetylation, acting at PIC formation or at elongation [19]. The various SAGA functions explain the observations that up- and down-regulated genes are detected in comparable numbers in *dAda2b* mutants. The phenotype and histone acetylation pattern of animals expressing only dADA2bS indicate that with the short dADA2b isoform dSAGA complexes with reduced activity are formed. Whether the activity changes of these complexes affect the expression of genes selectively and differently at specific stages and/or conditions remains to be revealed. Our data on the alterations of the transcriptome in the presence of dADA2bS alone suggest that the use of distinct ADA2b adaptor isoforms could be a way to achieve gene-selective effects.

## Methods

### Drosophila melanogaster strains, transgene constructions and genetic crosses

Fly stocks were raised at 25°C on standard *Drosophila* medium. The *dAda2b*^*d842*^ null allele has been described [12]. The isogenized *w*^*1118*^ strain used here as control has been described [20]. Mutant chromosomes were kept over TM6c Tb, Sb balancer chromosomes.

The *dAda2bS-EGFP* and *dAda2bL-EGFP* transgenes were constructed in *pBluescriptKS* (Fermentas) and inserted into pUASP plasmid with *KpnI* and *NotI*. The two constructs have the same 5^′^ and middle region generated by PCR amplification on genomic templates using the *Ada2bgene-Ada2bgeneL* and *Ada2bRI-Ada2bBHI* primer pairs. The 3^′^ ends were amplified without the translational STOP signals by using the *Ada2bS3*^′^ and *Ada2bL3*^′^*BamHI-Ada2bNcoI* primers and joined to the 5^′^ and middle region using *NcoI*. The EGFP coding sequence was transferred to the 3^′^ ends of the genes encoding dADA2bS and L isoforms from *pEGFPN3* (Clontech) using *BamHI* and *NotI* restriction endonucleases. The used primer sequences were as follows:

*Ada2bRI*: GCA TGA ATT CAT GAC CAC AAT CGC GGA TTT; *Ada2bBHI*: CGA TGG ATC CCC GAC AGC TAT CCA A; *Ada2bNco*: CCA TAT GGC CAT GGC AAG; *Ada2bgene*: TTT AAT CCT GAC CAC CGC T; *Ada2bgeneL*: CAG GGT GGG TCG ATT ATG TTG; *Ada2bS3*^′^*BamHI*: GGA TCC TCC AAG ATG TTT TTG CTG; *Ada2bL3*^′^*BamHI*: GGA TCC GTG GCT CAG CCA GCC GCA. The transgenic *Drosophila* lines were established by embryo microinjection with pUASP vector containing the EGFP-tagged *dAda2b* genes and a Δ2-3 transposase source plasmid, followed by genetic crosses. The generation of *dAda2bS* cDNA construct has been described [12].

For rescue experiments the following genotypes were used: w/w; +/+; *P{dAda2bL-EGFP}dAda2b*^*d842*^/*P{dAda2bL-EGFP}dAda2b*^*d842*^, w/w; *P{dAda2bS-EGFP}*/*P{dAda2bS-EGFP}*; *dAda2b*^*d842*^/*dAda2b*^*d842*^. In experiments using *dAda2bS* cDNA rescue construct the genotype of animals was: w/w; +/+; *P{UAS-dAda2bS} dAda2b*^*d842*^/*P{daGal4}dAda2b*^*d842*^. To calculate rescue efficiency the percentage of larvae eclosed as developed adults was determined in three independent experiments, each involving at least 150 animals for every genotype.

For construction of recombinant plasmids for protein expression in S2 cells the coding sequences of *dAda2bS*, *dAda2bL*, *dAda3* and *dp53* were cloned into the Gateway cloning entry vector pENTR3C. From the obtained entry clones, the inserts were recombined into destination vectors containing either N-terminal FLAG or HA affinity tag and suitable for expression in S2 cells (Invitrogen Gateway® system).

### Western blot

For protein analysis on immunoblots, total protein samples from animals of the indicated genotypes and developmental stages were separated on SDS-PAGE and transferred to nitrocellulose membrane. Membranes were blocked for1 h in 5% non-fat dry milk in TBST (20 mM Tris–HCl, pH 7.4, 150 mM NaCl, 0.05% Tween 20) and incubated overnight with primary antibody diluted in 2% BSA TBST. For the detection of dADA2b, polyclonal antibodies raised in rabbits against a dADA2b-specific peptide: PAQSQRPRLIDHTGDDDA, aa 128–146 [1], for the detection of histone H3 (Abcam ab1791) and K9-acetylated H3 polyclonal antibodies (Abcam ab4441) were used. To monitor loading, actin was detected by anti-actin polyclonal (Sigma A5060) antibody. Membranes were washed, incubated with horseradish peroxidase-conjugated anti-rabbit secondary antibodies (DAKO), washed again extensively, and developed using the ECL (Millipore) kit following the manufacturer’s recommendations.

### Co-immunoprecipitations

*Drosophila* Schneider S2 cells were transiently transfected with plasmid constructs as indicated using Effectene (Qiagen), according to the recommendations of the manufacturer. Cells were harvested by centrifugation, washed with 1 ml of ice cold PBS and lysed with 0.5 ml of RIPA buffer [10 mM Tris–HCl (pH 7.5), 150 mM NaCl, 1 mM EDTA, 1% NP-40, 0.5% SDS, 0.1 mM PMSF, 0.1 mM aprotinin and 0.1 mM leupeptin] for 30 min on ice. Clarified cell lysates were then incubated overnight with 20 μl M2-agarose beads (Sigma). The immunoprecipitates were washed five times with TBST buffer [50 mM Tris–HCl (pH 7.5), 400 mM NaCl, 1% Tween 20]. Proteins were eluted from the beads in sodium dodecyl sulfate loading buffer, resolved by SDS-PAGE and detected by Western blotting using the following antibodies: Anti-HA tag antibody (Abcam ab9110) in 1:5000 dilution, monoclonal Anti-FLAG M2 antibody (Sigma) in 1:10000 dilution.

### Immunostaining

For immunostaining of polytene chromosome spreads obtained from salivary glands of wandering larvae were treated in phosphate-buffered saline (PBS) containing 3.7% formaldehyde and 45% acetic acid. Slides were blocked in PBST (PBS + 0.1% Tween20) supplemented with 5% fetal calf serum for 1 h at 25°C and incubated overnight at 4°C in mixture of anti-histone H3K9ac (Abcam ab4441, 1:200) or anti-histone H3K14ac polyclonal (Upstate 06–911, 1:200) and anti-RNA polymerase II large subunit monoclonal [21] (7G5, 1:500) antibodies. Samples were washed in PBST and incubated with a mixture of secondary antibodies (Alexa Fluor 555-conjugated anti-rabbit-, and Alexa Fluor 488-conjugated anti-mouse IgGs, Molecular Probes) for 1 h at 25°C. The slides were washed again, covered with VectaShield mounting medium containing DAPI and examined with an OLYMPUS BX51 microscope. Photos were taken with an Olympus DP70 camera using identical settings for mutant and control samples.

### Microarray analysis

Total RNA was isolated from groups of 10 larvae using RNeasy Mini Kit (Qiagen). Control and mutant animals were synchronized at spiracle eversion and collected for RNA preparation. RNA labeling, hybridization to Affymetrix GeneChips Drosophila Genome 2.0 Array and scanning was performed at the IGBMC DNA CHIP Facility following the recommended standard Affymetrix protocols. The microarray hybridizations were performed using three biological replicates for each genotype studied. Only those genes which were indicated as “present” in at least two out of three samples of a given genotype were included in data analysis. The genes were filtered to show at least 2-fold up- or down-regulation for activation or repression categories, respectively by their log_2_ expression change value and also by their p value.

For the microarray experiments the following genotypes were used:

Control/wild type - *w*^*1118*^*/w*^*1118*^*; +/+; +/+*

dADA2bL - *w/w; +/+; P{dAda2bL-EGFP}dAda2b*^*d842*^*/P{dAda2bL-EGFP}dAda2b*^*d842*^

dADA2bS - *w/w; P{dAda2bS-EGFP}/P{dAda2bS-EGFP}; dAda2b*^*d842*^*/dAda2b*^*d842*^

The gene ontology categories were scored at the NetAffX website according the Affymetrix sample ID number. More details can be found at the Affymetrix supporting webpage.

[http://www.affymetrix.com/support/help/IVT_glossary/index.affx]

### Availability of supporting data

Microarray data sets for the different genotypes are available at ebi the microarray database [http://www.ebi.ac.uk/arrayexpress/browse.html] referred to as E-MEXP-2125 (*w*^*1118*^ and *dAda2b*^*842*^) [13] and E-MEXP-3747 (ADA2bS and ADA2bL).

## Abbreviations

ADA: Alteration/deficiency in activation;HAT: Histone acetyltransferase;SAGA: Spt-Ada-Gcn5-acetyltransferase;GCN5/KAT2: General control non-derepressible 5/lysine acetyltransferase 2;ATAC: Ada2A-containing

## Competing interest

The authors declare no competing interest.

## Authors’ contributions

TP construction of transgenes, microarray analysis, manuscript preparation; NZS rescue experiments, phenotype characterization of transgene-carriers, manuscript preparation; EV protein interaction experiments; OK establishment of transgene-carriers, genetic crosses; L.B. experimental plan, genetic analysis; IB experimental plan, manuscript preparation. All authors read and approved the final manuscript.

## Supplementary Material

Additional file 1: Figure S1 Expression of dADA2bS isoform in transgene-carrier *dAda2b* larvae. Protein extracts prepared from equal numbers of late third instar larvae of the indicated genotypes were analysed on western blot developed with dADA2b specific antibody. The blot shows the high level expression of native dADA2bS isoform under the control of *da-GAL4* driver in *UAS-dAda2bS* transgene-carrier animals. Click here for file

Additional file 2: Table S1 List of selected genes which display at least twofold expression change in *dAda2b* null and/or *dAda2bS-EGFP* transgene-carrier *dAda2b* (enhanced by gray background) mutants. mRNA levels are compared to that of wild type (w^*1118*^). Averages of three biological replicas, corresponding p values and gene ontology (GO) categories are shown. The gene ontology categories were scored by NetAffX website according the Affymetrix sample ID number. Click here for file

## References

[B1] Muratoglu S Georgieva S Papai G Scheer E Enunlu I Komonyi O Cserpan I Lebedeva L Nabirochkina E Udvardy A Two different Drosophila ADA2 homologues are present in distinct GCN5 histone acetyltransferase-containing complexes Mol Cell Biol 2003 23 1 306 321 10.1128/MCB.23.1.306-321.2003 12482983 PMC140672

[B2] Kusch T Guelman S Abmayr SM Workman JL Two Drosophila Ada2 homologues function in different multiprotein complexes Mol Cell Biol 2003 23 9 3305 3319 10.1128/MCB.23.9.3305-3319.2003 12697829 PMC153191

[B3] Grant PA Eberharter A John S Cook RG Turner BM Workman JL Expanded lysine acetylation specificity of Gcn5 in native complexes J Biol Chem 1999 274 9 5895 5900 10.1074/jbc.274.9.5895 10026213

[B4] Balasubramanian R Pray-Grant MG Selleck W Grant PA Tan S Role of the Ada2 and Ada3 transcriptional coactivators in histone acetylation J Biol Chem 2002 277 10 7989 7995 10.1074/jbc.M110849200 11773077

[B5] Candau R Berger SL Structural and functional analysis of yeast putative adaptors. Evidence for an adaptor complex *in vivo* J Biol Chem 1996 271 9 5237 5245 10.1074/jbc.271.9.5237 8617808

[B6] Stockinger EJ Mao Y Regier MK Triezenberg SJ Thomashow MF Transcriptional adaptor and histone acetyltransferase proteins in Arabidopsis and their interactions with CBF1, a transcriptional activator involved in cold-regulated gene expression Nucleic Acids Res 2001 29 7 1524 1533 10.1093/nar/29.7.1524 11266554 PMC31267

[B7] Guelman S Suganuma T Florens L Swanson SK Kiesecker CL Kusch T Anderson S Yates JR 3rd Washburn MP Abmayr SM Host cell factor and an uncharacterized SANT domain protein are stable components of ATAC, a novel dAda2A/dGcn5-containing histone acetyltransferase complex in Drosophila Mol Cell Biol 2006 26 3 871 882 10.1128/MCB.26.3.871-882.2006 16428443 PMC1347012

[B8] Nagy Z Tora L Distinct GCN5/PCAF-containing complexes function as co-activators and are involved in transcription factor and global histone acetylation Oncogene 2007 26 37 5341 5357 10.1038/sj.onc.1210604 17694077

[B9] Spedale G Timmers HT Pijnappel WW ATAC-king the complexity of SAGA during evolution Genes Dev 2012 26 6 527 541 10.1101/gad.184705.111 22426530 PMC3315114

[B10] Gamper AM Kim J Roeder RG The STAGA subunit ADA2b is an important regulator of human GCN5 catalysis Mol Cell Biol 2009 29 1 266 280 10.1128/MCB.00315-08 18936164 PMC2612497

[B11] Qi D Larsson J Mannervik M Drosophila Ada2b is required for viability and normal histone H3 acetylation Mol Cell Biol 2004 24 18 8080 8089 10.1128/MCB.24.18.8080-8089.2004 15340070 PMC515027

[B12] Pankotai T Komonyi O Bodai L Ujfaludi Z Muratoglu S Ciurciu A Tora L Szabad J Boros I The homologous Drosophila transcriptional adaptors ADA2a and ADA2b are both required for normal development but have different functions Mol Cell Biol 2005 25 18 8215 8227 10.1128/MCB.25.18.8215-8227.2005 16135810 PMC1234310

[B13] Zsindely N Pankotai T Ujfaludi Z Lakatos D Komonyi O Bodai L Tora L Boros IM The loss of histone H3 lysine 9 acetylation due to dSAGA-specific dAda2b mutation influences the expression of only a small subset of genes Nucleic Acids Res 2009 37 20 6665 6680 10.1093/nar/gkp722 19740772 PMC2777428

[B14] Sterner DE Wang X Bloom MH Simon GM Berger SL The SANT domain of Ada2 is required for normal acetylation of histones by the yeast SAGA complex J Biol Chem 2002 277 10 8178 8186 10.1074/jbc.M108601200 11777910

[B15] Weake VM Lee KK Guelman S Lin CH Seidel C Abmayr SM Workman JL SAGA-mediated H2B deubiquitination controls the development of neuronal connectivity in the Drosophila visual system EMBO J 2008 27 2 394 405 10.1038/sj.emboj.7601966 18188155 PMC2234343

[B16] Vamos E Boros IM The C-terminal domains of ADA2 proteins determine selective incorporation into GCN5-containing complexes that target histone H3 or H4 for acetylation FEBS Lett 2012 586 19 3279 3286 10.1016/j.febslet.2012.06.051 22796493

[B17] Koutelou E Hirsch CL Dent SY Multiple faces of the SAGA complex Curr Opin Cell Biol 2010 22 3 374 382 10.1016/j.ceb.2010.03.005 20363118 PMC2900470

[B18] Guelman S Suganuma T Florens L Weake V Swanson SK Washburn MP Abmayr SM Workman JL The essential gene wda encodes a WD40 repeat subunit of Drosophila SAGA required for histone H3 acetylation Mol Cell Biol 2006 26 19 7178 7189 10.1128/MCB.00130-06 16980620 PMC1592886

[B19] Daniel JA Grant PA Multi-tasking on chromatin with the SAGA coactivator complexes Mutat Res 2007 618 1–2 135 148 17337012 10.1016/j.mrfmmm.2006.09.008 PMC1892243

[B20] Ryder E Blows F Ashburner M Bautista-Llacer R Coulson D Drummond J Webster J Gubb D Gunton N Johnson G The DrosDel collection: a set of P-element insertions for generating custom chromosomal aberrations in Drosophila melanogaster Genetics 2004 167 2 797 813 10.1534/genetics.104.026658 15238529 PMC1470913

[B21] Besse S Vigneron M Pichard E Puvion-Dutilleul F Synthesis and maturation of viral transcripts in herpes simplex virus type 1 infected HeLa cells: the role of interchromatin granules Gene Expr 1995 4 3 143 161 7734948 PMC6134381

