# Supplementary figures and images for: Functional characterization and gene expression profiling of Drosophila melanogaster short dADA2b isoform-containing dSAGA complexes
Source: BMC Genomics. 2013 Jan 22;14:44. doi: 10.1186/1471-2164-14-44 (PMC3598691; doi:10.1186/1471-2164-14-44)

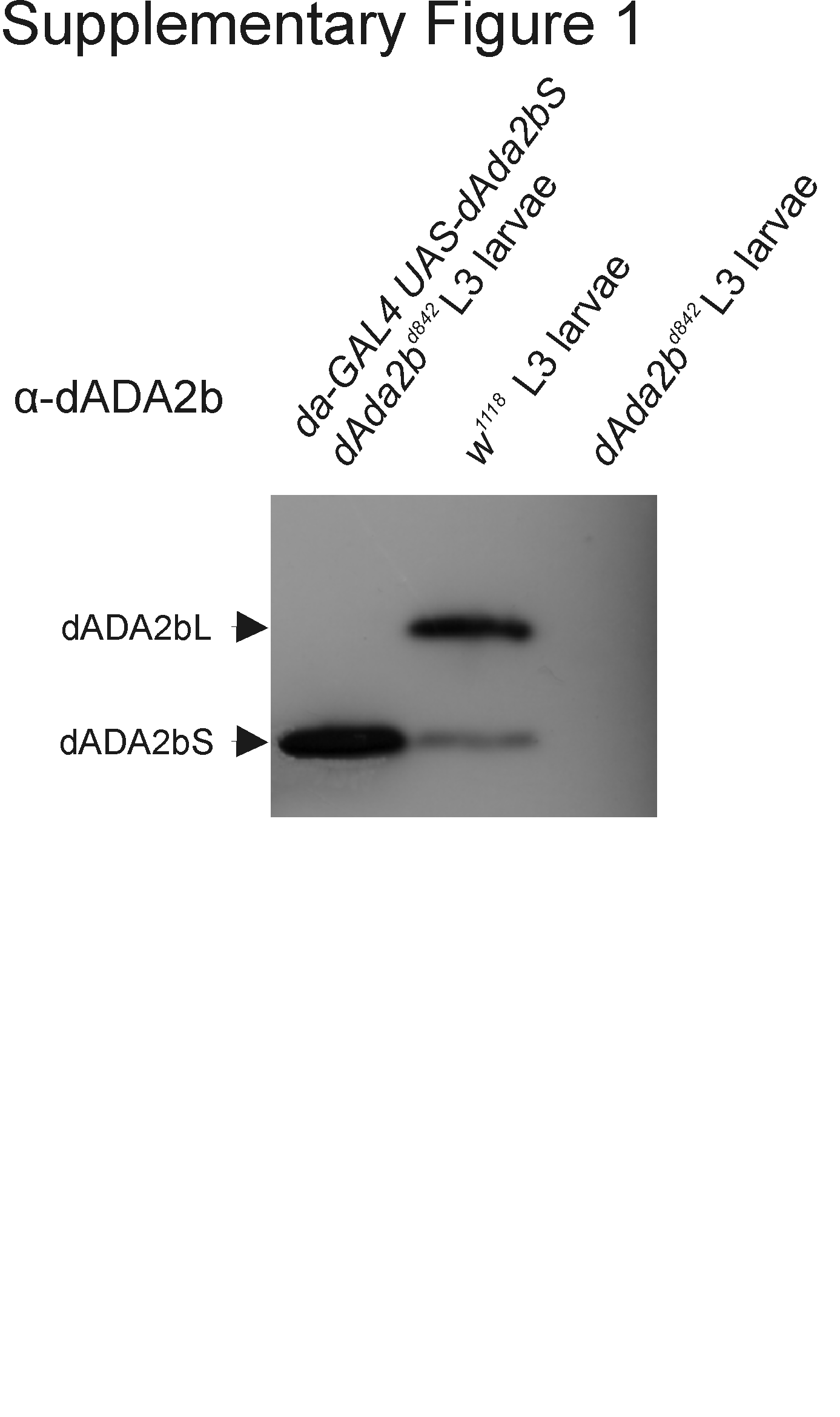

Supplement: Additional file 1: Figure S1 — Expression of dADA2bS isoform in transgene-carrier dAda2b larvae. Protein extracts prepared from equal numbers of late third instar larvae of the indicated genotypes were analysed on western blot developed with dADA2b specific antibody. The blot shows the high level expression of native dADA2bS isoform under the control of da-GAL4 driver in UAS-dAda2bS transgene-carrier animals. [file 1471-2164-14-44-S1.tiff]
