# Supplementary material for: Functional characterization and gene expression profiling of Drosophila melanogaster short dADA2b isoform-containing dSAGA complexes
Source: BMC Genomics. 2013 Jan 22;14:44. doi: 10.1186/1471-2164-14-44 (PMC3598691; doi:10.1186/1471-2164-14-44)
Supplement: Additional file 2: Table S1 — List of selected genes which display at least twofold expression change in dAda2b null and/or dAda2bS-EGFP transgene-carrier dAda2b (enhanced by gray background) mutants. mRNA levels are compared to that of wild type (w1118). Averages of three biological replicas, corresponding p values and gene ontology (GO) categories are shown. The gene ontology categories were scored by NetAffX website according the Affymetrix sample ID number. [file 1471-2164-14-44-S2.pdf]

Activation

Gene Title	w1118 average of 3 replicates	dAda2b average of 3 replictes	Ada2bS average of 3 replicates	log2(dAda2b/w1118)	log2(dAda2bS/w1118)	log2(dAda2bS/Ada2b)	p value w1118 vs Ada2bS	p value Ada2b vs Ada2bS	go biological process term	go molecular function term
CG32352	8,9	18,1	18,1	1,02	1,02	0,00	2,93E-02	4,98E-01	---	---
CG13921	58,7	176,7	168,2	1,59	1,52	-0,07	1,79E-02	3,60E-01	---	---
CG4250	150,1	1148,0	569,3	2,93	1,92	-1,01	5,47E-02	7,60E-03	---	---
CG31633	43,6	70,0	90,0	0,68	1,05	0,36	5,73E-04	6,41E-03	---	---
CG5326	13,8	19,5	20,4	0,49	0,56	0,07	2,12E-02	4,04E-01	---	---
CG31410	750,6	2606,3	2898,9	1,80	1,95	0,15	4,62E-03	2,31E-01	---	---
CG2127	258,6	326,2	565,0	0,33	1,13	0,79	4,17E-02	2,09E-01	---	---
CG15705	17,9	37,5	37,3	1,07	1,06	-0,01	8,13E-02	4,96E-01	---	---
CG17217	10,8	25,9	19,6	1,26	0,86	-0,40	1,41E-02	2,66E-01	---	---
CG10801	10,3	19,2	18,7	0,89	0,85	-0,04	8,28E-02	4,79E-01	---	---
CG15287 /// CG33090	28,0	36,2	57,9	0,37	1,05	0,68	1,20E-02	1,99E-01	---	---
CG9322	40,1	92,7	115,2	1,21	1,52	0,31	4,21E-03	5,22E-02	---	---
CG12729	8,2	19,5	15,7	1,24	0,93	-0,32	1,09E-01	3,47E-01	---	---
CG3557	41,2	63,3	91,5	0,62	1,15	0,53	8,61E-02	2,96E-01	---	---
CG31391	47,0	71,6	77,4	0,61	0,72	0,11	1,27E-01	4,46E-01	---	---
CG12035	21,4	32,3	39,2	0,60	0,87	0,28	1,07E-01	3,98E-01	---	---
CG14949	55,3	123,7	128,2	1,16	1,21	0,05	3,64E-02	4,27E-01	---	---
CG9822	264,8	761,4	773,5	1,52	1,55	0,02	2,37E-03	3,94E-01	---	---
CG7530	167,3	270,1	363,5	0,69	1,12	0,43	5,85E-03	1,95E-02	---	---
CG2233	2046,8	4544,6	5657,1	1,15	1,47	0,32	4,20E-03	1,83E-03	---	---
Antigen 5-related 2	134,7	780,0	314,2	2,53	1,22	-1,31	7,80E-03	3,69E-02	---	---
CG11298	19,4	29,1	31,3	0,58	0,69	0,11	3,65E-02	4,53E-01	---	---
CG15506	40,8	114,8	140,5	1,49	1,78	0,29	1,18E-03	2,97E-02	---	---
CG40115	1,7	3,5	4,7	1,03	1,47	0,44	3,59E-02	3,15E-01	---	---
CG31676	55,6	110,8	143,3	0,99	1,37	0,37	3,13E-03	2,05E-02	---	---
CG3588	58,2	85,2	122,1	0,55	1,07	0,52	4,04E-03	7,30E-02	---	---
CG11854	119,4	313,7	271,3	1,39	1,18	-0,21	4,45E-03	7,32E-02	---	---
CG16885	331,1	422,2	695,2	0,35	1,07	0,72	1,20E-02	1,46E-02	---	---
proximal to raf	44,6	97,5	83,7	1,13	0,91	-0,22	3,41E-03	3,53E-03	---	---
CG1079	11,2	20,8	20,9	0,90	0,90	0,01	7,62E-05	4,92E-01	---	---
CG33333	140,2	537,1	631,5	1,94	2,17	0,23	6,50E-03	1,31E-01	---	---
CG31467	26,6	41,2	46,4	0,63	0,80	0,17	8,55E-02	3,90E-01	---	---
CG18746	7,1	19,8	14,2	1,48	1,00	-0,47	8,80E-02	2,21E-01	---	---
CG2750	20,2	25,7	45,7	0,35	1,18	0,83	1,76E-02	1,73E-01	---	---
CG5767	262,8	539,0	567,0	1,04	1,11	0,07	1,10E-02	4,20E-01	---	---
CG31533	17,3	27,1	44,2	0,65	1,35	0,70	1,86E-02	1,72E-01	---	---
CG11349	45,3	45,8	95,9	0,01	1,08	1,07	1,15E-02	1,55E-02	---	---
CG31672	13,0	47,2	35,7	1,86	1,46	-0,40	1,44E-02	1,17E-01	---	---
CG12057	998,7	3051,9	2468,8	1,61	1,31	-0,31	1,22E-02	2,00E-02	---	---
CG7272	85,4	245,8	232,2	1,52	1,44	-0,08	1,23E-03	6,60E-02	---	---
CG13924	22,2	245,5	113,5	3,47	2,36	-1,11	1,10E-03	7,17E-03	---	---
Sdic:CG32823	34,6	43,6	94,4	0,33	1,45	1,11	6,02E-02	1,47E-01	---	---
CG30148	30,4	103,1	102,5	1,76	1,76	-0,01	6,60E-03	4,95E-01	---	---
CG1468	158,7	239,3	382,4	0,59	1,27	0,68	1,21E-02	5,63E-02	---	---
CG31126	109,1	375,5	339,5	1,78	1,64	-0,15	1,58E-03	6,87E-02	---	---
CG13905	224,2	936,5	561,0	2,06	1,32	-0,74	2,14E-02	1,80E-02	---	---
CG8138	48,2	71,9	78,5	0,58	0,70	0,13	9,07E-02	4,26E-01	---	---
CG31698	116,8	218,3	287,7	0,90	1,30	0,40	2,76E-02	2,76E-02	---	---
CG13314	99,3	463,4	394,8	2,22	1,99	-0,23	5,15E-03	1,63E-01	---	---
CG33140	14,6	23,3	21,2	0,68	0,54	-0,14	9,04E-02	4,38E-01	---	---
CG14000	7,0	15,5	19,9	1,14	1,50	0,36	2,26E-04	3,04E-01	---	---
CG14926	159,0	359,8	522,8	1,18	1,72	0,55	2,58E-02	2,66E-01	---	---
CG7669	26,4	40,5	57,1	0,62	1,12	0,50	8,64E-02	3,03E-01	---	---
CG32023	2,6	76,0	159,8	4,87	5,94	1,07	3,02E-04	1,21E-02	---	---
CG11584	8,7	30,0	25,8	1,79	1,57	-0,22	6,28E-03	1,25E-01	---	---
CG14839	6,3	16,9	19,8	1,42	1,64	0,22	5,15E-02	4,03E-01	---	---
CG15404	124,8	281,3	661,5	1,17	2,41	1,23	3,67E-02	5,39E-02	---	---
CG5506	270,3	1244,2	771,2	2,20	1,51	-0,69	8,29E-03	4,49E-02	---	---
CG7587	351,1	2919,5	4467,1	3,06	3,67	0,61	2,50E-03	6,06E-02	---	---
CG5676	209,2	316,0	435,5	0,60	1,06	0,46	1,25E-02	4,22E-03	---	---
CG9877	76,9	229,2	366,6	1,58	2,25	0,68	5,22E-03	1,72E-02	---	---
CG5391	433,7	1503,5	1468,9	1,79	1,76	-0,03	3,76E-03	3,81E-01	---	---
CG15909	11,7	18,7	15,0	0,68	0,36	-0,32	2,36E-01	3,95E-01	---	---
CG4712	47,1	62,4	102,6	0,41	1,12	0,72	5,18E-02	1,75E-01	---	---
CG31948	154,7	301,2	371,1	0,96	1,26	0,30	5,91E-02	3,50E-01	---	---
CG13460	27,1	121,4	85,5	2,16	1,66	-0,51	1,19E-01	2,89E-01	---	---
CG13405	20,9	33,8	44,7	0,69	1,09	0,40	6,24E-02	3,16E-01	---	---
CG13661	19,9	31,7	43,1	0,68	1,12	0,44	4,01E-02	2,53E-01	---	---
CG15784	142,0	610,0	458,2	2,10	1,69	-0,41	2,90E-02	2,43E-01	---	---
CG10734	139,3	231,4	307,2	0,73	1,14	0,41	1,88E-02	2,65E-01	---	---
CG33278	19,9	35,4	48,5	0,83	1,29	0,45	4,83E-02	3,14E-01	---	---
CG14302	448,8	1037,2	1145,5	1,21	1,35	0,14	1,71E-02	1,79E-01	---	---
CG15870	24,8	63,2	60,7	1,35	1,29	-0,06	1,93E-02	3,51E-01	---	---
CG10289	62,7	85,5	133,9	0,45	1,09	0,65	6,66E-02	2,14E-03	---	---
CG15155	52,1	167,0	113,4	1,68	1,12	-0,56	8,35E-02	2,38E-01	---	acyltransferase activity /// transferase activity
CG30471	40,5	87,5	193,6	1,11	2,26	1,14	8,50E-03	9,09E-03	---	acyltransferase activity /// transferase activity /// transferase activity, transferring acyl groups other than amino-acyl groups
CG31694	429,1	906,7	868,1	1,08	1,02	-0,06	3,60E-03	2,48E-02	---	binding
CG6614	10,0	15,7	25,1	0,66	1,33	0,68	3,85E-02	1,81E-01	---	binding
CG6073	13,1	24,6	26,6	0,90	1,02	0,12	5,77E-02	3,29E-01	---	binding /// protein binding
CG5155	38,2	58,6	68,1	0,62	0,83	0,22	2,88E-02	4,01E-01	---	binding /// protein binding
CG3244	443,2	906,9	971,2	1,03	1,13	0,10	1,39E-03	3,62E-01	---	binding /// sugar binding
CG7526	12,2	28,0	28,9	1,20	1,24	0,05	6,18E-03	6,06E-03	---	calcium ion binding
CG33490	25,3	41,4	48,8	0,71	0,95	0,24	1,96E-02	3,70E-01	---	calcium ion binding
CG10841	31,7	33,6	76,4	0,08	1,27	1,18	2,65E-02	1,18E-01	---	calcium ion binding
CG4704	8,7	19,0	17,6	1,12	1,02	-0,11	1,09E-01	4,37E-01	---	calcium ion binding /// protein binding
Cystatin-like	722,5	2109,2	2217,6	1,55	1,62	0,07	1,33E-03	2,24E-01	---	cysteine-type endopeptidase inhibitor activity /// cysteine-type endopeptidase inhibitor activity /// cysteine-type endopeptidase inhibitor activity /// peptidase inhibitor activity
CG6461	158,6	285,5	320,6	0,85	1,02	0,17	1,02E-03	1,00E-01	---	gamma-glutamyltransferase activity /// gamma-glutamyltransferase activity /// acyltransferase activity /// transferase activity
miple2	476,9	946,0	1003,0	0,99	1,07	0,08	1,34E-03	2,36E-01	---	growth factor activity /// heparin binding
CG15429	12,5	22,6	24,3	0,85	0,96	0,11	1,95E-02	4,25E-01	---	heme binding
CG3982	228,9	270,2	475,9	0,24	1,06	0,82	5,49E-02	1,94E-01	---	hydrolase activity
CG12289	6,8	14,5	16,9	1,10	1,31	0,22	5,14E-02	4,19E-01	---	ketohexokinase activity /// ketohexokinase activity /// protein binding /// transferase activity
CG7551	6,9	18,3	28,7	1,41	2,06	0,65	4,99E-02	1,81E-01	---	ketohexokinase activity /// ketohexokinase activity /// transferase activity
CG6429	12,5	42,8	55,2	1,77	2,14	0,37	1,38E-02	1,92E-01	---	lysozyme activity /// protein binding
CG31735	60,9	82,4	125,1	0,44	1,04	0,60	5,65E-02	1,93E-01	---	myosin light chain binding
CG33346	663,6	2190,2	1916,5	1,72	1,53	-0,19	8,98E-03	1,00E-01	---	nucleic acid binding /// protein binding /// hydrolase activity /// metal ion binding
CG17386	105,7	203,2	212,7	0,94	1,01	0,07	2,07E-02	4,20E-01	---	nucleotide binding
CG32364	8,4	29,7	19,9	1,82	1,24	-0,58	4,16E-03	1,20E-01	---	nucleotide binding /// nucleic acid binding
CG10513	27,9	187,0	106,3	2,74	1,93	-0,82	1,48E-02	8,99E-03	---	protein binding
CG8620	19,2	84,6	149,1	2,14	2,96	0,82	1,71E-03	1,18E-02	---	protein binding
Mst33A	115,9	168,6	239,3	0,54	1,05	0,50	5,78E-02	2,72E-01	---	protein binding
CG14332	305,3	909,5	1414,6	1,58	2,21	0,64	2,11E-03	5,41E-02	---	protein binding
CG1138	24,0	44,6	43,5	0,90	0,86	-0,04	6,51E-02	4,80E-01	---	protein binding
Prosap	131,3	263,4	301,1	1,00	1,20	0,19	1,97E-02	2,09E-01	---	protein binding
CG7886	66,7	86,0	135,4	0,37	1,02	0,65	3,40E-02	2,47E-01	---	protein binding

CG11523	98,4	188,8	198,3	0,94	1,01	0,07	1,34E-03	3,85E-01	---	protein binding
CG12115	460,4	2233,3	1461,1	2,28	1,67	-0,61	1,64E-02	1,79E-02	---	protein binding
CG7251	50,5	64,4	104,6	0,35	1,05	0,70	4,56E-02	1,70E-01	---	protein binding
CG13067	75,4	292,4	249,3	1,95	1,72	-0,23	7,61E-03	2,25E-01	---	protein binding
CG1314	25,6	25,5	55,2	-0,01	1,11	1,12	4,20E-02	9,87E-02	---	protein binding
CG13245	145,6	218,0	231,5	0,58	0,67	0,09	1,02E-01	4,61E-01	---	protein binding
CG10307	17,3	24,3	34,9	0,49	1,02	0,52	4,92E-02	2,64E-01	---	protein binding
CG14383	13,7	20,8	23,4	0,61	0,78	0,17	2,01E-01	4,33E-01	---	protein binding
CG10510	24,6	39,3	64,1	0,68	1,38	0,71	1,07E-02	1,54E-01	---	protein binding
CG3610	32,0	48,7	72,6	0,61	1,18	0,58	4,54E-02	2,18E-01	---	protein binding
CG2871	37,6	58,1	71,1	0,63	0,92	0,29	3,00E-02	3,43E-01	---	protein binding
Male-specific RNA 98Ca	346,8	449,5	701,5	0,37	1,02	0,64	5,47E-02	2,32E-01	---	protein binding
CG31820	274,7	408,5	369,9	0,57	0,43	-0,14	1,87E-01	4,39E-01	---	protein binding
CG18266	53,3	73,3	107,4	0,46	1,01	0,55	5,44E-02	2,58E-01	---	protein binding
CG2217	23,9	49,4	118,7	1,05	2,31	1,27	1,66E-01	2,09E-01	---	protein binding
CG8292	21,4	43,0	53,8	1,00	1,33	0,32	5,01E-03	3,33E-01	---	protein binding
CG13482	572,3	1811,9	1527,7	1,66	1,42	-0,25	1,79E-03	4,62E-02	---	protein binding
CG31029	56,7	85,4	126,9	0,59	1,16	0,57	4,44E-02	2,73E-01	---	protein binding
CG31244	27,4	44,0	52,4	0,68	0,93	0,25	2,76E-02	3,69E-01	---	protein binding
CG3124	164,2	248,1	330,7	0,59	1,01	0,41	7,40E-02	3,14E-01	---	protein binding
CG14154	23,4	35,2	50,9	0,59	1,12	0,53	7,36E-02	2,79E-01	---	protein binding
CG6569	40,8	63,8	89,9	0,65	1,14	0,49	6,89E-02	2,72E-01	---	protein binding
CG9192	46,9	107,1	101,5	1,19	1,11	-0,08	1,43E-02	3,29E-01	---	protein binding
CG30161	16,3	30,5	34,9	0,90	1,09	0,19	1,35E-01	4,22E-01	---	protein binding
CG15878	23,4	39,8	54,1	0,76	1,21	0,44	1,85E-02	2,91E-01	---	protein binding
CG2082	80,1	143,0	190,2	0,84	1,25	0,41	2,04E-02	1,16E-01	---	protein binding
CG12377	12,8	17,9	27,8	0,48	1,12	0,64	6,10E-02	2,06E-01	---	protein binding
CG14864	29,9	111,0	76,4	1,89	1,35	-0,54	2,59E-02	5,61E-02	---	protein binding
CG13843	18,9	32,8	46,8	0,79	1,31	0,51	7,32E-02	3,12E-01	---	protein binding
CG13043	26,9	93,5	73,2	1,80	1,44	-0,35	1,06E-03	8,30E-02	---	protein binding
CG14183	39,7	54,6	80,2	0,46	1,01	0,55	2,15E-02	1,70E-01	---	protein binding /// ATP binding
Metallothionein A	1206,4	2254,6	2748,3	0,90	1,19	0,29	1,06E-02	1,80E-01	---	protein binding /// metal ion binding /// metal ion binding
CG11253	52,1	62,6	113,4	0,26	1,12	0,86	6,83E-02	1,85E-01	---	protein binding /// zinc ion binding /// metal ion binding
CG7966	208,0	398,3	481,9	0,94	1,21	0,27	7,55E-04	8,65E-02	---	selenium binding /// selenium binding
CG16713	163,1	238,0	588,2	0,54	1,85	1,31	2,73E-02	7,90E-02	---	serine-type endopeptidase inhibitor activity /// serine-type endopeptidase inhibitor activity
CG3604	70,6	151,6	163,9	1,10	1,22	0,11	3,84E-03	2,61E-01	---	serine-type endopeptidase inhibitor activity /// serine-type endopeptidase inhibitor activity
CG11018 /// Larval cuticle protein 4	653,1	725,1	1878,1	0,15	1,52	1,37	7,46E-02	8,02E-02	---	structural constituent of chitin-based cuticle /// structural constituent of chitin-based larval cuticle /// structural constituent of cuticle
CG13222	122,7	510,8	268,2	2,06	1,13	-0,93	6,05E-03	1,82E-02	---	structural constituent of chitin-based cuticle /// structural constituent of chitin-based larval cuticle /// structural constituent of cuticle
CG10625	681,7	1029,9	1381,0	0,60	1,02	0,42	8,59E-04	4,98E-02	---	structural constituent of cuticle
CG1941	29,2	94,7	79,9	1,70	1,45	-0,25	1,25E-02	2,45E-02	---	transferase activity, transferring acyl groups other than amino-acyl groups
CG4956	15,5	28,8	35,7	0,90	1,21	0,31	3,29E-02	3,22E-01	---	zinc ion binding /// metal ion binding
Attacin-A	76,7	156,4	285,1	1,03	1,89	0,87	1,29E-02	1,22E-01	antibacterial humoral response /// antibacterial humoral response /// defense response to bacterium /// defense response to bacterium /// innate immune response /// defense response to Gram-negative bacterium /// defense response to Gram-negative bacterium	---
CG7860	564,3	1253,9	1151,8	1,15	1,03	-0,12	2,29E-03	2,29E-01	asparagine catabolic process via L-aspartate /// salivary gland cell autophagic cell death /// autophagic cell death	N4-(beta-N-acetylglucosaminyl)-L-asparaginase activity /// asparaginase activity /// asparaginase activity /// protein binding /// hydrolase activity
CG11703	16,3	25,5	27,6	0,64	0,76	0,11	1,22E-01	4,48E-01	ATP biosynthetic process /// potassium ion transport /// sodium ion transport	sodium:potassium-exchanging ATPase activity /// sodium:potassium-exchanging ATPase activity
CG17923	14,9	27,1	26,6	0,86	0,83	-0,03	1,31E-01	4,88E-01	ATP biosynthetic process /// transport /// ion transport /// cation transport /// metabolic process /// monovalent inorganic cation transport	nucleotide binding /// catalytic activity /// sodium:potassium-exchanging ATPase activity /// sodium:potassium-exchanging ATPase activity /// ATP binding /// monovalent inorganic cation transmembrane transporter activity /// ATPase activity, coupled to transmembrane movement of ions, phosphorylative mechanism /// hydrolase activity /// hydrolase activity, acting on acid anhydrides, catalyzing transmembrane movement of substances
CTP:phosphocholine cytidylyltransferase 2	35,5	54,3	80,9	0,61	1,19	0,58	4,01E-02	2,13E-01	biosynthetic process	catalytic activity /// choline-phosphate cytidylyltransferase activity /// choline-phosphate cytidylyltransferase activity /// choline-phosphate cytidylyltransferase activity /// transferase activity /// nucleotidyltransferase activity
CG31773	24,1	32,9	57,1	0,45	1,24	0,80	7,11E-02	1,77E-01	biosynthetic process /// folic acid and derivative biosynthetic process /// folic acid and derivative biosynthetic process	tetrahydrofolylpolyglutamate synthase activity /// tetrahydrofolylpolyglutamate synthase activity /// ATP binding /// ligase activity
CG15918	25,4	55,0	56,7	1,11	1,16	0,04	1,26E-02	4,04E-01	carbohydrate metabolic process	catalytic activity /// hydrolase activity /// hydrolase activity, acting on carbon-nitrogen (but not peptide) bonds
Imaginal disc growth factor 3	22,6	40,2	55,2	0,83	1,29	0,46	2,32E-02	1,58E-01	carbohydrate metabolic process /// chitin catabolic process /// multicellular organismal development /// imaginal disc development	catalytic activity /// hydrolase activity, hydrolyzing O-glycosyl compounds /// chitinase activity /// chitinase activity /// imaginal disc growth factor receptor binding /// cation binding
CG31169	44,3	48,7	92,3	0,13	1,06	0,92	6,20E-02	1,48E-01	carbohydrate metabolic process /// glycerol-3-phosphate metabolic process /// glycerol-3-phosphate metabolic process /// metabolic process /// glycerol-3-phosphate catabolic process /// oxidation reduction	DNA binding /// catalytic activity /// glycerol-3-phosphate dehydrogenase [NAD+] activity /// glycerol-3-phosphate dehydrogenase [NAD+] activity /// glycerol-3-phosphate dehydrogenase activity /// binding /// oxidoreductase activity /// oxidoreductase activity, acting on CH-OH group of donors /// oxidoreductase activity, acting on the CH-OH group of donors, NAD or NADP as acceptor /// coenzyme binding /// NAD or NADH binding
CG5322	134,0	253,7	347,2	0,92	1,37	0,45	4,16E-03	2,57E-02	carbohydrate metabolic process /// mannose metabolic process /// metabolic process	catalytic activity /// hydrolase activity, hydrolyzing O-glycosyl compounds /// alpha-mannosidase activity /// alpha-mannosidase activity /// zinc ion binding /// mannosidase activity /// hydrolase activity /// hydrolase activity, acting on glycosyl bonds /// carbohydrate binding /// cation binding
CG18473	94,9	189,7	144,1	1,00	0,60	-0,40	2,26E-03	1,31E-01	catabolic process	aryldialkylphosphatase activity /// zinc ion binding /// hydrolase activity /// hydrolase activity /// hydrolase activity, acting on ester bonds /// metal ion binding
Muscle protein 20	642,1	804,1	1290,3	0,32	1,01	0,68	1,32E-02	1,76E-02	cell adhesion /// myoblast fusion /// regulation of cell shape	actin binding /// calcium ion binding /// protein binding
scribbled	42,6	36,2	34,4	-0,24	-0,31	-0,07	1,93E-01	4,25E-01	cell morphogenesis /// establishment of imaginal disc-derived wing hair orientation /// morphogenesis of a polarized epithelium /// positive regulation of antibacterial peptide biosynthetic process /// multicellular organismal development /// pole cell migration /// dorsal closure /// dorsal closure /// nervous system development /// R3/R4 cell fate commitment /// wing disc morphogenesis /// sensory perception of smell /// asymmetric protein localization /// asymmetric protein localization /// cell proliferation /// negative regulation of cell proliferation /// gonad development /// morphogenesis of embryonic epithelium /// establishment or maintenance of polarity of embryonic epithelium /// morphogenesis of follicular epithelium /// establishment or maintenance of polarity of follicular epithelium /// morphogenesis of larval imaginal disc epithelium /// establishment or maintenance of polarity of larval imaginal disc epithelium /// septate junction assembly /// cell differentiation /// establishment or maintenance of apical/basal cell polarity /// olfactory behavior /// establishment of ommatidial planar polarity /// response to chemical stimulus /// zonula adherens assembly /// zonula adherens assembly /// establishment or maintenance of epithelial cell apical/basal polarity /// establishment or maintenance of epithelial cell apical/basal polarity /// negative regulation of imaginal disc growth /// positive regulation of heart contraction /// compound eye development /// negative regulation of epithelial cell proliferation /// regulation of synapse structure and activity /// response to stimulus /// regulation of cell cycle	hormone activity /// protein binding /// protein binding /// protein binding
CG6726	180,8	450,9	382,2	1,32	1,08	-0,24	1,72E-02	6,44E-02	cellular amino acid metabolic process /// metabolic process	aminoacylase activity /// aminoacylase activity /// protein binding /// protein binding /// hydrolase activity
CG14608	3,9	8,1	8,1	1,06	1,07	0,01	9,12E-02	4,94E-01	chitin metabolic process	chitin binding
CG32656	191,1	837,1	791,5	2,13	2,05	-0,08	5,72E-03	3,83E-01	chitin metabolic process	chitin binding
CG14959	21,0	30,6	50,9	0,54	1,28	0,73	7,97E-03	7,34E-02	chitin metabolic process	chitin binding
CG7874	348,6	859,2	796,7	1,30	1,19	-0,11	2,62E-05	2,91E-01	chitin metabolic process	chitin binding
CG31439	31,0	121,4	66,5	1,97	1,10	-0,87	1,05E-01	1,82E-01	chitin metabolic process	chitin binding /// hydrolase activity
CG3348	112,2	186,3	162,3	0,73	0,53	-0,20	2,25E-01	4,18E-01	chitin metabolic process	protein binding /// chitin binding
CG14957	953,0	2248,1	2562,6	1,24	1,43	0,19	1,80E-03	6,32E-02	chitin metabolic process	protein binding /// chitin binding
CG7290	378,9	1560,7	1375,4	2,04	1,86	-0,18	1,78E-02	2,15E-01	chitin metabolic process	protein binding /// chitin binding /// structural constituent of peritrophic membrane

scalloped	110,8	112,0	225,8	0,02	1,03	1,01	3,32E-02	7,69E-03	compound eye morphogenesis /// transcription /// regulation of transcription, DNA-dependent /// regulation of transcription from RNA polymerase II promoter /// cell cycle /// multicellular organismal development /// nervous system development /// sensory organ development /// imaginal disc-derived wing morphogenesis /// imaginal disc-derived wing morphogenesis /// imaginal disc-derived wing morphogenesis /// imaginal disc-derived leg morphogenesis /// somatic muscle development /// cell differentiation /// regulation of transcription /// cardiac muscle cell development	DNA binding /// sequence-specific DNA binding transcription factor activity /// sequence-specific DNA binding transcription factor activity /// specific RNA polymerase II transcription factor activity /// protein binding /// protein domain specific binding /// metal ion binding
Attacin-B /// Attacin-A	65,3	172,1	290,4	1,40	2,15	0,75	1,68E-02	2,00E-01	defense response /// antibacterial humoral response /// antibacterial humoral response /// antibacterial humoral response /// defense response to bacterium /// defense response to bacterium /// defense response to bacterium /// defense response to bacterium /// defense response to bacterium /// innate immune response /// defense response to Gram-negative bacterium /// defense response to Gram-negative bacterium	protein binding
drosomycin-4	26,8	135,2	95,3	2,33	1,83	-0,50	4,23E-03	5,19E-02	defense response /// defense response /// defense response /// defense response to fungus	---
PGRP-SC1a /// PGRP-SC1b	41,4	213,0	253,4	2,36	2,61	0,25	1,55E-02	2,82E-01	defense response /// immune response /// peptidoglycan catabolic process /// peptidoglycan catabolic process /// peptidoglycan catabolic process /// detection of bacterium /// innate immune response /// innate immune response /// innate immune response /// innate immune response	protein binding /// protein binding /// N-acetylmuramoyl-L-alanine amidase activity /// N-acetylmuramoyl-L-alanine amidase activity /// hydrolase activity /// peptidoglycan binding /// peptidoglycan binding /// peptidoglycan binding /// peptidoglycan binding /// metal ion binding
Toll-7	24,2	38,8	61,0	0,68	1,33	0,65	7,00E-03	4,44E-02	defense response /// signal transduction /// signal transduction /// innate immune response	transmembrane receptor activity /// transmembrane receptor activity /// transmembrane receptor activity /// protein binding
CG3810	33,1	53,9	46,5	0,70	0,49	-0,21	6,15E-02	1,10E-01	determination of adult lifespan /// ER-associated protein catabolic process	mannosyl-oligosaccharide 1,2-alpha-mannosidase activity /// mannosyl-oligosaccharide 1,2-alpha-mannosidase activity /// calcium ion binding
inturned	78,8	127,8	160,2	0,70	1,02	0,33	2,59E-02	1,93E-02	establishment of planar polarity /// establishment of imaginal disc-derived wing hair orientation /// establishment or maintenance of cell polarity /// non-sensory hair organization	protein binding
brown	19,1	35,6	36,8	0,90	0,94	0,05	5,89E-02	3,87E-01	eye pigment biosynthetic process /// ommochrome biosynthetic process /// pteridine biosynthetic process /// transport /// eye pigment precursor transport	nucleotide binding /// eye pigment precursor transporter activity /// ATP binding /// ATPase activity /// nucleoside-triphosphatase activity /// pigment binding /// ATPase activity, coupled to transmembrane movement of substances
CG10192	193,4	280,9	411,2	0,54	1,09	0,55	2,81E-03	7,62E-02	G1/S transition of mitotic cell cycle /// translational initiation /// male meiosis /// spermatogenesis /// meiotic G2/MI transition /// RNA metabolic process /// salivary gland cell autophagic cell death /// autophagic cell death /// spermatid differentiation	RNA 7-methylguanosine cap binding /// translation initiation factor activity /// binding /// protein binding
no extended memory	70,0	82,0	170,3	0,23	1,28	1,05	1,17E-03	1,86E-02	glutamine metabolic process /// memory /// locomotory behavior	glutaminase activity /// protein binding /// hydrolase activity
Glutathione Synthetase	7,9	22,9	14,5	1,54	0,88	-0,66	4,04E-02	5,74E-02	glutathione biosynthetic process /// glutathione biosynthetic process /// response to DNA damage stimulus	catalytic activity /// glutathione synthase activity /// glutathione synthase activity /// ATP binding /// ligase activity
CG9624	29,9	47,5	62,1	0,67	1,05	0,39	7,95E-02	3,18E-01	glycolysis	magnesium ion binding /// pyruvate kinase activity /// protein binding /// potassium ion binding
CG6401	40,8	77,5	88,6	0,93	1,12	0,19	5,63E-03	6,74E-02	GPI anchor biosynthetic process /// GPI anchor biosynthetic process /// biosynthetic process	transferase activity /// transferase activity, transferring glycosyl groups /// phosphatidylinositol N-acetylglucosaminyltransferase activity /// phosphatidylinositol N-acetylglucosaminyltransferase activity
shade	79,2	114,1	187,7	0,53	1,25	0,72	3,94E-02	4,34E-02	growth of a germarium-derived egg chamber /// dorsal closure /// central nervous system development /// midgut development /// head involution /// ecdysteroid biosynthetic process /// ecdysteroid biosynthetic process /// oxidation reduction	monooxygenase activity /// ecdysone 20-monooxygenase activity /// ecdysone 20-monooxygenase activity /// iron ion binding /// electron carrier activity /// electron carrier activity /// electron carrier activity /// oxidoreductase activity /// heme binding /// metal ion binding
CG12120	32,0	47,4	74,3	0,57	1,21	0,65	2,91E-02	1,12E-01	histamine metabolic process /// histamine biosynthetic process /// visual perception /// flight behavior /// penicillin biosynthetic process /// dopamine biosynthetic process /// cuticle pigmentation	beta-alanyl-dopamine hydrolase activity /// beta-alanyl-dopamine hydrolase activity /// hydrolase activity /// hydrolase activity /// beta-alanyl-histamine hydrolase activity
Jonah 99Fi	1283,6	3743,6	3150,5	1,54	1,30	-0,25	2,75E-03	1,46E-01	instar larval development /// proteolysis /// haltere development /// molting cycle, chitin-based cuticle	sequence-specific DNA binding transcription factor activity /// catalytic activity /// serine-type endopeptidase activity /// serine-type endopeptidase activity /// peptidase activity /// serine-type peptidase activity /// hydrolase activity
CG32295	20,7	29,9	45,7	0,53	1,14	0,61	5,67E-02	2,52E-01	inter-male aggressive behavior /// olfactory behavior	protein binding /// protein phosphatase 1 binding
CG6296	26,6	195,7	78,1	2,88	1,56	-1,32	1,60E-02	1,02E-01	lipid metabolic process	catalytic activity /// triglyceride lipase activity /// triglyceride lipase activity /// phospholipase A1 activity /// hydrolase activity
CG6271 /// CG6277	231,4	1058,5	839,6	2,19	1,86	-0,33	1,69E-02	2,09E-01	lipid metabolic process	catalytic activity /// triglyceride lipase activity /// triglyceride lipase activity /// protein binding /// phospholipase A1 activity /// hydrolase activity
CG11029	117,8	221,6	227,9	0,91	0,95	0,04	3,87E-03	4,25E-01	lipid metabolic process	lysophospholipase activity /// lysophospholipase activity /// lipase activity /// hydrolase activity /// hydrolase activity, acting on ester bonds
Lipid storage droplet-2	176,2	521,0	631,9	1,56	1,84	0,28	6,98E-03	4,11E-02	lipid metabolic process /// transport /// lipid transport /// lipid transport /// multicellular organismal development /// lipid storage /// lipid storage /// lipid storage /// sequestering of triglyceride /// lipid particle transport along microtubule /// regulation of transport	protein binding
Menl-2	35,3	43,1	72,0	0,29	1,03	0,74	6,13E-02	1,89E-01	malate metabolic process /// metabolic process /// oxidation reduction	catalytic activity /// malic enzyme activity /// malate dehydrogenase (oxaloacetate-decarboxylating) (NADP+) activity /// malate dehydrogenase (oxaloacetate-decarboxylating) (NADP+) activity /// binding /// oxidoreductase activity /// oxidoreductase activity, acting on the CH-OH group of donors, NAD or NADP as acceptor /// metal ion binding /// NAD or NADH binding
CG3950	89,9	133,8	179,8	0,57	1,00	0,43	3,20E-03	3,36E-03	mesoderm development	actin binding /// structural constituent of cytoskeleton
CG15450	21,1	32,9	39,8	0,64	0,91	0,27	6,59E-02	3,10E-01	metabolic process	1-acylglycerol-3-phosphate O-acyltransferase activity /// 1-acylglycerol-3-phosphate O-acyltransferase activity /// protein binding /// acyltransferase activity /// transferase activity
CG5656	39,0	95,4	99,8	1,29	1,36	0,06	1,63E-02	4,06E-01	metabolic process	catalytic activity /// alkaline phosphatase activity /// alkaline phosphatase activity /// hydrolase activity /// phosphatase activity
Ugt36Bc	64,1	30,7	147,3	-1,06	1,20	2,26	3,68E-03	5,48E-03	metabolic process	glucuronosyltransferase activity /// glucuronosyltransferase activity /// transferase activity, transferring hexosyl groups
Lysozyme P	4,0	148,0	179,3	5,20	5,47	0,28	2,35E-03	1,85E-01	metabolic process /// cell wall macromolecule catabolic process /// antimicrobial humoral response /// cytolysis /// defense response to bacterium	lysozyme activity /// lysozyme activity /// lysozyme activity /// catalytic activity /// hydrolase activity /// hydrolase activity, acting on glycosyl bonds
CG4860	152,5	370,2	335,4	1,28	1,14	-0,14	1,64E-03	2,39E-01	metabolic process /// oxidation reduction	acyl-CoA dehydrogenase activity /// acyl-CoA dehydrogenase activity /// oxidoreductase activity /// oxidoreductase activity, acting on the CH-CH group of donors /// FAD or FADH2 binding
CG10006	142,0	276,8	300,8	0,96	1,08	0,12	2,40E-03	1,43E-01	metal ion transport /// transmembrane transport	metal ion transmembrane transporter activity
Ejaculatory bulb protein III /// PebIII	32,9	127,2	73,0	1,95	1,15	-0,80	1,65E-03	2,57E-03	metamorphosis /// behavior /// response to virus /// post-mating behavior	protein binding
Tektin A	46,2	50,0	103,2	0,11	1,16	1,05	7,19E-02	1,03E-01	microtubule cytoskeleton organization /// microtubule-based process	protein binding /// microtubule binding
robl62A	66,1	132,6	139,4	1,01	1,08	0,07	4,30E-02	4,70E-01	microtubule-based movement	protein binding /// hydrolase activity /// ATPase activity, coupled /// ATPase activity, uncoupled
CG32392	47,4	72,5	85,4	0,61	0,85	0,24	6,23E-02	3,84E-01	microtubule-based process	protein binding /// microtubule binding
Rbp1-like	27,8	63,2	61,4	1,18	1,14	-0,04	1,77E-02	3,35E-01	nuclear mRNA splicing, via spliceosome /// nuclear mRNA splicing, via spliceosome /// mRNA splice site selection /// RNA splicing	nucleotide binding /// nucleic acid binding
Cyp6a21	9,9	56,5	50,6	2,52	2,36	-0,16	2,14E-02	2,60E-01	oxidation reduction	monooxygenase activity /// iron ion binding /// electron carrier activity /// electron carrier activity /// electron carrier activity /// oxidoreductase activity /// heme binding /// metal ion binding
Cyp6a17	45,3	99,8	82,0	1,14	0,86	-0,28	9,95E-03	1,62E-01	oxidation reduction	monooxygenase activity /// iron ion binding /// electron carrier activity /// electron carrier activity /// electron carrier activity /// oxidoreductase activity /// heme binding /// metal ion binding
Cyp28d1	132,9	168,1	265,9	0,34	1,00	0,66	3,11E-02	5,59E-02	oxidation reduction	monooxygenase activity /// iron ion binding /// electron carrier activity /// electron carrier activity /// electron carrier activity /// oxidoreductase activity /// heme binding /// metal ion binding
Cytochrome P450-9c1	6,8	97,7	127,6	3,85	4,23	0,39	8,78E-04	7,20E-02	oxidation reduction	monooxygenase activity /// iron ion binding /// electron carrier activity /// electron carrier activity /// electron carrier activity /// oxidoreductase activity /// heme binding /// metal ion binding
Cyp6a14	116,3	620,1	638,8	2,41	2,46	0,04	4,24E-03	3,64E-01	oxidation reduction	monooxygenase activity /// iron ion binding /// electron carrier activity /// oxidoreductase activity /// heme binding /// metal ion binding
Cytochrome P450-9b2	402,0	1427,8	1019,2	1,83	1,34	-0,49	8,42E-04	5,68E-02	oxidation reduction	monooxygenase activity /// iron ion binding /// protein binding /// electron carrier activity /// electron carrier activity /// electron carrier activity /// oxidoreductase activity /// heme binding /// metal ion binding
CG3270	151,7	419,3	380,1	1,47	1,33	-0,14	1,10E-03	1,79E-01	oxidation reduction	oxidoreductase activity
CG31370	21,1	94,2	84,6	2,16	2,00	-0,16	5,43E-03	1,70E-01	oxidation reduction	oxidoreductase activity
CG15093	26,1	78,7	83,8	1,59	1,68	0,09	2,18E-03	7,47E-02	pentose-phosphate shunt /// cellular amino acid metabolic process /// valine metabolic process /// metabolic process /// oxidation reduction	catalytic activity /// phosphogluconate dehydrogenase (decarboxylating) activity /// binding /// protein binding /// 3-hydroxyisobutyrate dehydrogenase activity /// 3-hydroxyisobutyrate dehydrogenase activity /// 3-hydroxyisobutyrate dehydrogenase activity /// oxidoreductase activity /// coenzyme binding /// NAD or NADH binding
CG6652	59,6	72,1	129,0	0,27	1,11	0,84	7,49E-02	2,13E-01	phagocytosis, engulfment	protein binding
Phosphotidylinositol 3 kinase 68D	70,8	135,6	144,6	0,94	1,03	0,09	6,82E-03	3,01E-01	phagocytosis, engulfment /// response to DNA damage stimulus /// cell communication /// phosphoinositide phosphorylation /// phosphoinositide phosphorylation /// phosphoinositide phosphorylation /// phosphoinositide phosphorylation /// phosphoinositide-mediated signaling	inositol or phosphatidylinositol kinase activity /// binding /// protein binding /// phospholipid binding /// kinase activity /// 1-phosphatidylinositol-3-kinase activity /// 1-phosphatidylinositol-3-kinase activity /// 1-phosphatidylinositol-3-kinase activity /// transferase activity /// phosphotransferase activity, alcohol group as acceptor /// phosphoinositide 3-kinase activity /// phosphoinositide 3-kinase activity /// phosphatidylinositol-4-phosphate 3-kinase activity /// phosphatidylinositol-4-phosphate 3-kinase activity /// phosphoinositide binding /// phosphatidylinositol-4,5-bisphosphate 3-kinase activity /// phosphatidylinositol-4,5-bisphosphate 3-kinase activity
CG5840	55,3	146,0	174,7	1,40	1,66	0,26	1,92E-02	2,51E-01	proline biosynthetic process /// metabolic process /// cellular amino acid biosynthetic process /// oxidation reduction	catalytic activity /// pyrroline-5-carboxylate reductase activity /// pyrroline-5-carboxylate reductase activity /// binding /// protein binding /// oxidoreductase activity /// identical protein binding
Ptpmeg	148,9	393,1	418,3	1,40	1,49	0,09	1,03E-02	3,44E-01	protein dephosphorylation /// dephosphorylation	phosphoprotein phosphatase activity /// protein tyrosine phosphatase activity /// binding /// protein binding /// cytoskeletal protein binding /// hydrolase activity /// phosphatase activity

**Heat shock protein cognate 2**	61,9	91,3	151,0	0,56	1,29	0,73	1,06E-03	7,81E-02	protein folding /// response to stress /// response to heat	nucleotide binding /// ATP binding /// unfolded protein binding
**CG10478**	13,4	36,9	41,3	1,46	1,63	0,16	1,45E-02	3,71E-01	protein import into nucleus	binding /// protein binding /// protein transmembrane transporter activity
**bicoid-interacting protein 3**	47,9	102,8	98,7	1,10	1,04	-0,06	4,38E-02	2,75E-01	protein methylation /// developmental process /// olfactory behavior /// regulation of transcription	protein binding /// methyltransferase activity /// methyltransferase activity /// transferase activity
**CG16716**	45,9	68,9	104,7	0,59	1,19	0,60	7,56E-02	2,53E-01	protein modification process	tubulin-tyrosine ligase activity /// tubulin-tyrosine ligase activity /// ligase activity
**CG32238**	40,9	61,7	71,0	0,59	0,79	0,20	4,24E-02	3,96E-01	protein modification process /// C-terminal protein-tyrosinylation	tubulin-tyrosine ligase activity /// tubulin-tyrosine ligase activity /// protein binding /// ligase activity
**CG8565**	133,8	195,2	271,7	0,55	1,02	0,48	4,60E-02	2,74E-01	protein phosphorylation /// protein phosphorylation	nucleotide binding /// protein kinase activity /// protein kinase activity /// protein kinase activity /// protein serine/threonine kinase activity /// protein serine/threonine kinase activity /// ATP binding /// transferase activity
**CG2577**	12,6	19,6	21,2	0,64	0,76	0,12	1,85E-01	4,45E-01	protein phosphorylation /// protein phosphorylation	nucleotide binding /// protein kinase activity /// protein serine/threonine kinase activity /// protein serine/threonine kinase activity /// ATP binding /// transferase activity
**Puromycin sensitive aminopeptidase**	565,5	1032,7	1350,0	0,87	1,26	0,39	2,81E-03	5,07E-04	proteolysis	aminopeptidase activity /// aminopeptidase activity /// metallopeptidase activity /// zinc ion binding /// hydrolase activity
**CG30375**	17,7	47,0	63,1	1,41	1,84	0,42	9,85E-03	1,17E-01	proteolysis	catalytic activity /// serine-type endopeptidase activity /// hydrolase activity
**CG13527**	16,3	28,8	36,3	0,82	1,16	0,34	4,10E-02	3,43E-01	proteolysis	catalytic activity /// serine-type endopeptidase activity /// serine-type endopeptidase activity /// hydrolase activity
**Kuzbanian-like**	38,8	82,6	100,1	1,09	1,37	0,28	4,44E-02	2,69E-01	proteolysis	metalloendopeptidase activity /// metalloendopeptidase activity /// peptidase activity /// metallopeptidase activity /// zinc ion binding /// hydrolase activity /// metal ion binding
**CG33013**	18,7	63,3	57,8	1,76	1,63	-0,13	1,20E-03	2,46E-01	proteolysis	peptidase activity
**CG10051**	2,5	22,6	19,1	3,19	2,95	-0,24	4,72E-03	2,11E-01	proteolysis	peptidase activity /// hydrolase activity
**CG9850**	192,9	358,3	388,1	0,89	1,01	0,12	3,66E-02	3,11E-01	proteolysis /// cell proliferation	metalloendopeptidase activity /// protein binding /// peptidase activity /// metallopeptidase activity /// hydrolase activity
**CG32063**	257,5	348,9	533,2	0,44	1,05	0,61	4,27E-02	2,50E-01	proteolysis /// protein metabolic process	aminopeptidase activity /// aminopeptidase activity /// metalloexopeptidase activity /// hydrolase activity /// manganese ion binding
**Jonah 25Biii**	182,4	3370,1	2025,4	4,21	3,47	-0,73	1,55E-02	3,48E-02	proteolysis /// proteolysis	catalytic activity /// endopeptidase activity /// serine-type endopeptidase activity /// hydrolase activity
**CG7142**	26,2	64,7	57,1	1,31	1,12	-0,18	5,39E-03	2,76E-01	proteolysis /// proteolysis	catalytic activity /// serine-type endopeptidase activity /// serine-type endopeptidase activity /// hydrolase activity
**CG11911**	79,9	445,7	238,5	2,48	1,58	-0,90	3,90E-02	6,97E-02	proteolysis /// proteolysis	catalytic activity /// serine-type endopeptidase activity /// serine-type endopeptidase activity /// peptidase activity /// serine-type peptidase activity /// hydrolase activity
**CG11529**	156,8	621,8	493,0	1,99	1,65	-0,33	3,89E-03	5,96E-03	proteolysis /// proteolysis	catalytic activity /// serine-type endopeptidase activity /// serine-type endopeptidase activity /// peptidase activity /// serine-type peptidase activity /// hydrolase activity
**CG10405**	38,7	75,3	83,6	0,96	1,11	0,15	3,22E-02	3,10E-01	proteolysis /// proteolysis	catalytic activity /// serine-type endopeptidase activity /// serine-type endopeptidase activity /// peptidase activity /// serine-type peptidase activity /// hydrolase activity
**CG17571**	59,4	477,1	288,3	3,00	2,28	-0,73	2,92E-03	5,02E-02	proteolysis /// proteolysis	catalytic activity /// serine-type endopeptidase activity /// serine-type endopeptidase activity /// peptidase activity /// serine-type peptidase activity /// hydrolase activity
**CG12951**	1407,5	2910,3	2568,2	1,05	0,87	-0,18	4,50E-02	1,75E-01	proteolysis /// proteolysis	catalytic activity /// serine-type endopeptidase activity /// serine-type endopeptidase activity /// peptidase activity /// serine-type peptidase activity /// hydrolase activity
**Jonah 44E**	88,7	1068,8	701,1	3,59	2,98	-0,61	1,53E-02	7,76E-02	proteolysis /// proteolysis	catalytic activity /// serine-type endopeptidase activity /// serine-type endopeptidase activity /// serine-type endopeptidase activity /// peptidase activity /// serine-type peptidase activity /// hydrolase activity
**Jonah 65Aii**	1016,2	2699,6	2157,8	1,41	1,09	-0,32	7,41E-02	1,37E-01	proteolysis /// proteolysis	catalytic activity /// serine-type endopeptidase activity /// serine-type endopeptidase activity /// serine-type endopeptidase activity /// peptidase activity /// serine-type peptidase activity /// hydrolase activity
**Jonah 66Ci**	565,4	1900,0	1557,5	1,75	1,46	-0,29	2,07E-02	2,33E-01	proteolysis /// proteolysis /// cyclic nucleotide biosynthetic process	catalytic activity /// adenylate cyclase activity /// endopeptidase activity /// serine-type endopeptidase activity /// serine-type endopeptidase activity /// hydrolase activity /// phosphorus-oxygen lyase activity
**CG9733**	16,5	59,5	64,3	1,85	1,97	0,11	1,53E-04	1,17E-01	proteolysis /// proteolysis /// defense response	catalytic activity /// serine-type endopeptidase activity /// serine-type endopeptidase activity /// serine-type endopeptidase activity /// peptidase activity /// serine-type peptidase activity /// hydrolase activity
**S1P**	27,7	67,9	57,1	1,29	1,04	-0,25	3,53E-02	1,99E-01	proteolysis /// sterol regulatory element binding protein cleavage	serine-type endopeptidase activity /// serine-type endopeptidase activity /// serine-type endopeptidase activity /// hydrolase activity
**Pros5R1**	23,5	32,6	38,1	0,47	0,70	0,22	5,71E-02	4,03E-01	proteolysis involved in cellular protein catabolic process	endopeptidase activity /// endopeptidase activity /// threonine-type endopeptidase activity /// protein binding /// peptidase activity /// hydrolase activity
**Ecdysone-induced gene 71Ee**	1270,9	3384,8	3708,5	1,41	1,55	0,13	6,72E-03	1,84E-01	puparial adhesion /// hemolymph coagulation /// hemolymph coagulation	structural molecule activity /// protein binding
**CG7742**	58,6	83,8	137,8	0,52	1,23	0,72	3,81E-02	1,94E-01	regulation of Rab GTPase activity	Rab GTPase activator activity /// protein binding
**CG32006**	24,6	41,8	37,7	0,76	0,61	-0,15	9,96E-02	4,36E-01	regulation of transcription, DNA-dependent /// regulation of transcription, DNA-dependent	DNA binding /// sequence-specific DNA binding transcription factor activity /// sequence-specific DNA binding transcription factor activity /// sequence-specific DNA binding
**Lk6**	303,5	463,9	676,7	0,61	1,16	0,54	2,15E-03	2,40E-02	regulation of translation /// protein phosphorylation /// protein phosphorylation /// response to stress /// microtubule-based process /// regulation of growth	nucleotide binding /// protein kinase activity /// protein kinase activity /// protein kinase activity /// protein serine/threonine kinase activity /// protein serine/threonine kinase activity /// protein binding /// ATP binding /// kinase activity /// kinase activity
**CG4461**	7,6	22,0	33,4	1,53	2,13	0,60	1,46E-02	6,28E-03	response to heat	---
**Cyp12a4**	103,6	340,8	231,7	1,72	1,16	-0,56	2,55E-03	3,48E-02	response to insecticide /// oxidation reduction	monooxygenase activity /// iron ion binding /// protein binding /// electron carrier activity /// electron carrier activity /// electron carrier activity /// oxidoreductase activity /// heme binding /// metal ion binding
**lethal (2) essential for life**	1027,4	1698,1	2815,5	0,72	1,45	0,73	3,32E-03	2,66E-03	response to stress /// multicellular organismal development /// response to heat /// response to heat /// embryo development	identical protein binding
**Hsp67Bb**	146,0	502,8	412,7	1,78	1,50	-0,28	1,97E-02	2,58E-01	response to stress /// response to oxidative stress /// determination of adult lifespan /// response to heat /// response to heat /// response to heat /// response to heat /// protein refolding	---
**scaf6**	25,8	35,3	57,0	0,45	1,14	0,69	1,95E-02	1,89E-03	RNA processing	nucleic acid binding /// RNA binding
**CG6954**	142,2	357,2	367,6	1,33	1,37	0,04	1,72E-02	4,51E-01	signal transduction	---
**CG13790**	12,3	30,9	37,2	1,33	1,60	0,27	5,83E-02	2,33E-01	signal transduction /// Ras protein signal transduction /// regulation of small GTPase mediated signal transduction	GTPase activator activity /// Ras GTPase activator activity
**CG18445**	426,4	1076,4	1034,0	1,34	1,28	-0,06	6,92E-03	2,87E-01	sperm individualization /// germ cell migration	---
**yuri gagarin**	12,3	17,9	19,0	0,54	0,63	0,09	1,41E-01	4,53E-01	sperm individualization /// microtubule basal body organization /// gravitaxis	nucleotide binding /// protein binding
**ACXC**	23,6	35,6	52,0	0,59	1,14	0,55	4,74E-02	1,90E-01	spermatogenesis /// cyclic nucleotide biosynthetic process /// intracellular signaling pathway	adenylate cyclase activity /// adenylate cyclase activity /// adenylate cyclase activity /// adenylate cyclase activity /// lyase activity /// phosphorus-oxygen lyase activity
**defective transmitter release**	30,8	41,6	63,3	0,43	1,04	0,60	8,44E-02	2,49E-01	synaptic transmission /// cilium morphogenesis	protein binding /// dynein binding
**CG9589**	28,1	38,0	58,1	0,44	1,05	0,61	1,01E-01	2,67E-01	tetrapyrrole biosynthetic process	uroporphyrinogen-III synthase activity /// uroporphyrinogen-III synthase activity /// lyase activity
**Ecdysone-induced protein 74EF**	70,4	905,7	496,6	3,68	2,82	-0,87	4,69E-03	4,53E-03	transcription /// regulation of transcription, DNA-dependent /// regulation of transcription, DNA-dependent /// autophagy /// multicellular organismal development /// cell death /// cellular process /// salivary gland cell autophagic cell death /	DNA binding /// sequence-specific DNA binding transcription factor activity /// sequence-specific DNA binding transcription factor activity /// specific RNA polymerase II transcription factor activity /// sequence-specific DNA binding
**CG4810**	33,2	47,9	59,5	0,53	0,84	0,31	3,26E-02	3,46E-01	translation /// translational initiation /// translational initiation /// regulation of translational initiation	translation initiation factor activity /// translation initiation factor activity /// protein binding
**CG5348**	11,8	18,0	27,8	0,62	1,24	0,62	5,37E-02	1,68E-01	transmembrane transport	---
**CG8083**	24,3	63,8	51,6	1,39	1,09	-0,31	1,81E-02	4,72E-02	transport	nucleoside binding /// nucleoside:sodium symporter activity /// nucleoside:sodium symporter activity
**CG11147**	48,5	101,4	117,5	1,06	1,28	0,21	8,45E-03	1,54E-01	transport	nucleotide binding /// transporter activity /// ATP binding /// hydrolase activity /// ATPase activity /// nucleoside-triphosphatase activity /// ATPase activity, uncoupled
**CG14077**	23,0	36,0	58,0	0,65	1,34	0,69	7,20E-02	2,41E-01	transport /// ion transport /// oxidation reduction	cytochrome-c oxidase activity /// cytochrome-c oxidase activity /// receptor activity /// ionotropic glutamate receptor activity /// ion channel activity /// extracellular-glutamate-gated ion channel activity
**CG18418**	50,9	68,3	112,6	0,43	1,15	0,72	4,76E-02	1,81E-01	transport /// mitochondrial transport /// mitochondrial transport /// alpha-ketoglutarate transport /// malate transport	binding /// oxoglutarate:malate antiporter activity /// transmembrane transporte activity
**CG18317**	65,8	73,1	138,2	0,15	1,07	0,92	5,04E-03	5,17E-02	transport /// mitochondrial transport /// transmembrane transport	binding /// transmembrane transporter activity
**CG2930**	82,5	268,9	202,5	1,71	1,30	-0,41	1,01E-02	4,17E-02	transport /// oligopeptide transport	transporter activity /// proton-dependent oligopeptide secondary active transmembrane transporter activity /// protein binding
**Odorant-binding protein 99a /// Obp99a**	330,5	589,3	856,2	0,83	1,37	0,55	5,51E-03	8,70E-03	transport /// sensory perception of chemical stimulus /// sensory perception of chemical stimulus /// sensory perception of smell /// response to pheromone /// olfactory behavior /// response to stimulus	protein binding /// odorant binding /// odorant binding /// odorant binding /// odorant binding
**CG12201**	11,3	17,1	13,7	0,59	0,27	-0,32	3,78E-01	3,95E-01	transport /// transmembrane transport	transporter activity /// binding /// carnitine transporter activity /// acyl carnitine transporter activity /// transmembrane transporter activity
**CG16727**	64,3	283,6	142,6	2,14	1,15	-0,99	2,99E-03	3,01E-02	transport /// transmembrane transport	transporter activity /// organic cation transmembrane transporter activity
**CG31874**	7,8	28,7	39,6	1,89	2,35	0,46	3,56E-02	3,29E-01	tricarboxylic acid cycle /// fumarate metabolic process	catalytic activity /// fumarate hydratase activity /// fumarate hydratase activity /// lyase activity
**CG7349**	76,0	101,2	177,9	0,41	1,23	0,81	4,11E-02	2,22E-01	tricarboxylic acid cycle /// oxidation reduction	succinate dehydrogenase activity /// succinate dehydrogenase activity /// protein binding /// electron carrier activity /// oxidoreductase activity /// iron-sulfur cluster binding
**CG6629**	35,1	47,9	71,7	0,45	1,03	0,58	8,01E-02	2,72E-01	tricarboxylic acid cycle /// succinate metabolic process /// mitochondrial electron transport, succinate to ubiquinone /// oxidation reduction	succinate dehydrogenase activity /// succinate dehydrogenase activity /// protein binding /// oxidoreductase activity /// oxidoreductase activity, acting on the CH-CH group of donors
**CG8369**	2217,7	2048,2	4535,4	-0,11	1,03	1,15	1,14E-02	2,59E-03	wing disc dorsal/ventral pattern formation	---

Repression

Gene Title	w1118 average of 3 replicates	dAda2b average of 3 replictes	Ada2bS average of 3 replicates	log2(dAda2b/w1118)	log2(dAda2bS/w1118)	log2(dAda2bS/Ada2b)	p value w1118 vs Ada2bS	p value Ada2b vs Ada2bS	go biological process term	go molecular function term
CG15820	84,2	39,2	38,5	-1,10	-1,13	-0,02	5,32E-03	4,40E-01	---	---
CG31531	47,0	29,0	22,2	-0,70	-1,08	-0,39	3,88E-02	5,27E-02	---	---
CG7179	29,7	18,8	14,9	-0,66	-1,00	-0,34	2,56E-02	1,30E-01	---	---
CG6327	137,5	41,4	58,7	-1,73	-1,23	0,50	1,76E-02	1,65E-02	---	---
CG1109	66,8	19,0	16,5	-1,81	-2,02	-0,20	6,45E-04	3,37E-01	---	---
CG32115	153,3	57,2	52,0	-1,42	-1,56	-0,14	2,20E-02	1,66E-01	---	---
CG15905	126,6	70,4	58,1	-0,85	-1,12	-0,28	1,45E-03	4,63E-02	---	---
CG15535	75,1	29,2	34,0	-1,36	-1,14	0,22	3,53E-03	1,91E-01	---	---
Tetraspanin 42Ee	1730,5	923,4	839,5	-0,91	-1,04	-0,14	8,83E-03	1,83E-01	---	---
CG13026	174,4	12,2	54,4	-3,84	-1,68	2,16	3,49E-02	7,06E-02	---	---
CG14253	158,0	87,2	73,3	-0,86	-1,11	-0,25	3,02E-03	1,19E-01	---	---
CG3568	71,6	39,6	32,3	-0,86	-1,15	-0,29	2,26E-03	2,08E-01	---	---
HDC07203	338,5	5,6	121,7	-5,92	-1,48	4,44	1,10E-01	1,34E-01	---	---
CG18343	591,8	419,1	223,3	-0,50	-1,41	-0,91	1,24E-02	1,61E-02	---	---
CG2016	1040,9	437,5	495,2	-1,25	-1,07	0,18	2,75E-03	3,18E-02	---	---
CG8270	82,8	45,1	47,1	-0,88	-0,81	0,06	1,15E-03	4,15E-01	---	---
CG30424	88,3	55,1	38,8	-0,68	-1,19	-0,51	1,90E-02	1,59E-01	---	---
CG18577	40,0	6,0	5,2	-2,74	-2,94	-0,21	3,20E-03	3,56E-01	---	---
Ecdysone-induced gene 71Ec	1985,6	440,7	627,6	-2,17	-1,66	0,51	3,23E-02	1,86E-01	---	---
CG16743	119,3	58,2	48,1	-1,04	-1,31	-0,27	1,76E-02	2,34E-01	---	---
Mec2	502,2	361,5	218,2	-0,47	-1,20	-0,73	4,63E-03	2,61E-02	---	---
CG7173	20,4	6,5	6,2	-1,65	-1,71	-0,06	1,22E-02	3,94E-01	---	---
CG11671	23,2	26,1	9,4	0,17	-1,30	-1,47	6,90E-03	5,95E-02	---	---
CG12813	60,8	44,7	24,9	-0,44	-1,29	-0,84	2,90E-02	1,04E-01	---	---
CG9682	386,2	340,1	170,8	-0,18	-1,18	-0,99	1,76E-02	1,46E-02	---	---
CG13315	1674,4	177,1	141,4	-3,24	-3,57	-0,33	2,95E-03	3,17E-01	---	---
CG13028	45,3	19,1	10,1	-1,24	-2,17	-0,93	9,66E-03	2,94E-02	---	---
CG17352	59,0	9,0	9,2	-2,71	-2,69	0,03	5,34E-03	4,85E-01	---	---
CG8012	86,5	29,4	20,8	-1,56	-2,06	-0,50	3,12E-02	6,91E-02	---	---
CG13053	21,3	1,5	6,7	-3,83	-1,66	2,17	6,23E-02	1,21E-01	---	---
CG18622	325,5	175,5	122,5	-0,89	-1,41	-0,52	7,51E-03	9,18E-02	---	---
CG33054	22,8	8,2	9,3	-1,48	-1,29	0,19	1,09E-02	2,17E-01	---	---
CG1572	540,4	349,5	256,2	-0,63	-1,08	-0,45	1,74E-02	2,92E-02	---	---
CG8157	196,3	151,8	91,1	-0,37	-1,11	-0,74	7,38E-02	1,47E-01	---	---
CG3984	35,7	9,4	13,3	-1,92	-1,42	0,50	3,19E-02	2,69E-01	---	---
CG7705	121,9	72,9	58,8	-0,74	-1,05	-0,31	8,48E-03	7,39E-02	---	---
putative noncoding RNA 009:3L	120,1	33,8	30,0	-1,83	-2,00	-0,17	2,29E-02	2,20E-01	---	---
CG6234	836,4	470,4	408,0	-0,83	-1,04	-0,21	2,63E-03	3,77E-02	---	---
Ecdysone-inducible gene L1	181,2	69,1	86,1	-1,39	-1,07	0,32	6,17E-02	4,79E-02	---	---
CG14567	269,0	106,1	124,4	-1,34	-1,11	0,23	4,02E-03	1,06E-01	---	---
CG3153	630,9	275,0	241,9	-1,20	-1,38	-0,18	2,96E-02	3,28E-01	---	---
CG14265	192,1	81,0	54,4	-1,25	-1,82	-0,57	8,96E-03	2,30E-01	---	---
CG32212	26,4	4,3	5,6	-2,63	-2,23	0,40	5,68E-02	2,58E-01	---	---
CG6739	47,4	13,7	11,6	-1,79	-2,04	-0,24	1,61E-02	3,62E-01	---	---
CG33169	46,7	30,6	23,3	-0,61	-1,00	-0,39	4,04E-03	1,00E-02	---	---
CG15385	32,8	28,9	15,8	-0,18	-1,05	-0,87	5,03E-02	2,36E-02	---	acid phosphatase activity /// hydrolase activity
CG11170	190,5	96,6	88,2	-0,98	-1,11	-0,13	9,97E-03	2,87E-01	---	ATP binding /// hydrolase activity /// ATPase activity /// ATPase activity, uncoupled
CG14872	105,9	16,5	11,6	-2,68	-3,19	-0,51	5,20E-03	1,16E-01	---	binding
CG14500	136,1	68,4	30,8	-0,99	-2,14	-1,15	4,97E-03	4,44E-02	---	binding
CG17556	433,6	313,6	200,1	-0,47	-1,12	-0,65	1,53E-02	4,69E-02	---	binding
CG7763	94,7	38,9	39,5	-1,28	-1,26	0,02	1,40E-02	4,89E-01	---	binding
CG11436	57,2	37,9	27,2	-0,59	-1,07	-0,48	1,01E-02	6,10E-03	---	binding
CG5399	1123,7	703,8	474,4	-0,68	-1,24	-0,57	1,07E-03	2,73E-02	---	binding
CG31955	143,9	91,1	50,1	-0,66	-1,52	-0,86	1,00E-02	1,46E-02	---	binding
CG4495	293,4	259,1	138,8	-0,18	-1,08	-0,90	5,42E-03	1,22E-02	---	calcium ion binding
Esterase-8	115,4	61,6	55,5	-0,91	-1,06	-0,15	3,63E-02	2,85E-01	---	carboxylesterase activity /// carboxylesterase activity /// carboxylesterase activity /// hydrolase activity
CG6018	36,0	26,1	16,0	-0,46	-1,17	-0,71	2,66E-03	1,03E-02	---	carboxylesterase activity /// carboxylesterase activity /// hydrolase activity
CG3835	40,0	21,7	20,9	-0,89	-0,94	-0,05	2,73E-02	1,19E-01	---	catalytic activity /// oxidoreductase activity /// FAD or FADH2 binding
CG6357	418,3	241,5	189,7	-0,79	-1,14	-0,35	7,15E-03	1,93E-01	---	cysteine-type endopeptidase activity /// hydrolase activity
Glutathione S transferase D10	63,1	41,7	30,5	-0,60	-1,05	-0,45	3,15E-04	2,50E-01	---	glutathione transferase activity /// glutathione transferase activity /// glutathione transferase activity /// transferase activity
Glutathione S transferase D3	127,9	48,9	49,9	-1,39	-1,36	0,03	4,82E-02	4,75E-01	---	glutathione transferase activity /// glutathione transferase activity /// glutathione transferase activity /// transferase activity
CG6630	22,8	13,7	15,0	-0,73	-0,61	0,12	6,11E-02	3,73E-01	---	guanyl-nucleotide exchange factor activity /// GTP binding /// GTPase binding
CG9646	204,0	85,7	47,8	-1,25	-2,09	-0,84	7,56E-03	1,05E-02	---	hydrolase activity
CG7900	61,9	7,4	10,7	-3,07	-2,53	0,54	4,59E-04	1,10E-01	---	ligase activity /// carbon-nitrogen ligase activity, with glutamine as amido-N-donor /// fatty acid amide hydrolase activity
Myosin light chain 2	20,8	14,2	9,9	-0,55	-1,07	-0,52	2,38E-03	6,86E-02	---	microfilament motor activity /// motor activity /// calcium ion binding /// calcium ion binding /// protein binding /// hydrolase activity /// ATPase activity, coupled /// ATPase activity, uncoupled
CG6839	27,3	28,6	10,1	0,07	-1,44	-1,50	6,52E-02	6,51E-02	---	nucleic acid binding /// hydrolase activity /// metal ion binding
CG14062	37,7	2,6	3,9	-3,88	-3,26	0,62	3,40E-02	2,20E-01	---	nucleic acid binding /// hydrolase activity /// metal ion binding
jim	264,6	153,9	121,8	-0,78	-1,12	-0,34	4,32E-02	2,31E-01	---	nucleic acid binding /// sequence-specific DNA binding transcription factor activity /// zinc ion binding
CG12391	130,9	51,9	57,2	-1,33	-1,19	0,14	1,08E-03	2,55E-01	---	nucleic acid binding /// zinc ion binding
CG9107	26,3	17,2	9,6	-0,61	-1,45	-0,84	2,40E-02	5,57E-02	---	nucleotide binding /// nucleic acid binding /// protein binding
CG14933	506,1	214,6	119,2	-1,24	-2,09	-0,85	1,07E-02	2,67E-02	---	protein binding
CG5333	46,5	36,8	22,1	-0,34	-1,08	-0,74	2,04E-02	1,35E-01	---	protein binding
Osiris 6	114,6	7,0	36,3	-4,03	-1,66	2,38	2,34E-01	2,14E-01	---	protein binding
CG4040 /// CG33224	61,7	39,1	29,8	-0,66	-1,05	-0,39	7,42E-02	1,01E-01	---	protein binding
CG9304	599,7	334,5	200,0	-0,84	-1,58	-0,74	1,53E-03	9,95E-02	---	protein binding
CG6834	33,0	18,8	16,2	-0,81	-1,02	-0,21	1,19E-02	3,37E-01	---	protein binding
CG9497	74,4	7,5	8,9	-3,31	-3,06	0,25	5,37E-03	4,08E-01	---	protein binding
CG32264	334,1	175,4	192,1	-0,93	-0,80	0,13	2,91E-02	2,61E-01	---	protein binding
CG3884	69,3	51,1	28,2	-0,44	-1,30	-0,86	3,12E-03	2,41E-02	---	protein binding
CG14534	134,1	54,2	66,4	-1,31	-1,01	0,29	7,48E-03	2,07E-02	---	protein binding
CG14193	46,4	19,5	22,3	-1,25	-1,05	0,19	1,97E-02	2,41E-01	---	protein binding
CG6912	26,9	4,3	12,8	-2,66	-1,07	1,59	1,83E-02	3,01E-02	---	protein binding
CG3528	22,5	7,3	8,4	-1,63	-1,43	0,20	4,18E-02	3,63E-01	---	protein binding
CG11905	25,4	14,8	14,3	-0,78	-0,82	-0,04	9,90E-03	4,71E-01	---	protein binding
CG32207	53,7	1,5	2,1	-5,19	-4,70	0,49	1,18E-03	3,73E-01	---	protein binding
CG14569	54,4	6,7	4,9	-3,02	-3,48	-0,46	6,79E-04	2,19E-01	---	protein binding
CG13512	86,9	36,1	33,5	-1,27	-1,38	-0,11	7,01E-03	3,82E-01	---	protein binding
CG13962	1554,0	49,1	230,1	-4,98	-2,76	2,23	5,21E-03	1,43E-02	---	protein binding
yellow-e2	26,0	18,4	12,0	-0,50	-1,12	-0,62	2,10E-02	5,75E-02	---	protein binding
CG12017	22,5	6,8	9,1	-1,72	-1,31	0,41	5,41E-02	3,46E-01	---	protein binding
Bestrophin 2	168,4	65,4	59,6	-1,36	-1,50	-0,13	8,56E-03	2,05E-01	---	protein binding
CG4880	133,2	125,4	64,4	-0,09	-1,05	-0,96	2,52E-02	3,63E-02	---	protein binding
CG9269	38,0	25,6	12,0	-0,57	-1,67	-1,10	1,19E-02	6,57E-02	---	protein binding
CG4022	63,1	34,9	31,3	-0,86	-1,01	-0,16	3,16E-03	1,96E-01	---	protein binding
CG11893	24,5	6,3	6,2	-1,95	-1,98	-0,03	3,65E-02	4,51E-01	---	protein binding
CG14696	67,0	35,2	24,3	-0,93	-1,46	-0,53	9,43E-03	2,28E-02	---	protein binding
CG13822	162,4	29,9	29,1	-2,44	-2,48	-0,04	2,77E-03	4,16E-01	---	protein binding
CG6310	120,6	43,5	43,8	-1,47	-1,46	0,01	8,79E-03	4,16E-01	---	protein binding
CG12521	34,4	20,1	14,6	-0,78	-1,24	-0,46	8,87E-02	3,33E-01	---	protein binding
CG9458	30,3	5,5	3,8	-2,45	-3,00	-0,54	5,61E-03	2,65E-01	---	protein binding
CG10918	72,4	19,9	17,1	-1,86	-2,08	-0,22	3,19E-03	1,73E-01	---	protein binding

**CG14598**	43,4	22,1	21,5	-0,97	-1,01	-0,04	3,93E-02	4,10E-01	---	protein binding
**CG10550**	32,5	31,1	15,9	-0,07	-1,03	-0,96	3,31E-03	6,86E-03	---	protein binding
**CG10208**	96,0	53,3	31,8	-0,85	-1,59	-0,74	1,92E-03	1,59E-02	---	protein binding
**CG14102**	21,8	13,2	9,2	-0,73	-1,25	-0,52	1,70E-02	3,12E-04	---	protein binding
**CG11882**	352,9	184,1	151,3	-0,94	-1,22	-0,28	9,46E-03	1,83E-01	---	protein binding /// identical protein binding
**ergic53**	517,6	365,1	247,3	-0,50	-1,07	-0,56	3,57E-03	4,60E-02	---	protein binding /// mannose binding
**CG32850**	62,8	36,7	6,9	-0,78	-3,19	-2,41	6,95E-03	2,05E-02	---	protein binding /// zinc ion binding /// metal ion binding
**CG17739**	115,3	32,1	39,5	-1,85	-1,55	0,30	1,36E-03	1,21E-03	---	serine-type endopeptidase inhibitor activity
**CG18349 /// CG7941**	28,4	1,6	1,2	-4,15	-4,56	-0,42	3,14E-03	3,18E-01	---	structural constituent of chitin-based cuticle /// protein binding /// structural constituent of chitin-based larval cuticle /// structural constituent of cuticle
**Larval cuticle protein 3**	198,2	26,9	53,0	-2,88	-1,90	0,98	7,88E-03	2,93E-01	---	structural constituent of chitin-based cuticle /// protein binding /// structural constituent of chitin-based larval cuticle /// structural constituent of cuticle
**CG18349**	42,7	8,2	10,5	-2,37	-2,02	0,35	2,95E-03	2,09E-01	---	structural constituent of chitin-based cuticle /// protein binding /// structural constituent of cuticle
**CG6131**	62,8	16,8	26,3	-1,90	-1,25	0,65	6,43E-04	3,65E-03	---	structural constituent of chitin-based cuticle /// structural constituent of cuticle
**CG15756**	432,2	219,6	175,9	-0,98	-1,30	-0,32	1,48E-03	1,39E-01	---	structural constituent of cuticle
**mitochondrial ribosomal protein L18 /// mRpL18**	102,3	72,7	42,7	-0,49	-1,26	-0,77	1,82E-02	1,82E-02	---	structural constituent of ribosome
**CG8209**	296,9	138,9	135,3	-1,10	-1,13	-0,04	2,12E-02	4,47E-01	---	zinc ion binding
**CG9119**	37,9	5,6	2,9	-2,77	-3,69	-0,92	3,24E-03	2,23E-03	---	zinc ion binding /// hydrolase activity, acting on ester bonds
**CG32335**	28,3	12,5	14,2	-1,18	-1,00	0,18	9,79E-02	3,49E-01	---	zinc ion binding /// hydrolase activity, acting on ester bonds
**CG9509**	299,8	811,6	58,0	1,44	-2,37	-3,81	1,51E-02	1,70E-03	alcohol metabolic process /// mesoderm development /// oxidation reduction	protein binding /// choline dehydrogenase activity /// choline dehydrogenase activity /// oxidoreductase activity /// oxidoreductase activity, acting on CH-OH group of donors /// FAD or FADH2 binding
**CG9521**	60,6	3,4	1,7	-4,14	-5,15	-1,01	9,80E-04	2,64E-01	alcohol metabolic process /// oxidation reduction	choline dehydrogenase activity /// choline dehydrogenase activity /// oxidoreductase activity /// oxidoreductase activity, acting on CH-OH group of donors /// FAD or FADH2 binding
**CG9512**	58,5	24,3	22,5	-1,27	-1,38	-0,11	7,08E-03	3,03E-01	alcohol metabolic process /// oxidation reduction	choline dehydrogenase activity /// choline dehydrogenase activity /// oxidoreductase activity /// oxidoreductase activity, acting on CH-OH group of donors /// FAD or FADH2 binding
**CG7888**	163,7	56,7	69,8	-1,53	-1,23	0,30	7,00E-03	1,22E-01	amino acid transmembrane transport	amino acid transmembrane transporter activity
**CG9510**	328,4	209,0	237,3	-0,65	-0,47	0,18	4,88E-02	3,33E-01	arginine biosynthetic process /// arginine biosynthetic process via ornithine	catalytic activity /// argininosuccinate lyase activity /// argininosuccinate lyase activity /// protein binding
**CG8745**	1452,9	164,7	231,3	-3,14	-2,65	0,49	1,63E-03	1,14E-01	arginine catabolic process to glutamate	catalytic activity /// ornithine-oxo-acid transaminase activity /// alanine-glyoxylate transaminase activity /// transaminase activity /// transferase activity /// pyridoxal phosphate binding
**CG11594**	102,4	59,0	50,7	-0,80	-1,01	-0,22	6,30E-03	2,03E-01	carbohydrate metabolic process	protein binding /// phosphotransferase activity, alcohol group as acceptor
**Chitinase 4**	101,9	33,4	23,9	-1,61	-2,09	-0,48	9,13E-03	7,75E-02	carbohydrate metabolic process /// chitin metabolic process /// chitin catabolic process /// cuticle chitin catabolic process /// metabolic process	catalytic activity /// hydrolase activity, hydrolyzing O-glycosyl compounds /// chitinase activity /// chitinase activity /// chitinase activity /// chitin binding /// hydrolase activity /// hydrolase activity, acting on glycosyl bonds /// cation binding
**CG18135**	855,2	413,5	321,4	-1,05	-1,41	-0,36	1,60E-02	7,63E-02	carbohydrate metabolic process /// glycerol metabolic process /// lipid metabolic process /// intracellular signaling pathway	catalytic activity /// phospholipase C activity /// protein binding /// phosphoric diester hydrolase activity /// glycerophosphodiester phosphodiesterase activity /// hydrolase activity /// carbohydrate binding /// phosphatidylcholine phospholipase C activity
**Ecdysone-inducible gene L3**	155,4	98,0	76,0	-0,67	-1,03	-0,37	2,06E-02	7,72E-02	carbohydrate metabolic process /// glycolysis /// glycolysis /// metabolic process /// cellular carbohydrate metabolic process /// oxidation reduction	catalytic activity /// L-lactate dehydrogenase activity /// L-lactate dehydrogenase activity /// L-lactate dehydrogenase activity /// L-lactate dehydrogenase activity /// binding /// protein binding /// oxidoreductase activity /// oxidoreductase activity, acting on the CH-OH group of donors, NAD or NADP as acceptor
**CG14935**	69,3	44,2	33,3	-0,65	-1,06	-0,41	7,25E-04	1,19E-01	carbohydrate metabolic process /// metabolic process	catalytic activity /// alpha-glucosidase activity /// alpha-glucosidase activity /// protein binding /// hydrolase activity /// hydrolase activity, acting on glycosyl bonds /// cation binding
**CG15117**	48,7	26,8	21,6	-0,86	-1,17	-0,31	1,96E-02	1,91E-01	carbohydrate metabolic process /// metabolic process	catalytic activity /// hydrolase activity, hydrolyzing O-glycosyl compounds /// beta-glucuronidase activity /// beta-glucuronidase activity /// hydrolase activity /// hydrolase activity, acting on glycosyl bonds /// cation binding
**CG12582**	133,2	50,1	64,9	-1,41	-1,04	0,37	3,92E-03	1,14E-02	carbohydrate metabolic process /// metabolic process	catalytic activity /// hydrolase activity, hydrolyzing O-glycosyl compounds /// beta-mannosidase activity /// beta-mannosidase activity /// hydrolase activity /// hydrolase activity, acting on glycosyl bonds /// cation binding
**Ect3**	59,1	14,9	3,2	-1,99	-4,22	-2,23	6,41E-03	1,20E-02	carbohydrate metabolic process /// metabolic process /// salivary gland cell autophagic cell death /// autophagic cell death	catalytic activity /// hydrolase activity, hydrolyzing O-glycosyl compounds /// beta-galactosidase activity /// beta-galactosidase activity /// hydrolase activity /// hydrolase activity, acting on glycosyl bonds /// cation binding
**CG11967**	22,5	9,9	9,5	-1,18	-1,24	-0,06	2,36E-02	4,26E-01	carbon utilization	carbonate dehydratase activity /// zinc ion binding /// lyase activity
**Neurotactin**	145,4	79,0	68,9	-0,88	-1,08	-0,20	3,47E-02	1,75E-02	cell adhesion /// axonogenesis /// axon guidance /// axonal fasciculation /// central nervous system development	---
**Cad74A**	117,0	22,0	24,8	-2,41	-2,24	0,17	1,88E-02	2,75E-01	cell adhesion /// homophilic cell adhesion /// calcium-dependent cell-cell adhesion /// dorsal appendage formation	calcium ion binding
**Neuroglian**	216,3	168,9	95,7	-0,36	-1,18	-0,82	4,44E-02	5,77E-02	cell adhesion /// neuron cell-cell adhesion /// neuron cell-cell adhesion /// neuron cell-cell adhesion /// epidermal growth factor receptor signaling pathway /// multicellular organismal development /// axonogenesis /// imaginal disc morphogenesis /// axon ensheathment /// mushroom body development /// mushroom body development /// septate junction assembly /// nerve maturation /// melanotic encapsulation of foreign target /// regulation of tube size, open tracheal system /// central complex development /// axon extension	calcium ion binding
**CG32302**	389,3	476,5	177,3	0,29	-1,13	-1,43	4,86E-02	3,30E-03	chitin metabolic process	chitin binding
**CG4778**	106,4	61,4	42,6	-0,79	-1,32	-0,53	8,23E-03	4,78E-02	chitin metabolic process	protein binding /// chitin binding /// chitin binding /// structural constituent of peritrophic membrane
**Hemolectin**	24,7	15,2	17,6	-0,70	-0,49	0,21	4,79E-03	3,59E-01	chitin metabolic process /// cell adhesion /// hemostasis /// hemostasis /// melanization defense response /// wound healing /// hemolymph coagulation	sugar binding /// chitin binding /// protein homodimerization activity
**His2B:CG17949 /// His2B:CG33910**	320,8	153,4	159,4	-1,06	-1,01	0,06	2,47E-02	4,08E-01	chromatin assembly or disassembly /// nucleosome assembly /// nucleosome assembly	DNA binding /// DNA binding /// DNA binding /// protein binding
**High mobility group protein D**	970,3	561,9	475,0	-0,79	-1,03	-0,24	3,07E-03	1,70E-01	chromatin organization /// muscle organ development /// dendrite morphogenesis	DNA binding /// AT DNA binding /// protein binding /// DNA bending activity /// DNA bending activity
**Ada2b**	75,8	17,4	17,7	-2,13	-2,10	0,03	5,08E-04	4,68E-01	chromatin remodeling /// transcription /// axon target recognition /// cell proliferation /// positive regulation of histone acetylation /// wing disc pattern formation /// eye pigment metabolic process /// histone H3 acetylation /// regulation of transcription /// regulation of transcription	DNA binding /// histone acetyltransferase activity /// protein binding /// zinc ion binding /// metal ion binding
**CG33324 /// grappa**	33,4	32,2	13,6	-0,05	-1,29	-1,24	1,41E-01	1,76E-02	chromatin silencing /// chromatin silencing /// chromatin silencing at telomere /// chromatin modification /// histone methylation /// chromatin silencing at centromere /// chromatin-mediated maintenance of transcription /// chromatin-mediated maintenance of transcription	methyltransferase activity /// transferase activity /// histone-lysine N-methyltransferase activity /// histone methyltransferase activity (H3-K79 specific)
**taranis**	230,4	122,9	102,5	-0,91	-1,17	-0,26	1,26E-02	7,42E-02	chromatin-mediated maintenance of transcription /// wing disc dorsal/ventral pattern formation	protein binding
**lethal (1) G0237**	84,8	31,5	34,5	-1,43	-1,30	0,13	1,36E-03	2,26E-01	chromosome segregation /// mitotic metaphase plate congression	---
**blistery**	30,5	23,5	13,0	-0,37	-1,23	-0,85	1,78E-02	7,49E-02	cytoskeletal anchoring at plasma membrane /// apposition of dorsal and ventral imaginal disc-derived wing surfaces /// imaginal disc-derived wing morphogenesis /// imaginal disc-derived wing morphogenesis	actin binding /// protein binding /// protein binding
**pyd3**	263,1	62,8	87,8	-2,07	-1,58	0,48	7,37E-04	8,52E-02	'de novo' pyrimidine base biosynthetic process /// pyrimidine base catabolic process /// nitrogen compound metabolic process	beta-ureidopropionase activity /// beta-ureidopropionase activity /// beta ureidopropionase activity /// beta-ureidopropionase activity /// hydrolase activity /// hydrolase activity, acting on carbon-nitrogen (but not peptide) bonds /// identical protein binding
**PGRP-SB1**	263,0	56,2	28,8	-2,23	-3,19	-0,97	4,17E-03	4,07E-02	defense response /// immune response /// peptidoglycan catabolic process /// peptidoglycan catabolic process /// innate immune response /// innate immune response	protein binding /// N-acetylmuramoyl-L-alanine amidase activity /// hydrolase activity /// peptidoglycan binding /// peptidoglycan binding /// metal ion binding
**PGRP-SB2**	3445,2	212,4	1013,0	-4,02	-1,77	2,25	2,03E-02	2,66E-02	defense response /// immune response /// peptidoglycan catabolic process /// peptidoglycan catabolic process /// innate immune response /// innate immune response	protein binding /// N-acetylmuramoyl-L-alanine amidase activity /// hydrolase activity /// peptidoglycan binding /// peptidoglycan binding /// metal ion binding
**Lysozyme X**	2487,3	1156,3	438,7	-1,11	-2,50	-1,40	4,55E-03	2,62E-02	defense response /// metabolic process /// cell wall macromolecule catabolic process /// antimicrobial humoral response /// cytolysis /// defense response to bacterium	lysozyme activity /// lysozyme activity /// lysozyme activity /// catalytic activity /// hydrolase activity /// hydrolase activity, acting on glycosyl bonds
**Deoxyribonuclease II**	279,2	117,7	100,2	-1,25	-1,48	-0,23	3,93E-04	4,41E-02	DNA metabolic process /// DNA catabolic process	deoxyribonuclease II activity /// deoxyribonuclease II activity /// deoxyribonuclease II activity /// deoxyribonuclease II activity /// protein binding /// hydrolase activity
**male-specific lethal 2**	85,4	59,0	40,8	-0,53	-1,07	-0,53	2,86E-02	1,08E-01	dosage compensation /// dosage compensation, by hyperactivation of X chromosome /// dosage compensation complex assembly involved in dosage compensation by hyperactivation of X chromosome	DNA binding /// DNA binding /// chromatin binding /// chromatin binding /// protein binding /// protein binding /// zinc ion binding /// metal ion binding
**CG40123**	312,3	168,1	159,8	-0,89	-0,97	-0,07	5,97E-03	3,43E-01	ecdysone biosynthetic process /// molting cycle, chitin-based cuticle /// oxidation reduction	monooxygenase activity /// iron ion binding /// electron carrier activity /// oxidoreductase activity /// heme binding /// metal ion binding

mirror	37,5	15,2	15,7	-1,31	-1,26	0,05	4,63E-02	4,70E-01	embryonic development via the syncytial blastoderm /// embryonic development via the syncytial blastoderm /// compound eye morphogenesis /// regulation of transcription, DNA-dependent /// regulation of transcription, DNA-dependent /// smoothened signaling pathway /// peripheral nervous system development /// imaginal disc-derived wing morphogenesis /// equator specification /// equator specification /// regulation of transcription /// positive regulation of transcription /// dorsal appendage formation	DNA binding /// sequence-specific DNA binding transcription factor activity /// sequence-specific DNA binding transcription factor activity /// sequence-specific DNA binding transcription factor activity /// protein binding /// transcription activator activity /// transcription regulator activity /// sequence-specific DNA binding
CG31431	36,6	11,9	9,8	-1,62	-1,90	-0,28	2,51E-02	4,12E-01	fibroblast growth factor receptor signaling pathway	fibroblast growth factor receptor activity
Cyclin B	268,3	137,1	111,9	-0,97	-1,26	-0,29	2,48E-03	6,17E-02	G2/M transition of mitotic cell cycle /// mitotic anaphase B /// cytokinesis after mitosis /// cytokinesis /// embryonic development via the syncytial blastoderm /// response to DNA damage stimulus /// cell cycle /// mitosis /// mitotic chromosome movement towards spindle pole /// attachment of spindle microtubules to kinetochore /// attachment of spindle microtubules to kinetochore /// cellular process /// syncytial blastoderm mitotic cell cycle /// cell division	protein binding /// cyclin-dependent protein kinase regulator activity /// cyclin-dependent protein kinase regulator activity /// cyclin-dependent protein kinase regulator activity
lethal (2) k05713	61,5	29,6	32,0	-1,06	-0,94	0,11	1,85E-02	7,41E-02	glycerol metabolic process /// glycerol-3-phosphate metabolic process /// glycerophosphate shuttle /// flight behavior /// oxidation reduction	glycerol-3-phosphate dehydrogenase activity /// glycerol-3-phosphate dehydrogenase activity /// glycerol-3-phosphate dehydrogenase activity /// calcium ion binding /// oxidoreductase activity
delilah	21,9	10,8	10,0	-1,03	-1,13	-0,10	5,42E-02	1,90E-01	glycolysis /// transcription /// multicellular organismal development /// mesoderm development /// somatic muscle development /// metabolic process /// muscle attachment /// regulation of transcription	DNA binding /// sequence-specific DNA binding transcription factor activity /// specific RNA polymerase II transcription factor activity /// catalytic activity /// fructose-bisphosphate aldolase activity /// protein binding /// lyase activity /// transcription regulator activity
myoglianin	37,6	45,7	17,0	0,28	-1,15	-1,43	1,44E-02	8,63E-02	growth	growth factor activity /// growth factor activity
escl	55,0	44,3	21,0	-0,31	-1,39	-1,07	4,02E-03	2,09E-02	histone H3-K27 methylation	protein binding /// histone methyltransferase activity
CG4616	20,7	10,7	10,0	-0,95	-1,04	-0,10	1,37E-02	4,49E-01	histone mRNA 3'-end processing	---
Peroxiredoxin 2540	29,4	7,1	5,3	-2,05	-2,46	-0,41	8,03E-03	5,74E-02	hydrogen peroxide catabolic process /// cell redox homeostasis /// oxidation reduction	peroxidase activity /// peroxidase activity /// peroxidase activity /// glutathione peroxidase activity /// glutathione peroxidase activity /// thioredoxin peroxidase activity /// antioxidant activity /// antioxidant activity /// antioxidant activity /// oxidoreductase activity
CG40050	70,8	30,4	26,6	-1,22	-1,41	-0,19	2,92E-02	1,89E-01	instar larval development /// metamorphosis /// multicellular organism growth /// ecdysteroid biosynthetic process /// oxidation reduction	oxidoreductase activity /// metal ion binding /// iron-sulfur cluster binding /// 2 iron, 2 sulfur cluster binding
CG32541	81,1	43,9	50,6	-0,89	-0,68	0,20	7,27E-02	3,47E-01	inter-male aggressive behavior	---
sugarless	1353,4	614,0	519,7	-1,14	-1,38	-0,24	3,25E-04	2,62E-02	inter-male aggressive behavior /// glycosaminoglycan biosynthetic process /// cell surface receptor linked signaling pathway /// smoothened signaling pathway /// segment polarity determination /// epithelial cell migration, open tracheal system /// epithelial cell migration, open tracheal system /// epithelial cell migration, open tracheal system /// primary branching, open tracheal system /// heart development /// mesoderm migration involved in gastrulation /// mesoderm migration involved in gastrulation	catalytic activity /// UDP-glucose 6-dehydrogenase activity /// UDP-glucose 6-dehydrogenase activity /// UDP-glucose 6-dehydrogenase activity /// binding /// oxidoreductase activity /// oxidoreductase activity, acting on the CH-OH group of donors, NAD or NADP as acceptor /// NAD or NADH binding
Transferrin 3	165,4	43,9	41,1	-1,91	-2,01	-0,10	1,44E-02	3,74E-01	iron ion transport /// cellular iron ion homeostasis /// response to bacterium	iron ion transmembrane transporter activity /// ferric iron binding
Juvenile hormone epoxide hydrolase 3	45,5	24,1	24,1	-0,92	-0,91	0,00	2,47E-03	4,94E-01	juvenile hormone catabolic process /// response to toxin	catalytic activity /// epoxide hydrolase activity /// epoxide hydrolase activity /// juvenile hormone epoxide hydrolase activity /// hydrolase activity
Lcp65Ab2	58,1	4,9	18,0	-3,56	-1,69	1,86	3,20E-03	1,57E-01	larval chitin-based cuticle development	structural constituent of chitin-based cuticle /// structural constituent of chitin-based larval cuticle /// structural constituent of chitin-based larval cuticle /// structural constituent of cuticle
Hand	77,9	68,8	37,2	-0,18	-1,07	-0,89	2,34E-02	4,57E-02	larval heart development /// hemopoiesis /// embryonic heart tube development /// negative regulation of apoptosis /// regulation of transcription /// regulation of transcription	sequence-specific DNA binding transcription factor activity /// transcription activator activity /// transcription regulator activity
pastrel /// pst	1373,6	577,2	340,9	-1,25	-2,01	-0,76	5,29E-03	2,62E-02	learning or memory /// olfactory learning	---
CG10399	52,4	39,3	19,2	-0,42	-1,45	-1,03	2,32E-03	6,27E-03	leucine metabolic process /// metabolic process	catalytic activity /// hydroxymethylglutaryl-CoA lyase activity /// hydroxymethylglutaryl-CoA lyase activity /// lyase activity
CG10116	35,8	31,4	16,6	-0,19	-1,10	-0,92	1,15E-02	9,89E-05	lipid metabolic process	catalytic activity /// lipoprotein lipase activity /// lipoprotein lipase activity /// hydrolase activity
CG9743	79,3	21,6	19,5	-1,88	-2,02	-0,15	8,28E-03	4,04E-01	lipid metabolic process /// fatty acid biosynthetic process /// lipid biosynthetic process /// oxidation reduction	stearoyl-CoA 9-desaturase activity /// stearoyl-CoA 9-desaturase activity /// oxidoreductase activity /// oxidoreductase activity, acting on paired donors, with oxidation of a pair of donors resulting in the reduction of molecular oxygen to two molecules of water
Cytochrome b5-related	80,7	40,5	39,7	-1,00	-1,02	-0,03	1,24E-02	4,82E-01	lipid metabolic process /// fatty acid biosynthetic process /// oxidation reduction	iron ion binding /// electron carrier activity /// oxidoreductase activity /// oxidoreductase activity, acting on paired donors, with oxidation of a pair of donors resulting in the reduction of molecular oxygen to two molecules of water /// heme binding /// metal ion binding
Yolk protein 3	39,8	5,5	9,0	-2,87	-2,15	0,72	1,61E-02	4,85E-02	lipid metabolic process /// vitellogenesis /// vitellogenesis /// sex differentiation /// embryo development	catalytic activity /// structural molecule activity
CG7003	61,2	26,2	27,5	-1,22	-1,15	0,07	2,69E-03	3,71E-01	meiotic mismatch repair /// DNA repair /// DNA repair /// mismatch repair /// postreplication repair /// response to DNA damage stimulus	nucleotide binding /// DNA binding /// ATP binding /// mismatched DNA binding
hu li tai shao	258,9	161,8	129,1	-0,68	-1,00	-0,33	2,25E-02	1,42E-01	meiotic spindle organization /// multicellular organismal development /// cystoblast division /// germarium-derived oocyte fate determination /// female germline ring canal formation, actin assembly /// female germline ring canal formation, actin assembly /// female germline ring canal formation, actin assembly /// cell differentiation /// spectrosome organization /// ovarian fusome organization	actin binding /// actin binding /// protein binding /// metal ion binding
CG11314	984,7	473,4	463,4	-1,06	-1,09	-0,03	1,22E-03	4,53E-01	mesoderm development	---
Glycogenin	234,0	112,4	85,2	-1,06	-1,46	-0,40	4,78E-02	1,92E-01	mesoderm development	protein binding /// glycogenin glucosyltransferase activity /// glycogenin glucosyltransferase activity /// transferase activity, transferring glycosyl groups
CG12896 /// Peroxiredoxin 2540 /// Peroxiredoxin 2540	330,7	92,4	100,5	-1,84	-1,72	0,12	5,46E-02	3,73E-01	mesoderm development /// hydrogen peroxide catabolic process /// cell redox homeostasis /// oxidation reduction	peroxidase activity /// peroxidase activity /// peroxidase activity /// glutathione peroxidase activity /// glutathione peroxidase activity /// thioredoxin peroxidase activity /// antioxidant activity /// antioxidant activity /// antioxidant activity /// oxidoreductase activity /// peroxiredoxin activity
goliath	95,7	51,0	38,9	-0,91	-1,30	-0,39	2,45E-04	5,95E-03	mesoderm formation /// transcription /// regulation of transcription, DNA-dependent /// multicellular organismal development /// mesoderm development /// regulation of transcription	DNA binding /// protein binding /// zinc ion binding /// metal ion binding
CG3292	40,8	22,0	12,7	-0,89	-1,69	-0,79	2,34E-02	5,29E-03	metabolic process	catalytic activity /// alkaline phosphatase activity /// alkaline phosphatase activity /// hydrolase activity /// phosphatase activity
CG17562	70,6	48,7	29,9	-0,54	-1,24	-0,70	3,03E-03	4,76E-02	metabolic process	catalytic activity /// binding
CG4872	70,6	43,1	29,1	-0,71	-1,28	-0,57	1,46E-02	4,89E-02	metabolic process	catalytic activity /// binding
CG4594	72,3	47,9	33,9	-0,59	-1,09	-0,50	9,07E-03	2,95E-02	metabolic process	catalytic activity /// dodecenoyl-CoA delta-isomerase activity /// dodecenoyl-CoA delta-isomerase activity /// isomerase activity
CG17681	27,0	11,6	12,4	-1,22	-1,12	0,10	1,93E-02	3,94E-01	metabolic process	N-acetyltransferase activity /// acyltransferase activity /// transferase activity
CG7798	22,1	6,1	6,1	-1,85	-1,85	0,00	1,82E-02	5,00E-01	metabolic process /// cell wall macromolecule catabolic process /// antimicrobial humoral response	lysozyme activity /// lysozyme activity /// lysozyme activity /// hydrolase activity /// hydrolase activity, acting on glycosyl bonds
Lysozyme S	236,4	187,3	59,4	-0,34	-1,99	-1,66	6,50E-03	6,93E-02	metabolic process /// cell wall macromolecule catabolic process /// antimicrobial humoral response /// cytolysis /// defense response to bacterium	lysozyme activity /// lysozyme activity /// lysozyme activity /// catalytic activity /// hydrolase activity /// hydrolase activity, acting on glycosyl bonds
CG4842	54,8	29,0	21,5	-0,92	-1,35	-0,44	3,65E-02	5,70E-02	metabolic process /// oxidation reduction	catalytic activity /// alcohol dehydrogenase (NAD) activity /// binding /// oxidoreductase activity
CG2254	39,7	28,1	16,2	-0,50	-1,29	-0,80	1,89E-02	7,56E-02	metabolic process /// oxidation reduction	catalytic activity /// binding /// oxidoreductase activity
CG3699	100,9	29,1	36,2	-1,80	-1,48	0,32	4,12E-03	8,05E-02	metabolic process /// oxidation reduction	catalytic activity /// binding /// oxidoreductase activity /// oxidoreductase activity, acting on CH-OH group of donors
CG7675	414,3	184,3	195,1	-1,17	-1,09	0,08	1,07E-02	3,19E-01	metabolic process /// oxidation reduction	catalytic activity /// binding /// protein binding /// oxidoreductase activity /// oxidoreductase activity, acting on CH-OH group of donors
CG31810	36,7	14,9	14,3	-1,31	-1,36	-0,06	1,19E-03	4,42E-01	metabolic process /// steroid metabolic process /// oxidation reduction	catalytic activity /// binding /// steroid dehydrogenase activity /// oxidoreductase activity
SP2523	121,8	80,3	59,7	-0,60	-1,03	-0,43	3,99E-02	8,58E-02	microtubule cytoskeleton organization /// signal transduction /// neuromuscular junction development /// axon extension	protein binding
Tubulin at 97EF	122,1	71,6	55,2	-0,77	-1,14	-0,38	3,77E-03	2,25E-01	microtubule-based process /// microtubule-based movement /// protein polymerization	nucleotide binding /// GTPase activity /// structural molecule activity /// structural constituent of cytoskeleton /// GTP binding
CG10648	38,5	30,4	14,3	-0,34	-1,42	-1,08	3,78E-02	3,63E-02	mitotic spindle elongation /// translation	structural constituent of ribosome
CG4865	37,4	28,8	15,9	-0,38	-1,23	-0,85	1,37E-03	7,54E-02	mitotic spindle organization /// regulation of mitosis	protein binding
Muscle LIM protein at 60A	402,0	199,9	156,4	-1,01	-1,36	-0,35	7,98E-03	6,08E-02	multicellular organismal development /// muscle organ development /// regulation of striated muscle tissue development /// cell differentiation	protein binding /// zinc ion binding /// zinc ion binding /// metal ion binding
CG7447	803,8	358,5	581,1	-1,16	-0,47	0,70	3,72E-02	1,32E-02	muscle attachment	calcium ion binding /// protein binding
Myosin alkali light chain 1	253,5	140,5	114,9	-0,85	-1,14	-0,29	1,92E-02	1,64E-01	muscle contraction /// mesoderm development	microfilament motor activity /// motor activity /// calcium ion binding /// calcium ion binding /// ATPase activity, coupled
CG4091	528,3	333,2	211,5	-0,67	-1,32	-0,66	6,53E-05	1,70E-02	negative regulation of anti-apoptosis /// salivary gland cell autophagic cell death /// autophagic cell death	caspase inhibitor activity
Kruppel	180,4	106,3	82,5	-0,76	-1,13	-0,36	1,88E-02	1,63E-01	negative regulation of transcription from RNA polymerase II promoter /// chromatin silencing /// multicellular organismal development /// zygotic determination of anterior/posterior axis, embryo /// zygotic determination of anterior/posterior axis, embryo /// neuroblast fate determination /// neuroblast fate determination /// ganglion mother cell fate determination /// axon guidance /// ventral cord development /// Malpighian tubule morphogenesis /// Malpighian tubule morphogenesis /// muscle organ development	nucleic acid binding /// DNA binding /// DNA binding /// chromatin binding /// specific RNA polymerase II transcription factor activity /// protein binding /// zinc ion binding /// transcription repressor activity /// specific transcriptional repressor activity /// specific transcriptional repressor activity /// metal ion binding

**dorsal**	37,7	20,1	21,4	-0,91	-0,81	0,10	1,16E-02	3,83E-01	negative regulation of transcription from RNA polymerase II promoter /// regulation of alternative nuclear mRNA splicing, via spliceosome /// ectodermal cell fate specification /// defense response /// immune response /// immune response /// immune response /// multicellular organismal development /// gastrulation /// ectoderm development /// ventral cord development /// mesoderm development /// mesodermal cell fate specification /// heart development /// Toll signaling pathway	DNA binding /// DNA binding /// sequence-specific DNA binding transcription factor activity /// sequence-specific DNA binding transcription factor activity /// sequence-specific DNA binding transcription factor activity /// RNA polymerase II transcription factor activity /// protein binding /// morphogen activity /// transcription activator activity /// transcription repressor activity /// sequence-specific DNA binding /// high mobility group box 1 binding /// transcription repressor binding
**CG7344 /// dystrophin**	23,1	13,4	9,7	-0,78	-1,26	-0,47	3,01E-02	1,24E-01	neuromuscular synaptic transmission /// imaginal disc-derived wing vein specification /// muscle organ development /// muscle organ development /// imaginal disc-derived wing vein morphogenesis /// establishment of cell polarity /// muscle cell homeostasis /// regulation of neurotransmitter secretion /// regulation of neurotransmitter secretion /// regulation of short-term neuronal synaptic plasticity	actin binding /// actin binding /// structural molecule activity /// structural constituent of cytoskeleton /// calcium ion binding /// protein binding /// protein binding /// cytoskeletal protein binding /// zinc ion binding /// structural constituent of muscle /// metal ion binding /// WW domain binding
**CG9344**	299,7	104,9	101,2	-1,51	-1,57	-0,05	1,21E-02	3,80E-01	nuclear mRNA splicing, via spliceosome	protein binding
**hephaestus**	313,2	214,7	155,9	-0,54	-1,01	-0,46	2,23E-02	1,37E-01	nuclear mRNA splicing, via spliceosome /// mRNA processing /// Notch signaling pathway /// spermatid development /// negative regulation of oskar mRNA translation /// oocyte microtubule cytoskeleton polarization /// imaginal disc-derived wing vein morphogenesis /// imaginal disc-derived wing margin morphogenesis	nucleotide binding /// translation repressor activity, nucleic acid binding /// nucleic acid binding /// RNA binding /// mRNA binding /// mRNA 3'-UTR binding /// protein binding /// poly-pyrimidine tract binding /// poly-pyrimidine tract binding
**CG11883**	140,3	78,9	72,1	-0,83	-0,96	-0,13	1,24E-02	2,79E-01	nucleotide catabolic process	5'-nucleotidase activity /// 5'-nucleotidase activity /// hydrolase activity
**CG30104**	330,8	54,3	64,4	-2,61	-2,36	0,25	5,94E-05	4,33E-02	nucleotide catabolic process	nucleotide binding /// hydrolase activity /// hydrolase activity, acting on ester bonds /// metal ion binding
**Pray For Elves**	1,7	4,2	2,6	1,27	0,57	-0,70	2,77E-01	7,63E-03	ocellus development	protein binding
**CG5321**	23,5	7,5	2,3	-1,65	-3,37	-1,72	1,55E-02	1,87E-01	oxidation reduction	gamma-butyrobetaine dioxygenase activity /// gamma-butyrobetaine dioxygenase activity /// oxidoreductase activity
**Cyp12c1**	337,5	14,8	10,5	-4,51	-5,01	-0,50	5,41E-03	5,79E-03	oxidation reduction	monooxygenase activity /// iron ion binding /// electron carrier activity /// electron carrier activity /// electron carrier activity /// oxidoreductase activity /// heme binding /// metal ion binding
**Cyp12e1**	141,4	92,2	63,8	-0,62	-1,15	-0,53	6,35E-03	1,63E-01	oxidation reduction	monooxygenase activity /// iron ion binding /// electron carrier activity /// electron carrier activity /// electron carrier activity /// oxidoreductase activity /// heme binding /// metal ion binding
**Cyp309a1**	161,4	111,2	57,3	-0,54	-1,49	-0,96	1,94E-02	4,58E-02	oxidation reduction	monooxygenase activity /// iron ion binding /// electron carrier activity /// electron carrier activity /// electron carrier activity /// oxidoreductase activity /// heme binding /// metal ion binding
**Cytochrome P450-4d1**	595,0	393,7	293,7	-0,60	-1,02	-0,42	4,36E-03	4,94E-02	oxidation reduction	monooxygenase activity /// iron ion binding /// electron carrier activity /// electron carrier activity /// electron carrier activity /// oxidoreductase activity /// heme binding /// metal ion binding
**Cytochrome P450-4p1**	75,3	29,3	7,2	-1,36	-3,39	-2,03	6,38E-03	1,06E-02	oxidation reduction	monooxygenase activity /// iron ion binding /// protein binding /// electron carrier activity /// electron carrier activity /// electron carrier activity /// oxidoreductase activity /// heme binding /// metal ion binding
**CG12224**	71,9	21,7	15,5	-1,73	-2,22	-0,49	3,32E-03	1,36E-01	oxidation reduction	oxidoreductase activity /// oxidoreductase activity
**CG14882**	27,3	17,5	12,0	-0,65	-1,19	-0,55	7,19E-02	1,36E-01	oxidation reduction	protein binding /// oxidoreductase activity /// [methionine synthase] reductase activity /// [methionine synthase] reductase activity
**Cyp12d1-p /// Cyp12d1-d**	271,3	20,6	128,5	-3,72	-1,08	2,64	8,35E-03	2,27E-02	oxidation reduction /// oxidation reduction	monooxygenase activity /// iron ion binding /// electron carrier activity /// electron carrier activity /// electron carrier activity /// oxidoreductase activity /// heme binding /// 1-alpha,25-dihydroxyvitamin D3 24-hydroxylase activity /// metal ion binding
**Cyp12d1-d**	23,1	3,8	11,3	-2,61	-1,03	1,59	6,90E-03	2,90E-03	oxidation reduction /// oxidation reduction	monooxygenase activity /// iron ion binding /// electron carrier activity /// electron carrier activity /// electron carrier activity /// oxidoreductase activity /// heme binding /// 1-alpha,25-dihydroxyvitamin D3 24-hydroxylase activity /// metal ion binding
**prolyl-4-hydroxylase-alpha MP**	28,1	13,4	17,2	-1,07	-0,71	0,36	4,79E-02	1,06E-01	peptidyl-proline hydroxylation to 4-hydroxy-L-proline /// oxidation reduction	procollagen-proline 4-dioxygenase activity /// procollagen-proline 4-dioxygenase activity /// procollagen-proline 4-dioxygenase activity /// binding /// iron ion binding /// oxidoreductase activity /// oxidoreductase activity, acting on single donors with incorporation of molecular oxygen, incorporation of two atoms of oxygen /// oxidoreductase activity, acting on paired donors, with incorporation or reduction of molecular oxygen /// L-ascorbic acid binding
**Eip93F**	102,1	51,4	50,8	-0,99	-1,01	-0,02	6,56E-02	4,84E-01	phagocytosis, engulfment /// autophagy /// activation of caspase activity /// induction of apoptosis by hormones /// larval midgut histolysis /// salivary gland cell autophagic cell death /// salivary gland cell autophagic cell death /// ecdysone-mediated induction of salivary gland cell autophagic cell death /// regulation of transcription /// positive regulation of transcription, DNA-dependent /// autophagic cell death /// autophagic cell death	DNA binding /// DNA binding /// sequence-specific DNA binding transcription factor activity
**CG5991**	213,6	96,0	99,0	-1,15	-1,11	0,04	2,36E-03	2,27E-01	phospholipid biosynthetic process	phosphatidylserine decarboxylase activity /// phosphatidylserine decarboxylase activity /// lyase activity
**secretory Phospholipase A2**	37,4	21,2	16,0	-0,82	-1,22	-0,40	2,84E-02	1,09E-01	phospholipid metabolic process /// lipid catabolic process	phospholipase A2 activity /// phospholipase A2 activity /// phospholipase A2 activity /// calcium ion binding /// calcium-dependent phospholipase A2 activity
**Ornithine decarboxylase 2**	21,1	8,7	6,5	-1,28	-1,70	-0,42	1,53E-03	1,22E-01	polyamine biosynthetic process /// polyamine biosynthetic process	catalytic activity /// ornithine decarboxylase activity /// ornithine decarboxylase activity /// protein binding /// lyase activity /// carboxy-lyase activity
**CG10830**	20,0	9,2	9,0	-1,12	-1,15	-0,03	1,19E-02	4,67E-01	potassium ion transport	voltage-gated potassium channel activity /// protein binding
**CG5096 /// CG5091**	109,8	75,3	37,2	-0,55	-1,56	-1,02	6,61E-03	2,85E-02	protein N-linked glycosylation	protein binding /// protein binding /// transferase activity /// transferase activity, transferring glycosyl groups /// transferase activity, transferring hexosyl groups /// glucosyltransferase activity
**CG7156**	53,6	32,9	25,4	-0,70	-1,08	-0,37	1,15E-02	6,96E-02	protein phosphorylation /// cell communication	protein kinase activity /// protein kinase activity /// protein binding /// protein binding /// ATP binding /// phosphoinositide binding
**Pak3**	425,5	99,2	78,7	-2,10	-2,43	-0,33	5,58E-03	6,77E-02	protein phosphorylation /// protein phosphorylation /// actin filament organization	nucleotide binding /// protein kinase activity /// protein serine/threonine kinase activity /// receptor signaling protein serine/threonine kinase activity /// ATP binding /// kinase activity /// transferase activity
**p38c**	29,9	24,4	12,9	-0,29	-1,21	-0,92	1,12E-02	2,13E-03	protein phosphorylation /// protein phosphorylation /// signal transduction	nucleotide binding /// protein kinase activity /// protein serine/threonine kinase activity /// MAP kinase activity /// MAP kinase activity /// ATP binding /// kinase activity /// transferase activity
**CG8539**	48,2	33,6	21,9	-0,52	-1,14	-0,62	1,70E-02	1,35E-01	proteolysis	carboxypeptidase activity /// metallocarboxypeptidase activity /// metallocarboxypeptidase activity /// protein binding /// zinc ion binding /// hydrolase activity
**CG18585**	36,3	39,6	13,5	0,13	-1,43	-1,55	3,11E-04	3,68E-03	proteolysis	carboxypeptidase activity /// metallocarboxypeptidase activity /// zinc ion binding /// hydrolase activity
**Sarcoplasmic calcium-binding protein 2**	277,9	148,4	103,7	-0,90	-1,42	-0,52	6,93E-03	1,49E-01	proteolysis	catalytic activity /// GTPase activity /// GTPase activity /// endopeptidase activity /// serine-type endopeptidase activity /// calcium ion binding /// calcium ion binding /// calcium ion binding /// protein binding
**CG33465**	68,3	50,9	32,5	-0,42	-1,07	-0,65	4,77E-02	1,70E-01	proteolysis	catalytic activity /// serine-type endopeptidase activity
**CG30288**	24,3	15,3	13,7	-0,67	-0,83	-0,16	5,90E-02	4,01E-01	proteolysis	catalytic activity /// serine-type endopeptidase activity /// hydrolase activity
**CG30286**	22,9	6,8	2,9	-1,76	-3,00	-1,24	3,49E-03	3,00E-02	proteolysis	catalytic activity /// serine-type endopeptidase activity /// hydrolase activity
**CG31205**	57,4	13,3	24,7	-2,11	-1,22	0,90	3,99E-02	9,14E-03	proteolysis	catalytic activity /// serine-type endopeptidase activity /// hydrolase activity
**CG30287**	40,0	25,6	16,0	-0,65	-1,33	-0,68	3,06E-02	5,57E-03	proteolysis	catalytic activity /// serine-type endopeptidase activity /// peptidase activity /// serine-type peptidase activity /// hydrolase activity
**CG30083**	166,6	11,8	33,7	-3,82	-2,31	1,52	5,89E-03	5,06E-02	proteolysis	catalytic activity /// serine-type endopeptidase activity /// peptidase activity /// serine-type peptidase activity /// hydrolase activity
**CG30090**	86,2	31,4	28,6	-1,45	-1,59	-0,13	6,72E-02	3,80E-01	proteolysis	catalytic activity /// serine-type endopeptidase activity /// peptidase activity /// serine-type peptidase activity /// hydrolase activity
**CG3117**	67,1	42,7	27,6	-0,65	-1,28	-0,63	2,24E-02	2,31E-02	proteolysis	catalytic activity /// serine-type endopeptidase activity /// serine-type endopeptidase activity /// hydrolase activity
**CG9672**	269,5	203,8	43,1	-0,40	-2,64	-2,24	8,57E-03	2,19E-02	proteolysis	catalytic activity /// serine-type endopeptidase activity /// serine-type endopeptidase activity /// hydrolase activity
**CG32412**	210,3	99,2	79,6	-1,08	-1,40	-0,32	5,30E-04	1,15E-01	proteolysis	peptidase activity /// acyltransferase activity /// glutaminyl-peptide cyclotransferase activity /// glutaminyl-peptide cyclotransferase activity /// glutaminyl-peptide cyclotransferase activity /// transferase activity /// hydrolase activity
**CG14642**	211,7	63,1	66,9	-1,75	-1,66	0,08	2,53E-03	2,84E-01	proteolysis /// proteolysis	catalytic activity /// serine-type endopeptidase activity /// serine-type endopeptidase activity /// peptidase activity /// serine-type peptidase activity /// hydrolase activity
**CG11192**	149,4	10,1	20,3	-3,89	-2,88	1,01	2,70E-02	1,31E-01	proteolysis /// proteolysis	catalytic activity /// serine-type endopeptidase activity /// serine-type endopeptidase activity /// peptidase activity /// serine-type peptidase activity /// hydrolase activity
**CG18179**	707,7	27,3	44,6	-4,70	-3,99	0,71	1,52E-02	3,00E-01	proteolysis /// proteolysis	catalytic activity /// serine-type endopeptidase activity /// serine-type endopeptidase activity /// peptidase activity /// serine-type peptidase activity /// hydrolase activity
**CG10472**	95,7	46,0	30,8	-1,06	-1,64	-0,58	3,58E-03	7,44E-02	proteolysis /// proteolysis	catalytic activity /// serine-type endopeptidase activity /// serine-type endopeptidase activity /// protein binding /// peptidase activity /// serine-type peptidase activity /// hydrolase activity
**Jonah 74E**	1119,8	37,8	33,9	-4,89	-5,04	-0,16	2,51E-03	2,66E-01	proteolysis /// proteolysis	catalytic activity /// serine-type endopeptidase activity /// serine-type endopeptidase activity /// serine-type endopeptidase activity /// peptidase activity /// serine-type peptidase activity /// hydrolase activity

**CG10764**	405,0	214,7	192,9	-0,92	-1,07	-0,15	1,33E-02	2,86E-01	proteolysis /// proteolysis	catalytic activity /// serine-type endopeptidase activity /// serine-type endopeptidase activity /// serine-type endopeptidase activity /// peptidase activity /// serine-type peptidase activity /// hydrolase activity
**CG8329**	281,4	9,7	23,3	-4,86	-3,59	1,27	2,16E-02	9,28E-02	proteolysis /// proteolysis	catalytic activity /// serine-type endopeptidase activity /// serine-type endopeptidase activity /// serine-type endopeptidase activity /// peptidase activity /// serine-type peptidase activity /// hydrolase activity
**Jonah 25Bii**	518,7	346,5	228,2	-0,58	-1,18	-0,60	2,60E-02	1,56E-01	proteolysis /// proteolysis	catalytic activity /// serine-type endopeptidase activity /// serine-type endopeptidase activity /// serine-type endopeptidase activity /// protein binding /// peptidase activity /// serine-type peptidase activity /// hydrolase activity
**CG18477 /// CG31780**	389,8	257,8	105,3	-0,60	-1,89	-1,29	1,99E-03	5,43E-02	proteolysis /// proteolysis	catalytic activity /// serine-type endopeptidase activity /// serine-type endopeptidase activity /// serine-type peptidase activity /// hydrolase activity
**persephone**	31,0	41,8	13,1	0,43	-1,25	-1,68	1,02E-02	1,18E-02	proteolysis /// proteolysis /// proteolysis /// proteolysis /// defense response /// positive regulation of antifungal peptide biosynthetic process /// regulation of Toll signaling pathway /// innate immune response /// innate immune response /// defense response to fungus /// defense response to fungus	catalytic activity /// serine-type endopeptidase activity /// serine-type endopeptidase activity /// peptidase activity /// peptidase activity /// peptidase activity /// serine-type peptidase activity /// hydrolase activity
**CG10932**	283,3	141,5	104,0	-1,00	-1,45	-0,44	1,46E-04	5,05E-04	pyruvate metabolic process /// fatty acid biosynthetic process /// mitotic spindle organization /// metabolic process	catalytic activity /// acetyl-CoA C-acetyltransferase activity /// acetyl-CoA C-acetyltransferase activity /// acyltransferase activity /// transferase activity /// transferase activity, transferring acyl groups other than amino-acyl groups
**CG1894**	41,3	2,4	3,8	-4,08	-3,44	0,64	1,44E-02	3,53E-01	regulation of transcription	histone acetyltransferase activity /// histone acetyltransferase activity /// protein binding /// acyltransferase activity /// transferase activity /// transferase activity, transferring acyl groups other than amino-acyl groups
**extra-extra**	31,3	23,6	14,4	-0,40	-1,12	-0,71	1,36E-01	2,04E-01	regulation of transcription, DNA-dependent /// nervous system development /// axon target recognition /// central nervous system development /// regulation of transcription /// regulation of transcription	DNA binding /// sequence-specific DNA binding transcription factor activity /// sequence-specific DNA binding transcription factor activity /// transcription regulator activity /// sequence-specific DNA binding
**reversed polarity**	62,9	30,6	26,7	-1,04	-1,24	-0,20	6,44E-03	2,48E-01	regulation of transcription, DNA-dependent /// regulation of transcription from RNA polymerase II promoter /// glial cell differentiation /// regulation of transcription	DNA binding /// sequence-specific DNA binding transcription factor activity /// RNA polymerase II transcription factor activity /// transcription regulator activity /// sequence-specific DNA binding
**tailup**	48,0	28,8	23,8	-0,74	-1,01	-0,27	2,01E-03	1,55E-01	regulation of transcription, DNA-dependent /// regulation of transcription from RNA polymerase II promoter /// terminal region determination /// germ-band shortening /// germ-band shortening /// dorsal closure /// dorsal closure /// nervous system development /// motor axon guidance /// head involution /// torso signaling pathway /// bristle morphogenesis /// citrate transport /// notum cell fate specification /// regulation of transcription /// amnioserosa	DNA binding /// sequence-specific DNA binding transcription factor activity /// specific RNA polymerase II transcription factor activity /// protein binding /// zinc ion binding /// citrate transmembrane transporter activity /// transcription regulator activity /// sequence-specific DNA binding /// metal ion binding
**Heat-shock-protein-70Bc**	121,3	39,9	30,9	-1,60	-1,97	-0,37	1,43E-01	2,83E-01	response to hypoxia /// response to hypoxia /// response to stress /// response to unfolded protein /// response to heat /// response to heat /// heat shock-mediated polytene chromosome puffing	nucleotide binding /// ATP binding
**Heat-shock-protein-70Bc /// Heat-shock-protein-70Bb /// Heat-shock-protein-70Ba /// Hsp70Bbb**	178,8	65,6	59,0	-1,45	-1,60	-0,15	8,66E-02	3,75E-01	response to hypoxia /// response to hypoxia /// response to stress /// response to unfolded protein /// response to heat /// response to heat /// response to heat /// heat shock-mediated polytene chromosome puffing	nucleotide binding /// ATP binding
**Heat-shock-protein-70Ab /// Heat-shock-protein-70Aa**	302,8	223,2	140,4	-0,44	-1,11	-0,67	1,28E-01	2,09E-01	response to hypoxia /// response to hypoxia /// response to stress /// response to unfolded protein /// response to heat /// response to heat /// response to heat /// heat shock-mediated polytene chromosome puffing	nucleotide binding /// protein binding /// ATP binding
**Glial Lazarillo**	108,8	68,6	50,2	-0,66	-1,12	-0,45	2,07E-03	4,76E-02	response to hypoxia /// response to stress /// response to stress /// determination of adult lifespan /// response to starvation	binding /// binding
**CG6673**	89,6	52,0	43,9	-0,79	-1,03	-0,25	3,81E-02	1,87E-01	response to oxidative stress /// metabolic process /// determination of adult lifespan	glutathione transferase activity /// glutathione transferase activity /// glutathione transferase activity /// pyrimidodiazepine synthase activity /// pyrimidodiazepine synthase activity /// protein binding /// transferase activity
**methuselah-like 2**	144,8	44,0	39,2	-1,72	-1,89	-0,17	1,01E-02	2,67E-01	response to stress /// response to stress /// signal transduction /// cell surface receptor linked signaling pathway /// G-protein coupled receptor protein signaling pathway /// G-protein coupled receptor protein signaling pathway /// determination of adult lifespan	signal transducer activity /// receptor activity /// transmembrane receptor activity /// G-protein coupled receptor activity /// G-protein coupled receptor activity
**inscuteable**	132,2	88,2	45,8	-0,58	-1,53	-0,94	8,19E-04	3,49E-02	RNA localization /// cytoskeleton organization /// neuroblast fate determination /// peripheral nervous system development /// sensory organ development /// somatic muscle development /// protein localization /// protein localization /// asymmetric protein localization /// asymmetric cell division /// regulation of asymmetric cell division /// establishment of mitotic spindle localization /// establishment of mitotic spindle localization /// establishment of mitotic spindle localization	binding /// protein binding /// cytoskeletal adaptor activity /// cytoskeletal adaptor activity /// cytoskeletal adaptor activity
**CG16790**	20,5	11,4	13,9	-0,84	-0,56	0,28	1,95E-01	3,52E-01	RNA metabolic process	catalytic activity /// protein binding
**Ecdysone-induced gene 71Ed**	1843,5	463,2	660,3	-1,99	-1,48	0,51	5,76E-02	2,38E-01	salivary gland cell autophagic cell death /// autophagic cell death	---
**Oseg4**	30,0	20,1	14,8	-0,58	-1,02	-0,44	2,31E-02	5,18E-02	sensory cilium assembly /// cilium assembly /// cilium assembly	binding
**lectin-28C**	362,6	255,9	178,8	-0,50	-1,02	-0,52	2,90E-02	1,06E-01	signal transduction /// G-protein coupled receptor protein signaling pathway /// G-protein coupled receptor protein signaling pathway /// visual perception /// phototransduction /// phototransduction /// phototransduction /// phototransduction /// adult locomotory behavior /// photoreceptor cell morphogenesis /// response to light intensity /// protein-chromophore linkage /// rhabdomere development /// negative regulation of compound eye retinal cell programmed cell death /// response to stimulus	signal transducer activity /// receptor activity /// G-protein coupled receptor activity /// G-protein coupled receptor activity /// binding /// protein binding /// galactose binding /// G-protein coupled photoreceptor activity /// G-protein coupled photoreceptor activity /// photoreceptor activity
**Ras-related protein**	502,9	377,7	229,4	-0,41	-1,13	-0,72	4,86E-03	1,56E-01	signal transduction /// small GTPase mediated signal transduction /// border follicle cell migration /// dorsal closure /// innate immune response /// negative regulation of JNK cascade	nucleotide binding /// GTPase activity /// GTPase activity /// GTPase activity /// protein binding /// GTP binding
**nessy**	574,6	316,4	262,2	-0,86	-1,13	-0,27	2,50E-03	1,02E-01	sperm individualization /// germ cell migration	---
**Stellate 12D orphon**	31,1	15,1	9,2	-1,05	-1,76	-0,71	2,53E-02	1,56E-01	spermatogenesis /// regulation of protein kinase activity	protein kinase regulator activity /// protein kinase regulator activity
**longitudinals lacking**	32,6	17,0	15,1	-0,94	-1,11	-0,17	9,74E-03	1,42E-01	startle response /// inter-male aggressive behavior /// transcription /// regulation of transcription from RNA polymerase II promoter /// multicellular	nucleic acid binding /// DNA binding /// RNA polymerase II transcription factor activity /// specific RNA polymerase II transcription factor activity /// specific
**olf186-F**	112,9	33,8	23,3	-1,74	-2,28	-0,54	5,60E-03	3,02E-02	store-operated calcium entry /// transport /// ion transport /// calcium ion transport /// nervous system development /// regulation of calcium ion transport via store-operated calcium channel activity /// positive regulation of calcium ion transport via store-operated calcium channel activity /// positive regulation of NFAT protein import into nucleus /// positive regulation of calcium ion transport	serine-type endopeptidase activity /// ion channel activity /// calcium channel activity /// store-operated calcium channel activity /// store-operated calcium channel activity /// hydrolase activity
**Tachykinin**	22,8	12,0	8,1	-0,93	-1,50	-0,57	3,22E-02	1,55E-01	tachykinin receptor signaling pathway /// neuropeptide signaling pathway /// neuropeptide signaling pathway /// behavior	receptor binding /// neuropeptide hormone activity /// neurotransmitter transporter activity /// protein binding
**sugarbabe**	165,1	36,8	51,6	-2,16	-1,68	0,49	2,81E-02	1,30E-02	transcription /// multicellular organismal development /// negative regulation of transcription /// regulation of transcription /// positive regulation of transcription	nucleic acid binding /// DNA binding /// zinc ion binding /// transcription activator activity /// metal ion binding
**buttonhead**	34,9	14,5	16,0	-1,27	-1,13	0,14	1,30E-02	3,53E-01	transcription /// regulation of transcription from RNA polymerase II promoter /// cephalic furrow formation /// specification of segmental identity, head /// head segmentation /// anterior head segmentation /// posterior head segmentation /// regulation of transcription /// positive regulation of transcription, DNA-dependent /// positive regulation of transcription from RNA polymerase II promoter	nucleic acid binding /// DNA binding /// RNA polymerase II transcription factor activity /// protein binding /// transcription factor binding /// zinc ion binding /// transcription activator activity /// metal ion binding
**cap-n-collar**	826,0	384,5	269,5	-1,10	-1,62	-0,51	4,03E-03	1,04E-01	transcription /// regulation of transcription, DNA-dependent /// multicellular organismal development /// oocyte dorsal/ventral axis specification /// regulation of pole plasm oskar mRNA localization /// blastoderm segmentation /// oocyte microtubule cytoskeleton polarization /// regulation of bicoid mRNA localization /// maintenance of oocyte nucleus location involved in oocyte dorsal/ventral axis specification /// regulation of transcription /// bicoid mRNA localization /// pole plasm oskar mRNA localization	DNA binding /// DNA binding /// sequence-specific DNA binding transcription factor activity /// sequence-specific DNA binding transcription factor activity /// RNA polymerase II transcription factor activity /// protein binding /// transcription activator activity /// sequence-specific DNA binding /// protein heterodimerization activity /// protein dimerization activity
**midline**	115,6	54,9	48,1	-1,07	-1,26	-0,19	5,64E-02	2,88E-01	transcription /// regulation of transcription, DNA-dependent /// regulation of transcription	DNA binding /// sequence-specific DNA binding transcription factor activity
**ventral veins lacking**	57,2	52,9	26,8	-0,11	-1,09	-0,98	4,12E-02	5,86E-02	transcription /// regulation of transcription, DNA-dependent /// regulation of transcription, DNA-dependent /// brain development /// peripheral nervous system development /// peripheral nervous system development /// epithelial cell fate determination, open tracheal system /// motor axon guidance /// brain segmentation /// regulation of transcription /// dendrite morphogenesis	DNA binding /// DNA binding /// sequence-specific DNA binding transcription factor activity /// sequence-specific DNA binding transcription factor activity /// RNA polymerase II transcription factor activity /// protein binding /// transcription regulator activity /// sequence-specific DNA binding
**Ecdysone-induced protein 75B**	108,8	98,9	51,3	-0,14	-1,09	-0,95	1,99E-02	2,38E-02	transcription /// regulation of transcription, DNA-dependent /// regulation of transcription, DNA-dependent /// multicellular organismal development /// regulation of ecdysteroid metabolic process /// molting cycle, chitin-based cuticle /// molting cycle, chitin-based cuticle /// ecdysis, chitin-based cuticle /// antimicrobial humoral response /// ecdysone-mediated induction of salivary gland cell autophagic cell death /// regulation of transcription /// oogenesis	DNA binding /// sequence-specific DNA binding transcription factor activity /// specific RNA polymerase II transcription factor activity /// steroid hormone receptor activity /// receptor activity /// ligand-dependent nuclear receptor activity /// ligand-dependent nuclear receptor activity /// ligand-dependent nuclear receptor activity /// zinc ion binding /// heme binding /// sequence-specific DNA binding /// metal ion binding
**Checkpoint suppressor homologue**	81,3	78,0	40,5	-0,06	-1,01	-0,95	3,82E-02	5,50E-02	transcription /// regulation of transcription, DNA-dependent /// regulation of transcription, DNA-dependent /// phagocytosis, engulfment /// regulation of transcription	DNA binding /// sequence-specific DNA binding transcription factor activity /// sequence-specific DNA binding transcription factor activity /// binding /// sequence-specific DNA binding
**CG17327**	88,9	37,9	34,4	-1,23	-1,37	-0,14	2,38E-02	3,29E-01	translation	aminoacyl-tRNA hydrolase activity
**mitochondrial ribosomal protein S28 /// mRpS28**	175,3	81,3	82,7	-1,11	-1,08	0,02	2,00E-02	3,81E-01	translation	structural constituent of ribosome
**CG4630**	70,4	42,4	30,9	-0,73	-1,19	-0,46	9,14E-03	1,75E-01	transmembrane transport	carnitine transporter activity
**CG3649**	41,3	14,2	16,2	-1,54	-1,35	0,19	4,59E-03	1,27E-01	transmembrane transport	high affinity inorganic phosphate:sodium symporter activity /// protein binding
**CG14511**	34,5	19,6	15,7	-0,81	-1,13	-0,32	1,06E-02	9,35E-03	transmembrane transport	UDP-N-acetylglucosamine transmembrane transporter activity

**CG5973**	82,7	49,2	23,2	-0,75	-1,83	-1,08	1,05E-03	1,55E-02	transport	transporter activity /// protein binding /// retinal binding
**CG3091**	48,0	35,5	23,5	-0,43	-1,03	-0,60	5,19E-03	1,52E-02	transport	transporter activity /// vitamin E binding
**ripped pocket**	26,4	7,3	7,4	-1,85	-1,83	0,02	9,18E-03	4,51E-01	transport /// ion transport /// sodium ion transport	ion channel activity /// sodium channel activity /// sodium channel activity /// sodium channel activity /// ligand-gated sodium channel activity
**Histamine-gated chloride channel subunit 1**	45,6	13,6	15,4	-1,75	-1,57	0,18	9,45E-03	1,85E-01	transport /// ion transport /// thermotaxis	ion channel activity /// extracellular ligand-gated ion channel activity /// extracellular-glycine-gated ion channel activity /// histamine-gated chloride channel activity
**Lipid storage droplet-1**	1314,9	1322,9	636,3	0,01	-1,05	-1,06	6,64E-05	5,57E-03	transport /// lipid transport /// lipid transport /// lipid storage /// lipid storage	protein binding
**CG8193**	304,4	44,0	50,9	-2,79	-2,58	0,21	1,99E-02	3,16E-01	transport /// metabolic process /// melanin biosynthetic process /// oxidation reduction	monooxygenase activity /// monophenol monooxygenase activity /// monopheno monooxygenase activity /// oxygen transporter activity /// oxidoreductase activity /// metal ion binding
**Black cells**	215,1	22,3	24,8	-3,27	-3,12	0,15	2,71E-02	3,78E-01	transport /// metabolic process /// oxidation reduction	catechol oxidase activity /// monophenol monooxygenase activity /// monophenol monooxygenase activity /// oxygen transporter activity /// oxidoreductase activity
**CG33282**	105,6	76,0	18,7	-0,47	-2,50	-2,03	8,89E-03	5,31E-03	transport /// monosaccharide transport /// transmembrane transport	transporter activity /// monosaccharide transmembrane transporter activity
**CG8850**	179,6	60,1	70,5	-1,58	-1,35	0,23	7,07E-03	1,88E-01	transport /// neurotransmitter transport /// neurotransmitter transport	neurotransmitter transporter activity /// neurotransmitter:sodium symporter activity /// symporter activity /// potassium:amino acid symporter activity
**CG1967**	142,7	147,2	48,7	0,04	-1,55	-1,60	3,15E-02	3,99E-02	transport /// post-Golgi vesicle-mediated transport	transporter activity
**Odorant-binding protein 50d**	27,5	2,0	9,7	-3,78	-1,50	2,28	2,41E-02	2,42E-02	transport /// sensory perception of chemical stimulus	odorant binding /// odorant binding
**Odorant-binding protein 83ef**	366,6	146,2	140,4	-1,33	-1,38	-0,06	3,26E-03	4,10E-01	transport /// sensory perception of chemical stimulus /// sensory perception of chemical stimulus	odorant binding /// odorant binding /// odorant binding /// odorant binding
**Odorant-binding protein 56a**	106,1	49,3	48,7	-1,10	-1,12	-0,02	1,93E-02	4,52E-01	transport /// sensory perception of chemical stimulus /// sensory perception of chemical stimulus /// sensory perception of smell /// sensory perception of smell /// response to pheromone /// olfactory behavior /// response to stimulus	protein binding /// odorant binding /// odorant binding /// odorant binding /// odorant binding
**Epidermal stripes and patches**	226,8	99,2	102,2	-1,19	-1,15	0,04	1,61E-02	3,41E-01	transport /// sulfate transport /// transmembrane transport	transporter activity /// secondary active sulfate transmembrane transporter activity /// secondary active sulfate transmembrane transporter activity
**CG5805**	700,7	331,4	346,6	-1,08	-1,02	0,06	3,16E-02	3,65E-01	transport /// transmembrane transport	transporter activity /// binding /// transmembrane transporter activity
**CG8323**	72,3	40,4	35,1	-0,84	-1,04	-0,20	1,12E-02	1,90E-01	transport /// transmembrane transport	transporter activity /// binding /// transmembrane transporter activity
**CG8654**	157,8	50,1	68,2	-1,66	-1,21	0,45	1,28E-03	1,64E-01	transport /// transmembrane transport	transporter activity /// secondary active organic cation transmembrane transporter activity
**CG7342**	262,5	146,1	125,3	-0,85	-1,07	-0,22	1,70E-03	1,74E-01	transport /// transmembrane transport	transporter activity /// secondary active organic cation transmembrane transporter activity
**CG6836**	24,6	7,7	5,6	-1,67	-2,13	-0,46	7,41E-04	8,07E-02	transport /// transport	transporter activity
**lethal (1) G0155**	160,1	55,1	64,4	-1,54	-1,31	0,22	1,54E-03	2,72E-02	transport /// vesicle-mediated transport /// vesicle-mediated transport	SNAP receptor activity
**fat facets**	23,6	3,9	2,5	-2,61	-3,24	-0,63	2,34E-02	3,07E-01	ubiquitin-dependent protein catabolic process /// ubiquitin-dependent protein catabolic process /// endocytosis /// nuclear migration /// multicellular organismal development /// cellularization /// visual perception /// germ cell migration /// mystery cell fate differentiation /// mystery cell fate differentiation /// embryo development /// protein deubiquitination /// protein deubiquitination	ubiquitin thiolesterase activity /// ubiquitin-specific protease activity /// ubiquitin-specific protease activity /// binding /// protein binding /// peptidase activity /// cysteine-type peptidase activity /// hydrolase activity
**CG13030 /// GA11985**	76,8	11,8	20,8	-2,71	-1,89	0,82	1,66E-03	1,72E-01	ubiquitin-dependent protein catabolic process /// ubiquitin-dependent protein catabolic process /// multicellular organismal development /// sensory organ development /// proteasomal protein catabolic process /// protein ubiquitination /// regulation of R7 cell differentiation	ubiquitin-protein ligase activity /// protein binding /// protein binding /// zinc ion binding /// ligase activity /// ligase activity /// metal ion binding
